# Mitochondrial adaptation in cancer drug resistance: prevalence, mechanisms, and management

**DOI:** 10.1186/s13045-022-01313-4

**Published:** 2022-07-18

**Authors:** Ping Jin, Jingwen Jiang, Li Zhou, Zhao Huang, Edouard C. Nice, Canhua Huang, Li Fu

**Affiliations:** 1 State Key Laboratory of Biotherapy and Cancer Center, West China Hospital and West China School of Basic Medical Sciences and Forensic Medicine, Sichuan University and Collaborative Innovation Center for Biotherapy, Chengdu, 610041 People’s Republic of China; 2 Department of Biochemistry and Molecular Biology, Monash University, Clayton, VIC 3800 Australia; 3 Guangdong Provincial Key Laboratory of Regional Immunity and Diseases, Department of Pharmacology and International Cancer Center, Shenzhen University Health Science Center, Shenzhen, 518060 Guangdong People’s Republic of China

**Keywords:** Cancer drug resistance, Mitochondrial dynamics, Mitochondrial adaptation, Drug repurposing, Mitochondrial-targeted drug delivery, Mitochondrial transplantation

## Abstract

Drug resistance represents a major obstacle in cancer management, and the mechanisms underlying stress adaptation of cancer cells in response to therapy-induced hostile environment are largely unknown. As the central organelle for cellular energy supply, mitochondria can rapidly undergo dynamic changes and integrate cellular signaling pathways to provide bioenergetic and biosynthetic flexibility for cancer cells, which contributes to multiple aspects of tumor characteristics, including drug resistance. Therefore, targeting mitochondria for cancer therapy and overcoming drug resistance has attracted increasing attention for various types of cancer. Multiple mitochondrial adaptation processes, including mitochondrial dynamics, mitochondrial metabolism, and mitochondrial apoptotic regulatory machinery, have been demonstrated to be potential targets. However, recent increasing insights into mitochondria have revealed the complexity of mitochondrial structure and functions, the elusive functions of mitochondria in tumor biology, and the targeting inaccessibility of mitochondria, which have posed challenges for the clinical application of mitochondrial-based cancer therapeutic strategies. Therefore, discovery of both novel mitochondria-targeting agents and innovative mitochondria-targeting approaches is urgently required. Here, we review the most recent literature to summarize the molecular mechanisms underlying mitochondrial stress adaptation and their intricate connection with cancer drug resistance. In addition, an overview of the emerging strategies to target mitochondria for effectively overcoming chemoresistance is highlighted, with an emphasis on drug repositioning and mitochondrial drug delivery approaches, which may accelerate the application of mitochondria-targeting compounds for cancer therapy.

## Background

Chemotherapy and targeted therapy are mainstream cancer treatments, but their efficiency is limited by frequent drug resistance and tumor relapse [1–3]. Generally, cancer drug resistance can result from two types of mechanism: intrinsic or acquired causes [4–8]. Intrinsic resistance is due to preexisting resistance-mediating factors prior to any treatment administered, while acquired drug resistance is caused by adaptive responses that confer cancer cell survival in unfavorable environments during drug treatment [9–12]. These adaptive response mechanisms include reduced uptake of drugs or increased drug efflux, ineffective induction of cell death, and compensatory activation of pro-survival signaling pathways [13–15]. Moreover, it is increasingly recognized that drug resistance can generally arise from a minor resistant subpopulation of cancer cells due to the high incidence of tumor heterogeneity. Recent studies have demonstrated that cancer stem cells (CSCs) are prone to maintain a quiescent state to evade the drug cytotoxicity which contributes to the development of a whole resistance phenotype [16–19]

Cancer cells often reprogram their metabolic pathways to provide energetic and biosynthetic flexibility to survive in hostile conditions when exposed to cancer treatments [20–26]. Metabolic reprogramming is considered as one of the major hallmarks of cancer [27] and has been an area of accelerated research over the last century on the basis of aerobic glycolysis theory proposed by Otto von Warburg, which describes the preference for glycolysis by tumors in the presence of oxygen [28–37]. While numerous studies have well documented the crucial role of metabolic adaptations in supporting cancer progression under endogenous stress such as hypoxia, cancer cells also develop metabolic flexibility to survive in response to exogenous stress including drug administration [28, 38–40]. Chemoresistance caused by glucose metabolic plasticity, for example, is generally mediated by several key glycolytic factors, such as Hexokinase 2 (HK2), glucose transporter 1 (GLUT1), as well as pyruvate kinase isozymes M2 (PKM2) [41–44]. The augmentation of glycolysis results in enhanced secretion of lactate and production of glycolytic intermediates, which activate branching pathways (e.g., pentose phosphate pathway (PPP)) and the stress response machinery to support nucleotide synthesis and redox homeostasis, leading to escape from apoptosis and reduction in drug entry [45, 46]. Correspondingly, targeting the dynamic adaptability of metabolism has obtained considerable effect in improving the efficiency of cancer therapy [47–50].

**Fig. 1:**
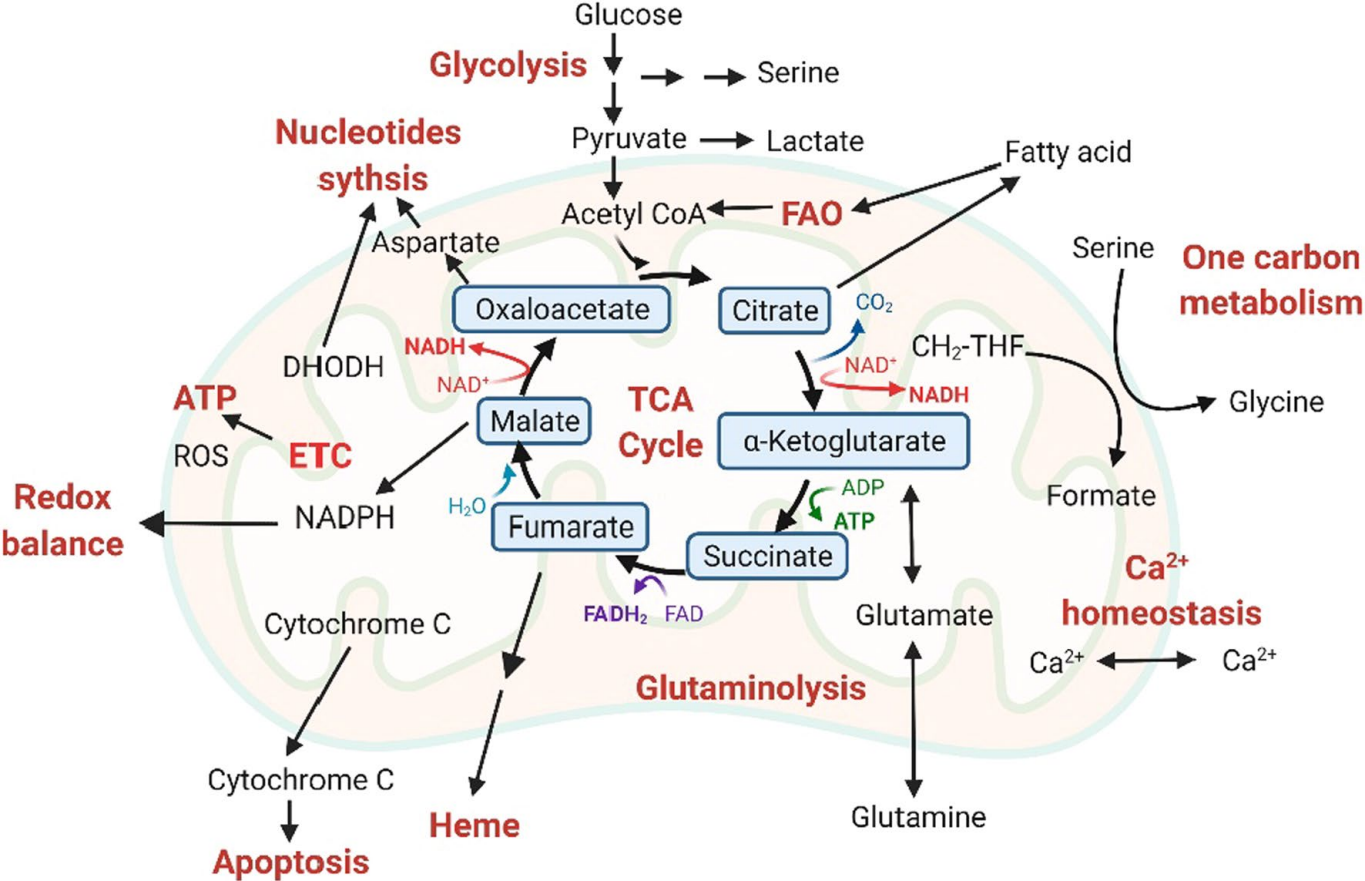
Mitochondria are energetic and biosynthetic signaling hubs. Mitochondria take up substrates from the cytoplasm to provide bioenergetic and biosynthetic flexibility. The TCA cycle coordinates glycolysis and glutaminolysis to provide blocks necessary for macromolecule (nucleotides, lipids, and amino acids) synthesis. This process produces ATP, NADPH, as well as the electron donors in OXPHOS (NADH and FADH2). The ETC complexes produce the majority of cellular ATP and oxidize NADH and FADH2 to NAD^+^ and FAD, respectively, to allow the oxidative TCA cycle to continuously function, producing metabolites that support macromolecule synthesis. DHODH couples de novo pyrimidine synthesis to donate electrons to mitochondrial ubiquinone (CoQ) during the conversion of dihydroorotate to orotate. Mitochondrial dynamics facilitate maximum survival advantages of cancer cells in response to stress by maintaining mitochondrial metabolism, ion homeostasis such as Ca^2+^ signaling, and redox balance

Mitochondria are the major organelles that provide bioenergetic and biosynthetic changes, which accompany tumor progression by taking up substrates from the cytoplasm to drive the electron transport chain (ETC) and respiration, the tricarboxylic acid cycle (TCA cycle), fatty acid oxidation (FAO), and subsequent macromolecule synthesis (Fig. 1) [51–54]. Additionally, mitochondria can rapidly sense and adapt to stress stimulation to ensure cell survival. Advanced studies on cancer metabolism have expanded our understanding of mitochondrial metabolic alterations to support anabolic requirements of cancer cells, which depend largely on the strictly intertwined plasticity of mitochondria (mitochondrial dynamics), including fusion/fission, trafficking/transfer, and inter-organelle communication/retrograde signaling [55]. While participating in the maintenance of cellular homeostasis during tumor progression, these mitochondrial adaptive processes are also pivotal for handling drug-induced stress, which contributes to alterations in mitochondrial metabolism and subsequent drug resistance [56–59]. For example, mitochondrial fission provides an advantage for cisplatin-resistant cells compared with their nonresistant counterpart under hypoxic conditions in ovarian cancer [60, 61]. In melanoma, the increased oxidative phosphorylation (OXPHOS) in resistant subclones is supported by peroxisome proliferator-activated receptorγcoactivator-1 (PGC-1α) and is required for buffering oxidative stress [46]. Indeed, mitochondria have received increasing attention as a therapeutic target for cancer therapy, and several agents targeting mitochondrial metabolism are under investigation [62–64]. However, the dynamic alterations and inaccessible characteristics of mitochondria make it a priority to explore novel mitochondria-targeting agents and strategies.

Here, we address the most recent findings regarding mitochondrial dynamics to indicate their functions in cancer drug resistance. An overview of existing mitochondrial agents will be presented, and emerging strategies for effective tumor elimination by targeting mitochondria, including drug repurposing and mitochondrial-targeted drug delivery systems, will be summarized.

## Mitochondrial structure and functions

Mitochondria are one of the most evolutionary conserved intracellular organelles that consist of the outer mitochondrial membrane (OMM) and a highly folded inner mitochondrial membrane (IMM) [65]. These two membranes are separated by the mitochondrial intermembrane space (IMS) and differ from each other in lipid composition and permeability. Importantly, the IMM invaginates into the mitochondrial matrix to form cristae, the crucial structure for mitochondrial function [66]. To maintain these structures, mitochondria undergo multiple complex processes (including fission and fusion) to dynamically control the function of mitochondria under various stimuli [67].

The primary function of mitochondria is demonstrated as energy supply. During the process of energy production, mitochondria integrate several metabolic pathways, including TCA cycle, FAO, amino acid oxidation, OXPHOS, etc., to provide not only most of the cellular ATP but also intermediate metabolite, supporting multiple physiological functions of the organism [68]. In recent decades, numerous studies have demonstrated that mitochondria also function as a signaling organelle to participate in many physiological processes, such as Ca^2+^ homeostasis, redox homeostasis, apoptosis regulation, and synthesis of heme and iron–sulfur clusters [69]. Indeed, mitochondria regulate Ca^2+^ homeostasis by exporting Ca^2+^ absorbed from intracellular store or extracellular uptake, which release Ca^2+^ back to the cytosol for regulation of calcium-dependent signaling [70]. In addition, the by-products of electron transfer during mitochondrial respiration result in the generation of reactive oxygen species (ROS), in which complexes I and III play the central role [71]. These physiological ROS together with the reducing equivalents (NADPH, etc.) generated by mitochondrial metabolism maintain the redox homeostasis for normal biological functions [72]. Moreover, mitochondria are tightly associated with apoptosis induction, as the release of cytochrome c, the key event of intrinsic apoptotic pathway, is mediated by the mitochondrial outer membrane permeabilization (MOMP) [73]. Therefore, the above complicated functions of mitochondria require the sophisticated regulation of mitochondrial dynamics for maintaining normal physiological functions of the organism.

Mitochondrial defects caused by various stimuli may lead to various pathologies, including neurodegenerative diseases, aging, and especially cancer. A large number of studies have suggested that mitochondria dysfunction may promote cancer onset and progression mainly through the following crucial mechanisms. First, as mitochondria are the major source of intracellular ROS, adequate levels of reactive species not only enable the accumulation of oncogenic defects of genes but also favor the activation of several oncogenic signaling pathways, which result in aberrant cell proliferation [74]. In addition, metabolic pathways in mitochondria may lead to the abnormal accumulation of specific metabolites, such as α-ketoglutarate (α-KG), pyruvate, fumarate, and succinate, which display significant oncogenic role during cancer initiation and progression [75–78]. Moreover, alterations or functional defects in MOMP are beneficial for survival of tumor cells when facing harsh conditions (such as hypoxic stress, metabolic stress, and therapeutic stress), thereby resisting regulated cell death [79]. During the dissemination and colonization, mitochondria endow metastatic cancer cells with phenotypic and metabolic plasticity for survival in intravascular transit and distant sites [80]. Taken together, the complex structures confer the diverse functions of mitochondria, whose dysfunction may regulate several aspects of cancer onset and progression, indicating a promising therapeutic target.

## Mitochondrial stress adaptation and drug resistance

**Fig. 2:**
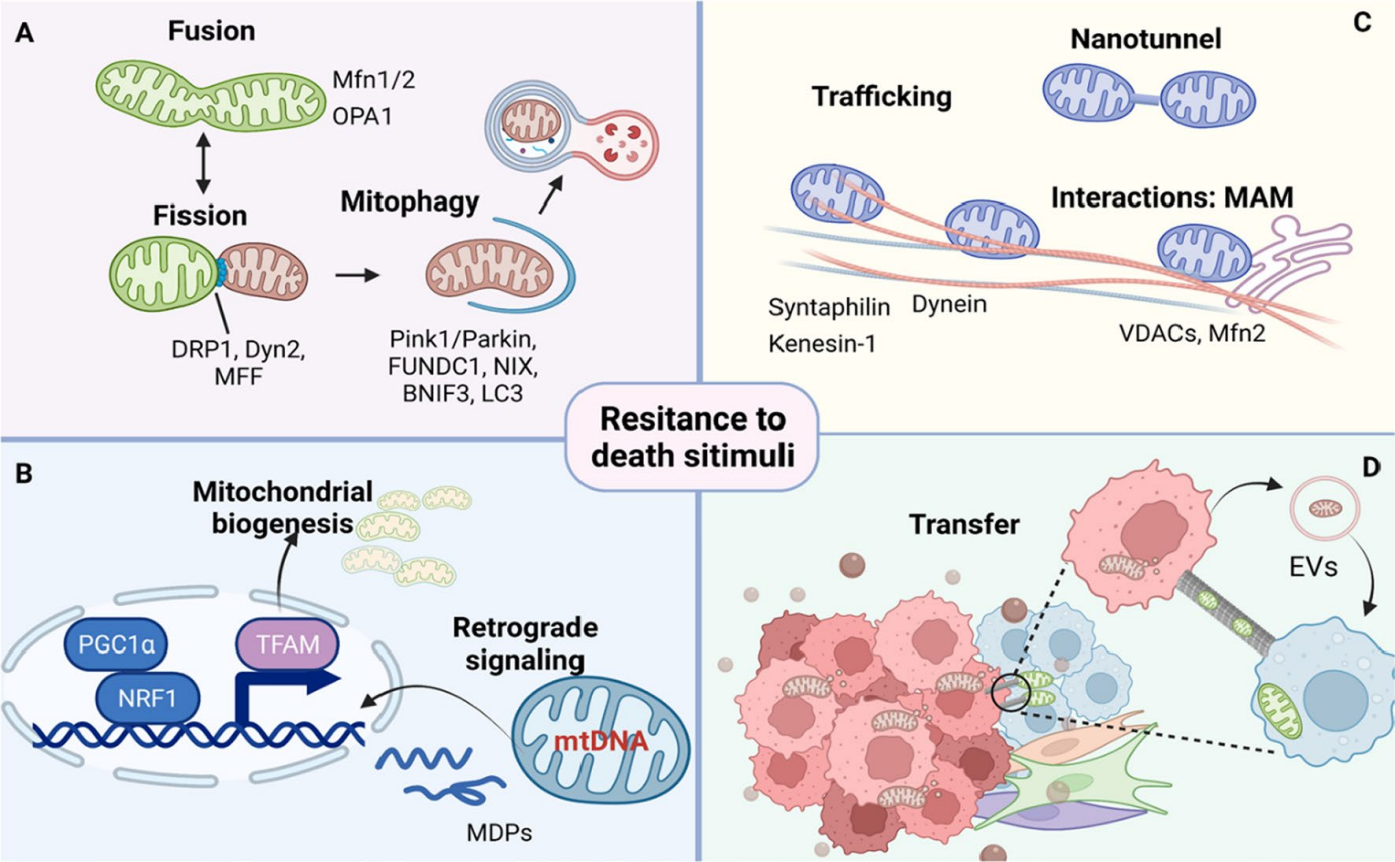
Mitochondrial stress adaptation and drug resistance. Mitochondrial dynamics are processes related to mitochondrial stress adaptation. These processes maintain proper mitochondrial numbers, structure, and position to ensure their function and could foster cancer drug resistance. (**A**) Fusion and fission allow mitochondria to constantly form networks or fragments according to cellular metabolic requirements. Mitophagy has been shown to coordinate with fission, facilitating the elimination of excessive or defective mitochondria. (**B**) While mitochondrial biogenesis and functions are largely regulated by nuclear coding factors, recent advances have revealed that mitochondrial dysfunction activates retrograde (mitochondria-to-nucleus) signaling to modify nuclear gene expression and subsequent cell behavior. This mitochondrial retrograde signaling functions as an adaptive mechanism for tumor cells to sense and mitigate mitochondrial stress. (**C**) Reshaping, localization, and motility of mitochondria along the microtubules facilitate mitochondria tethering with the ER or other organelles. (**D**) Recently described nanotunnel formation promotes component exchange and transfer of intercellular mitochondria, which usually increase OXPHOS output and ATP production of recipient cells and confer them with a survival advantage

Mitochondrial metabolic plasticity contributes to resistance in most types of anticancer therapy, as emphasized above [81, 82]. It is well orchestrated as a prerequisite of maintenance of OXPHOS, balance of ROS for signaling or defense, Ca^2+^ homeostasis, and proper induction of the apoptotic cascade. Mitochondrial dynamics modulate their shape, number, quality, and distribution in response to treatment and allow the maintenance of functional mitochondria (Fig. 2) [83]. Mitochondrial biogenesis and turnover, fusion and fission are universal mitochondrial stress-adaptive processes and have been well demonstrated to be involved in cancer drug resistance. Recent advances have expanded the paradigm of mitochondrial dynamics into mitochondrial trafficking and transfer, mitochondrial interplay with other organelles, and mitochondrial retrograde signaling [55, 84, 85]. These processes provide mitochondria plasticity for tumor cells, enabling tumor cells to survive under stress conditions, including radiotherapy and chemotherapy. In addition, the membrane system is essential for mitochondrial integrity to make the mitochondrial network more efficient in providing energy and required macromolecules. In this section, we systematically review the engagement of mitochondrial dynamics in cancer drug resistance.

### Mitochondrial biogenesis and turnover in drug resistance

Mitochondrial biogenesis and turnover are two opposing processes that work in concert to regulate mitochondrial mass, function, and quality regulating the biogenesis of new mitochondria and the removal of damaged mitochondria in a time-dependent manner. Mitochondrial biogenesis is regulated by the coordinated transcription of mitochondrial nuclear genes, in which PGC-1α plays a central regulatory role [86]. Reduced cellular bioenergetic output usually triggers mitochondrial biogenesis by activating AMPK to furnish OXPHOS and ATP production. In addition, oncogenes such as K-Ras and C-Myc are also involved in regulating mitochondrial biogenesis and increasing intracellular biosynthesis and respiration, thereby promoting tumorigenesis [87]. In particular, c-Myc controls the transcription of approximately 400 mitochondrial-related genes, thus regulating mitochondrial biogenesis [88]. These transcription networks provide metabolic flexibility for cancer cells to facilitate their adaptation to a hostile microenvironment and ultimately reduce the effectiveness of tumor treatment.

The most well-studied regulator involved in tumor drug resistance is PGC-1α, which facilitates tumor cell survival and metastasis under environmental stress by mediating mitochondrial biogenesis and OXPHOS [89, 90, 92]. Previous studies found that mutations in B-Raf or N-Ras in melanoma confer chemoresistance to MEK inhibitors by switching the metabolic mode to OXPHOS through upregulating PGC-1α or TFAM (transcription factor A, mitochondrial) to meet their bioenergy requirements [91, 93]. Similarly, upregulation of mitochondrial biogenesis and OXPHOS could augment tolerance to stimuli such as radiotherapy and ultraviolet radiation [94]. These studies demonstrate the critical role of adaptive mitochondrial biogenesis in drug-resistant capacity and highlight the potential of targeting mitochondrial OXPHOS for improving drug efficacy.

The maintenance of mitochondrial quality is also ensured by mitophagy, a programmed degradative process for eliminating excessive or defective mitochondria. Generally, the “eat me” signal on damaged mitochondria directly triggers mitophagy machinery by membrane depolarization and a cascade of phosphorylation and ubiquitination events to remove cytotoxic cellular components and maintain energy balance in the cell. It shares a common core mechanism with macro-autophagy but depends on specific mitophagy receptors, including the classical PTEN-induced putative kinase 1 (PINK1)-Parkin pathway [95, 96], and several other receptors, including Bcl-2/adenovirus E1B 19 kDa interacting protein 3 (BNIP3/NIX), FUN14 domain-containing protein 1 (FUNDC1), and Bcl-2-like protein 13 (BCL2L13). While these receptors have been well demonstrated to elicit mitophagy and facilitate tumor progression [97–99], they were proven to confer resistance of cancer cells to a variety of commonly used chemotherapy drugs, such as 5-fluorouracil (5-fu), cisplatin, and doxorubicin (Dox), by triggering mitophagy [64, 100, 101]. For example, the highly activated ATAD3A-PINK1/Parkin signaling pathway under hypoxic conditions confers tolerance of liver cancer cells to sorafenib [102]. In esophageal squamous cell carcinoma (ESCC) patients, PINK1-mediated mitophagy promotes tumor cell survival under neoadjuvant therapy [103]. Interestingly, PINK1 could recruit ARIH1, rather than Parkin, to trigger mitophagy and ultimately lead to drug resistance in breast and lung cancer cells [104]. Several other emerging receptors, such as FUNDC1 and Galectin-1, have been reported to be upregulated to promote resistance to cisplatin and ionizing radiation by eliciting mitophagy [105]. In addition, the upregulation of these receptors relies on a series of stress-adaptive transcriptional programs mediated by p53, NF-κB, and STAT1/2 [106]. It would be of interest to further explore the regulatory role of these networks in regulating mitophagy and their potential role in drug resistance.

### Mitochondrial fusion and fission in drug resistance

Mitochondrial fusion and fission allow mitochondria to constantly form networks or fragments according to cellular metabolic requirements. In general, fusion is commonly triggered by huge energy requirements, mediated by dynamin-related GTPases, optic atrophy 1 (OPA1) for IMM and mitofusin (MFN) 1 and 2 for OMM, resulting in a hyperfused mitochondrial network with increased mtDNA integrity, mitochondrial respiration, ATP production, and mitochondrial membrane potential (MMP) [107, 108]. Mitochondrial fission is mainly mediated by dynein-related protein 1 (DRP1) and fission protein homologous protein 1 (FIS1) and has been shown to coordinate with mitophagy or apoptosis, facilitating the elimination of damaged mitochondria, the redistribution of mtDNA, and the mobility of mitochondria [109–113]. These two opposing processes are tightly organized in response to stressors, thus engaging in tumor progression in a context-dependent manner [114–116]. For instance, the increase in mitochondrial fission caused by hypoxia has been shown to enhance the invasion of breast cancer [117].

Recent advances in mitochondrial medicine highlight the engagement of mitochondrial fusion and fission in mediating metabolic adaptation to chemotherapeutic agents in tumor cells. The relevance of mitochondrial fusion in chemoresistance is primarily evidenced by the upregulation of OPA1 and MFN1/2 expression, as well as interconnected mitochondrial networks in drug-resistant cancer cells. For instance, upregulated OPA1 confers resistance to cytochrome c release upon prolonged venetoclax treatment in acute myeloid leukemia (AML) cells [118]. Consistently, upregulated MFN2 and increased OXPHOS have been found in cancer cells that survive chemotherapy [119, 120]. Several other factors have been reported to promote cancer drug resistance by triggering mitochondrial fusion. For example, circulating leptin protein activates induced myeloid leukemia cell differentiation protein (MCL1, a member of the anti-apoptotic protein BCL2 family) to induce mitochondrial fusion, thereby promoting tumor cells to survive during gemcitabine treatment [121].

Increasing evidence also shows the pivotal role of mitochondrial fission in chemoresistance [60, 122]. One of the best examples is that phosphorylation of DRP1 induces mitochondrial fragmentation to promote metabolic adaptation, thus protecting cancer cells from chemotherapy agents [123–125]. Phosphorylation or activation of several upstream kinases, such as AMPK, cyclin B1/Cdk1, ERK1, and DRP1, is involved in mitochondrial fission-mediated chemoresistance [61, 126]. Since mitochondrial fusion and fission represent two opposing systems, their balance and role in cellular fate are carefully orchestrated by specific cellular metabolic requirements. Therefore, more insights into the regulatory patterns of mitochondrial fusion and fission and their effect in chemotherapy are necessary to develop therapies offering improved clinical outcomes for cancer patients.

### Inter-organelle contact sites, mitochondrial trafficking, and transfer in drug resistance

Mitochondria dynamically form contacts with various intracellular organelles to maintain cell homeostasis by fine-tuning Ca^2+^ transfer, phospholipid biosynthesis, ROS signaling, mitochondrial quality control, and mtDNA synthesis [127–133]. Such cellular membrane interactions are extensive and play the essential role in cell adaptation to metabolic stress [134].

The mitochondrial-associated endoplasmic reticulum (ER) membrane (MAM) is the most well-studied membranous system coordinating with a series of proteins and factors to maintain proper mitochondrial Ca^2+^ uptake which correlates with resistance to chemotherapy [135]. Mitochondrial Ca^2+^ uniporter complex (MCUC) subunits (MCU, MICU1, MICU2, EMRE, and MCUb) cooperate to maintain mitochondrial Ca^2+^ homeostasis, and their relevance to drug resistance is condition dependent [136–139]. For example, downregulation of MCU was demonstrated to confer resistance by restricting the transport of Ca^2+^ to the mitochondria in HeLa cells [140]. However, the interaction of MCU with receptor-interacting protein kinase 1 (RIPK1) can increase mitochondrial Ca^2+^ uptake, resulting in increased proliferation of colorectal cancer cells [141]. Additionally, MCUR1-mediated mitochondrial Ca^2+^ signaling was reported to facilitate cell survival of hepatocellular carcinoma (HCC) upon pro-apoptotic stimuli [137]. Several other recognized mitochondrial proteins, such as MFN2 and voltage-dependent anion-selective channel proteins (VDACs), were identified as important MAM proteins that might be involved in tethering MAMs and facilitating mitochondrial fission, mitophagy, and mitochondrial positioning [142, 143]. Moreover, there is mitochondrial contact with other organelles, such as peroxisomes, and lipid droplets, in response to metabolic stress. For example, “lipid droplet mitochondria” can help mitochondria provide energy by burning fatty acids (FAs) to support the TCA cycle [144]. Therefore, we conclude that exploring mitochondria-associated tethering systems could expand the understanding of mitochondrial dynamics and provide new targets for tumor intervention.

The proper localization of organelles is often crucial to their activity and function [145]. Numerous studies have shown that the movement and subcellular location of mitochondria can affect tumor cell polarity, morphology, and mobility capacity [146–150]. Mitochondrial stress responses drive strategic mitochondrial redistribution to fulfill bioenergetic needs, Ca^2+^ homeostasis, ROS buffering, and signal transduction, thus promoting the adaptation of tumor cells to the harsh tumor microenvironment [150–156]. A typical example is that of mitochondria migrating to the invasive front of metastatic tumor cells [157]. For example, the NF-κB-inducing kinase (NIK)-DRP1 axis could mediate the fission and subsequent directionally positioning of mitochondria to the cell periphery to promote the migration of a variety of tumors [151, 158]. In addition, the trafficking of mitochondria along the cytoskeleton could protect cells from detrimental ROS production [155, 159]. Notably, mitochondrial trafficking occurs to endow tumor cells for survival and metastasis upon drug abuse [160]. For instance, activated Akt promotes mitochondria positioning along the cytoskeleton to provide an effective “regional” energy source, thus fueling resistance and even adaptive cell invasion in response to PI3K inhibitors [148]. Intensive studies on this “spatiotemporal” model of mitochondria may deepen our understanding of the subcellular accumulation of mitochondria as an adaptative process and may provide a viable strategy to increase anticancer efficacy in the clinic.

Recent studies have shown that mitochondrial dynamics are accompanied by intercellular mitochondrial transfer [161–163]. This transfer process is achieved through several mechanisms, including gap junctions, extracellular vesicles (EVs), and tunneling nanotubes (TNTs) [164–169]. TNTs, the transient cytoplasmic extensions, are the major cellular structure that mediate intercellular mitochondrial transfer [170]. The mitochondrial transfer process usually increases the OXPHOS output and ATP level of recipient cells. As a consequence, recipient cancer cells exhibit a survival advantage and resistance to stress [171–173]. Moschoi et al. observed that AML cells acquire intact mitochondria from marrow stromal cells (MSCs) to maintain their own mitochondrial function and survive during cytarabine treatment [174]. Several other tumors can also despoil mitochondria from MSCs and obtain resistance to chemotherapeutics [172]. In addition, it has been proposed that mitochondria transfer from bone marrow stromal cells (BMSCs) to multiple myeloma (MM) cells, which can also contribute to chemoresistance [175, 176]. Consistently, in breast cancer, mitochondrial transfer promotes resistance to doxorubicin [177], and mtDNA in exosomes derived from hormonal therapy-resistant breast cancer cells leads to endocrine therapy resistance. Intercellular transfer of mitochondria expands the influence of mitochondria on tumor metabolism, suggesting that targeting mitochondrial transfer could represent a more reasonable and effective antitumor strategy.

### Mitochondrial retrograde signaling in drug resistance

Mitochondrial dynamic changes are positively regulated by nuclear coding factors, while recent advances underline that retrograde signaling activated by mitochondrial dysfunction can modify nuclear gene expression and subsequent cell behavior [59, 178]. In fact, it serves as an adaptive mechanism for tumor cells to sense and mitigate mitochondrial stress, thus participating in tumor survival, metastasis, and drug resistance.

#### Retrograde reactions

The signals from mitochondrial dysfunction, especially mutation/deletion in mtDNA, are usually relayed to the nucleus by TCA cycle intermediates, ATP, Ca^2+^ or ROS, which activate specific kinases to initiate transcriptional regulation of nuclear genes or posttranslational modification of key proteins (e.g., histone acetylation) [179–181]. The most well-studied example is the activation of AMPK triggered by a decrease in ATP levels, which elicits PGC-1α-mediated transcription of genes responsible for energy metabolism, mitochondrial synthesis, and the quality control system [182, 183].

As mentioned above, mitochondria are essential for maintaining intracellular Ca^2+^ levels. Disruption of MMP caused by deletion of the electron transport chain complex or drug insult led to leakage of Ca^2+^ into the cytoplasm. Intracellular free Ca^2+^, on the one hand, activates multiple oncogenic signaling pathways, including RAC-alpha serine/threonine-protein kinase (AKT) and phosphatidylinositol 3-kinase (PI3K), to upregulate the expression of glucose transporters, such as GLUT1 and GLUT4, thereby promoting the metabolic switch to glycolysis and the survival of cancer cells [184–186]. On the other hand, Ca^2+^ signaling activates NF-κB and T cell nuclear factor (NFATC) signaling to facilitate the transcription of Ca^2+^ transport and storage-related proteins [187, 188].

ROS can directly manipulate cellular redox homeostasis and act as second messengers to regulate cellular physiological and pathological processes [189–191]. For example, ROS elicited by mtDNA depletion could activate the NRF2 signaling pathway and the multidrug resistance proteins MRP1 and MRP2 to help tumor cells fight against ROS and survive under cisplatin, doxorubicin, and SN-38 treatment [192]. In addition, ROS modulate the expression of PGC-1α to promote OXPHOS, thus conferring cisplatin resistance in ovarian cancer cells [193]. Together, these studies have linked mtDNA mutations/deletion with changes in sensitivity of cancer cells to chemotherapy, thus providing a new perspective on modulating drug resistance.

#### Mitochondrial nuclear feedback and mitochondrial stress-relieving response

Mitochondria have evolved protein quality control systems to maintain mitochondrial integrity by ensuring proper folding, assembly, and circulation of mitochondrial proteins in response to exogenous or endogenous stressors. This process tightly relies on feedback regulatory loops, including the mitochondrial unfolded protein stress response (UPR^mt^) [194], proteolytic stress responses [195], and the heat shock response of mitochondrial chaperones [196]. These mechanisms are deregulated due to altered signaling to confer cancer cell survival, which ultimately contribute to tumor progression and drug resistance. This could be due to several proposed mechanisms, including continuous activation of NF-κB and molecular chaperone systems and mutations in the catalytic sites that contribute to resistance to proteasome inhibitors. For instance, a recent study has suggested that the mitochondrial oxidoreductase ferredoxin 1 (FDX1) maintains mitochondrial metabolism to promote the adaptation of tumor cells to the proteasome inhibitor elesclomol [197]. More investigations revealing the mechanisms employed in the quality control system of mitochondrial protein might offer unique strategies for improving therapeutic efficacy in cancer treatment.

#### Mitochondrial-derived peptides

Recently, mitochondria-derived peptides (MDPs, short open reading frames (sORFs) of mitochondrial genome) have been identified and implicated in stress response, metabolic regulation, and other biological processes. In response to cellular stress, these peptides can even directly manipulate nuclear gene expression [198], which expands the paradigm of mitochondrial nuclear communication. Several MDPs have been identified, including Humanin [199], humanin-like peptides (SHLP) 1–6 [200], and MOTS-c [201–203]. Among them, humanin was reported to protect cells from oxidative stress and mitochondrial dysfunction [204]. SHLP2 and SHLP3 exert similar cytoprotective effects by maintaining mitochondrial function and combating excessive ROS levels [202, 205]. In particular, MOTS-c was reported to translocate to the nucleus under metabolic stress such as glucose deprivation and oxidative stress [206]. In the nucleus, MOTS-c regulates the transcription of a broad range of genes, including those with antioxidant response elements (AREs) and other anti-inflammatory-associated genes, to initiate the stress adaptation program. A considerable number of studies have now proven that MDPs are intrinsically linked to tumor progression, and targeting MDPs holds potential to improve the efficacy of chemotherapeutics [207, 208].

### Mitochondria-mediated CSC properties in drug resistance

It is well established that CSCs contribute substantially to the refractory features of cancer. Under pharmacological treatment, mitochondria function as a central hub to maintain the survival and self-renewal capacity of CSCs, resulting in drug resistance and tumor recurrence [209]. For example, it has been reported that oncogenic Myc cooperated with MCL1 to maintain chemoresistance of CSCs in triple-negative breast cancer (TNBC). Further studies found that Myc and MCL1 upregulated the levels of mitochondrial OXPHOS and promoted ROS generation, which contributed to the accumulation of HIF-1α and the subsequent maintenance of CSC properties [210]. Moreover, PGC-1α, a critical regulator of mitochondrial biogenesis, has been demonstrated to enhance stem cell-like characteristics and chemoresistance to cisplatin in ovarian cancer [211]. In addition, increased levels of mitochondrial mass were found in a subtype of chemo-resistant breast cancer cells enriching in several known CSC markers, implying the potential of targeting mito-high CSC population for cancer therapy [212]. Therefore, investigation of mitochondrial function in regulating CSCs holds the promise to benefit the development of novel CSC-targeted strategies for reversing cancer drug resistance.

In summary, a large body of evidence has indicated the important role of mitochondrial dynamics in the adaptative mechanism of cancer cells in response to a challenging environment, which is expected to expand the understanding of cancer drug resistance phenomena [91, 213–216]. Further investigations deciphering specific mitochondrial-related mechanisms implicated in the resistance could hopefully benefit the identification of possible biomarkers for the early prediction of cancer drug resistance and hold promise to target mitochondria for overcoming cancer drug resistance.

## Targeting mitochondria to overcome cancer drug resistance: the current status and challenges

**Fig. 3:**
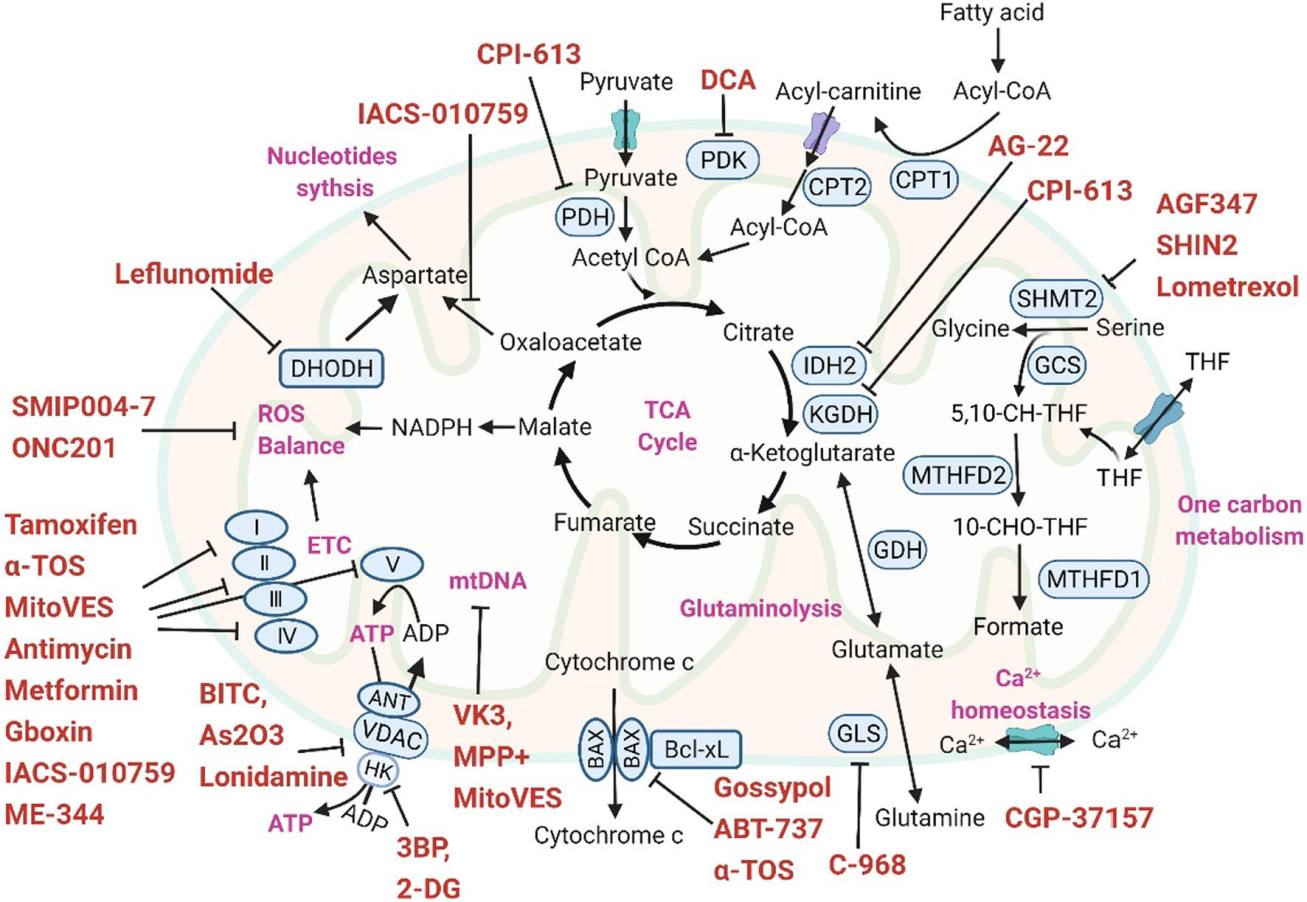
Schematic showing representative mitochondrial therapeutic targets and their proposed inhibitors. The mitochondrial ETC, as the central system of mitochondrial energy production and OXPHOS, has been the most frequently used mitochondrial target. Multiple mitochondrial ETC inhibitors, including metformin and IACS-010759, are being investigated for cancer treatment. In addition, inhibitors targeting different steps of the mitochondrial TCA cycle have shown promise in phase I and II clinical trials. Several other TCA cycle-coupled biomacromolecule synthesis pathways, such as the nucleotide synthesis pathway and the one-carbon (1C) metabolic pathway, are also potential therapeutic targets for reversing drug resistance

The mitochondrial ETC fuels cellular energy demands by utilizing intermediates from various metabolic pathways, including the TCA cycle and FAO, and couples the generation of macromolecules such as amino acids and nucleotides [217–219]. Thus, dynamic regulation of mitochondria involves robust metabolic and redox alterations, as well as changes in ion (e.g., Fe^2+^, Ca^2+^) homeostasis. Increasing knowledge that these critical processes are linked to tumor transformation makes mitochondria an attractive therapeutic target. In this section, we will summarize current mitochondrial therapeutic targets and their proposed inhibitors (Fig. 3, Table 1), with a particular emphasis on the role of small-molecule inhibitors in targeting mitochondria to overcome cancer chemoresistance.

**Table 1:** Summary of mitochondrial targets for cancer therapy

Category (Mitochondria signaling pathway)	Targets	Inhibitors and Refs
Mitochondrial ETC and OXPHOS	Complex I	Piericidin [220]; rotenone [221]; deguelin [222]; tamoxifen [123, 223]; metformin [224]; ME-344 [225]
	Complex II	α-TOS [226]; MitoVES [227]
	Complex III	Resveratrol [228];
	Complex IV	Fenretinide [229];
	Complex V	BZ-423 [230]
Targeting the mitochondrial metabolic pathway	Heme synthesis	Cyclopamine tartrate (CycT) [231]; HasA [232]
	1C metabolism (SHMT1 and SHMT2)	AGF291, AGF320, and AGF347 [233, 234]; lometrexol [235]
	DHODH	leflunomide [236, 237]
	Nucleotide biosynthesis	IACS-010759 [238]
	TCA cycle (α-ketoglutarate dehydrogenase (α-KGDH) and pyruvate dehydrogenase (PDH)	CPI-613 [239]
	Glutaminase (GLS)	CB-839 [240], bis-2-(5-phenylacetamido-1,2,4-thiadiazol-2-yl) ethyl sulfide (BPTES) [241]
	Glutamate-pyruvate transaminase 2 (GPT2)	Aminooxyacetate (AOA) [54]
Redox balance	NADH:ubiquinone oxidoreductase	SMIP004-7 [242];
Ca^2+^ homeostasis	VDACs and ANT	lonidamine[243], steroid analogs [244]
	Na^+^/Ca^2+^ exchanger	CGP-37157 [245]
	Bcl-2	Gossypol [246]
	S100A4	Niclosamide [247]
Mitochondrial membrane proteins	Hexokinase II	3-bromopyruvate [248]; 2-Deoxyglucose (2-DG) [249]

### Targeting mitochondrial ETC

The mitochondrial ETC complexes I–V (hereafter CI-V) bypass electrons to generate energy and are the major source of mitochondrial ROS [250, 251]. Disruption of the ETC inevitably triggers apoptosis and perturbs the cellular redox balance and therefore provides a possible strategy for eliminating cancer cells [252].

#### Complex I

Mitochondrial complex I (CI), the largest complex of the ETC, transfers TCA cycle-derived electrons from NADH from the UbQ and maintains the proton gradient on the MIM. Several inhibitors including piericidin, tamoxifen, metformin, and ME-344 that directly target the respiratory complex I have gained momentum as potential antitumor therapeutics. Metformin, an antidiabetic drug has now been repurposed as an anticancer drug and was observed to inhibit CI. Many preclinical and clinical studies have demonstrated its excellent antitumor efficacy in managing resistance caused by chemotherapeutics, including cisplatin, Dox, and 5-FU (NCT00897884, NCT02437656; Table 2) [253–256]. In addition, a recent phase II clinical trial observed that combinational use of metformin with standard epidermal growth factor receptor-tyrosine kinase inhibitor (EGFR-TKI) therapy significantly improved both progression-free survival and overall survival in patients with advanced lung adenocarcinoma (NCT03071705; Table 2) [257]. Consistently, metformin-sensitized lymphoma to isocitrate dehydrogenase (IDH) mutant inhibitors and increased the sensitivity of lymphoma to AZD3965, a monocarboxylate transporter (MCT1) inhibitor, by disturbing mitochondrial complex I and bioenergetics, thus providing a scientific rationale for combinatory mitochondrial-targeted therapies to overcome drug resistance in human lymphoma [53, 258].

**Table 2:** Clinical trials of identified mitochondrial inhibitors

Inhibitor	Target	Cancer type	ClinicalTrials.gov Identifier	Status
Metformin	CI	Breast cancer	NCT04559308	Recruiting
		Breast cancer	NCT00897884	Completed
		Rectal cancer	NCT02437656	Completed
		etc.		
VLX600	ETC	Refractory cancer	NCT02222363	Terminated
Fenretinide	CIV	Advanced or metastatic hormone refractory prostate cancer	NCT00077402	Completed
		Lung cancer	NCT00009971	Completed
		Bladder cancer	NCT00004154	Completed
		etc.		
Resveratrol	CIII	Colon cancer	NCT00256334	Completed
		Liver cancer	NCT00433576	Completed
		Breast cancer	NCT03482401	Completed
		etc.		
Tigecycline	Mitochondrial ribosomal machinery	Acute myeloid leukemia	NCT01332786	Completed
Gamitrinib	Mitochondrial chaperone proteins, such as TRAP-1 and HSP-90	Lymphoma, advanced solid tumor	NCT04827810	Recruiting
CPI-613	TCA cycle, PDH	Biliary tract cancer	NCT04203160	Recruiting
		Advanced hematologic malignancies	NCT01034475	Completed
		Recurrent small cell lung cancer	NCT01931787	Completed
		etc.		
Dichloroacetate	PDK	Head and neck cancer	NCT01163487	Completed
		Squamous cell carcinoma of the head and neck	NCT01386632	Completed
		etc.		
IACS-010759	CI, TCA cycle	Advanced malignant solid neoplasm; Anatomic stage IIIA breast cancer	NCT03291938	Completed
		Recurrent acute myeloid leukemia	NCT02882321	Active, not recruiting
		etc.		
Leflunomide	DHODH	Breast neoplasms	NCT03709446	Recruiting
		Prostate cancer	NCT00004071	Completed
		Brain and central nervous system tumors	NCT00003775	Completed
		etc.		
ONC201	OXPHOS	Endometrial cancer recurrent	NCT03485729	Recruiting
		Triple-negative breast cancer; endometrial cancer; hormone receptor positive, HER2 negative breast cancer	NCT03394027	Completed
		Recurrent neuroendocrine tumor; metastatic neuroendocrine tumor	NCT03034200	Active, not recruiting
		etc.		
Tamoxifen	ETC	Breast cancer	NCT00286117	Completed
		Estrogen receptor positive breast cancer	NCT02988986	Completed
		Bladder cancer	NCT02197897	Completed
ME-344	ETC	Breast cancer; human epidermal growth factor 2 negative carcinoma of breast; early-stage breast carcinoma	NCT02806817	Completed
		etc.		
Gossypol	Bcl-2 family proteins	Extensive stage small cell lung cancer; Recurrent small cell lung cancer	NC T00666666	Completed
		etc.		
Lometrexol	SHMT2	Lung cancer;	NCT00033722	Unknown
		Unspecified adult solid tumor, protocol specific	NCT00024310	Unknown

#### Complex II

Complex II (CII, succinate dehydrogenase), the smallest respiratory complexes, is a membrane-bound component of the TCA cycle that permits the oxidation of succinate to fumarate. Compounds that induce substantial ROS generation from CII are the emerging anticancer drugs. For example, alpha-tocopheryl succinate (α-TOS), a compound targeting UbQ-binding sites in CII, has been demonstrated to disturb CII for eliciting mitochondrial permeabilization and apoptosis, thus showing significant potential in overcoming drug resistance in various tumors [226, 259, 260]. In particular, based on the knowledge that α-TOS targets UbQ-binding sites, a more specific mitochondrial-targeted analogue, MitoVES, was designed for efficiently suppressing tumors by disturbing mitochondria [261, 262].

#### Complex III

CIII, similar to CI, functions by pumping protons across the MIM and contributing to the proton gradient. It has been also identified as target for anticancer drugs. Antimycin A is the most classic CIII inhibitor to trigger apoptosis for effectively eliminating cancer cells [263, 264]. Resveratrol, a plant-derived polyphenol, exhibited considerable antitumor efficacy by efficiently inhibiting ETC complexes, especially CIII to induce apoptosis and disturb multiple cellular processes in primary and resistant cancer cells [265–267]. Importantly, the anticancer potential of resveratrol is being investigated in clinical trials for the treatment of colorectal, liver, and breast cancer (NCT0025633, NCT00433576, and NCT03482401; Table 2).

#### Complex IV

CIV ensures the final step of electron transport in ETC to maintain the proton gradient and mitochondrial membrane potential. Several compounds have been observed to modulate the mRNA or protein expression of CIV subunits and thereby induce apoptosis in tumor cells. Fenretinide (also named as N-(4-hydroxyphenyl) retinamide, 4-HPR) and its analogue are perhaps the most well-documented category to downregulate CIV by destabilizing the mRNA transcript [268], thereby inducing ROS-mediated apoptosis to combat tumors in both preclinical and clinical studies (NCT00004154, NCT00009971, and NCT00077402; Table 2) [269, 270]. In particular, Fenretinide has been reported to eliminate ABT-737-resistant cell lines via ROS generation and MCL1 reduction and thus has synergetic effect with ABT-737 to enhance mitochondrial apoptotic cascade in acute lymphoblastic leukemia (ALL) [271].

#### Complex V

CV, the ATP synthase, directly catalyzes ATP production using the proton gradient maintained by complexes I–IV, thus supplying the cell with essential energy. Besides oligomycin and its derivatives, several newly identified CV inhibitors, including 3,30-diindolylmethane (DIM), Bz-423, were proposed to fight tumors and even those with drug resistance [230, 272]. Notably, there are compounds that could inhibit different complexes of ECT. For example, resveratrol is also reported to bind to CV and induce Bcl-2-mediated apoptosis [273]. In addition, Gboxin, an OXPHOS inhibitor, is observed to interact with several respiratory chain proteins spanning CI, CII, CIV, and CV, thereby suppressing tumor growth [274]. Although it remains to be known whether these ETC inhibitors will be effective in humans, emerging studies provide excellent prospects for their application in cancer therapy and drug resistance eradication.

#### Mitochondrial redox balance

ROS are intrinsically involved in tumor progression by modulating cell survival, secondary signaling networks, and genetic instability/mutations [275]. Mitochondria function as a major contributor to endogenous ROS due to the large electron flow in the ETC and constant metabolism alterations involved in numerous enzyme-catalyzed reactions. Mitochondrial redox balance is typically mediated by cellular antioxidants, such as glutathione (GSH), glutathione peroxidases (GPx1 and GPx4), and glutathione reductase [276–282]. Emerging observations suggest that heightened levels of ROS contribute to drug resistance. For instance, gefitinib resistance was demonstrated to be associated with mitochondrial dysfunction in lung cancer cells [283]. Conventional chemotherapies, such as 5-FU and cisplatin, are designed to kill cancer cells via ROS-dependent mechanisms. In that context, enhanced ROS levels could maximize antitumor efficacy. For example, SMIP004-7 targets NADH:ubiquinone oxidoreductase to improve the immunotherapeutic effect of PD-1 in triple-negative breast cancer [242]. Destroying the redox balance is perhaps the essence for modulating mitochondrial ROS to benefit cancer therapy. In view of this, redox status during treatment and the basal mitochondrial ROS range may provide important clues for guiding rational intervention strategies. Selective targeting of ROS-specific organelles, as well as dynamic ROS delivery, might be beneficial for preventing drug resistance and effectively eliminating cancer cells.

### Targeting the mitochondrial metabolic pathway

#### Nucleotide biosynthesis

Targeting mitochondrial ETC-linked metabolic pathways, such as nucleotide metabolism, also contributes to improved antitumor efficacy. One-carbon (1C) metabolism coordinates with serine synthesis to provide glycine and tetrahydrofolate methyl donors, namely methylene-THF (5,10-CH-THF) and formyl-THF (10-CHO-THF), for nucleotide synthesis. As essential enzymes in 1C metabolism, cytosolic SHMT1 and mitochondrial SHMT2 have attracted much attention. Several inhibitors, including AGF291, AGF320, and AGF347, have been developed to target these enzymes, and their antitumor efficacy has been established for lung, colon, and pancreatic cancer cells [233, 234, 284]. Intriguingly, folate inhibitors such as lometrexol have also been found to reduce SHMT1 and SHMT2 activity [235]. Several clinical trials are undergoing to investigate the use of lometrexol in advanced solid tumor (NCT00033722 and NCT00024310; Table 2).

In addition, efforts have been made to inhibit nucleotide metabolic enzymes, such as the intramitochondrial key pyrimidine synthesis-related enzyme, dihydroorotate dehydrogenase (DHODH). Leflunomide exhibited antitumor activity in prostate cancer mouse model by inhibiting DHODH [236]. Additionally, a phase I clinical study showed leflunomide had considerable activity toward myeloma with manageable side effects (NCT02509052; Table 2). Furthermore, IACS-010759, a mitochondrial CI inhibitor, was developed to induce apoptosis in brain cancer and AML, likely caused by energy depletion and reduced aspartate production that led to impaired nucleotide biosynthesis [238]. Combinational use of IACS-010759 with lactate dehydrogenase (LDH) inhibitor could overcome oxidative rewiring and show a synergistic therapeutic effect [49]. Key enzymes involving in nucleotide metabolism could also play a “part-time role” in other mitochondrial processes (e.g., SHMT2 participating in mitochondrial translation) [285–287]. The antitumor effects of nucleotide metabolism inhibitors could be manifold. As such, further efforts are urgently needed to screen for candidates targeting nucleotide metabolism for cancer management.

#### TCA cycle

The mitochondrial TCA cycle integrates multiple fuel sources to synthesize nucleotide, amino acid, lipid, and heme. Several TCA cycle inhibitors have been under investigation and predicted to be efficacious. Among them, CPI-613, a lipoate analog that targets two enzyme complexes of the TCA cycle (α-ketoglutarate dehydrogenase (α-KGDH) and pyruvate dehydrogenase (PDH)) [288], exerts anticancer activity in pancreatic cancer and AML [289]. Notably, CPI-613 could sensitize AML cells to cytarabine and mitoxantrone, representing a promising approach for relapsed or refractory AML [239]. To date, clinical trials of CPI-613 for the treatment of advanced or recurrent tumor are ongoing, or have already been completed with satisfactory results (NCT04203160, NCT01034475, and NCT01931787; Table 2). Interestingly, a recent study reported that vitamin C modulates the activity of PDH and regulates the TCA cycle via interfering with PDK1-mediated phosphorylation of PDH in KRAS mutant colon cancer, suggesting a potential application for clinical management of chemoresistance to anti-EGFR therapy [290].

#### Glutaminolysis

Glutamine (Gln) can be converted to glutamate by glutaminase (GLS) and further metabolized to α-KG via glutamate dehydrogenase 1 (GLUD1), glutamate oxaloacetate transaminase 2 (GOT2) or glutamate-pyruvate transaminase 2 (GPT2), thus providing a major carbon source to replenish the TCA cycle [291–295]. Many classes of compounds that target mitochondrial Gln metabolism are being investigated for cancer treatment. GLS inhibitors have shown promising anticancer effect in preclinical models of cancer. For example, bis-2-(5-phenylacetamido-1,2,4-thiadiazol-2-yl) ethyl sulfide (BPTES), a GLS inhibitor, has been demonstrated to slow the growth of various types of tumors [296, 297].

Importantly, BPTES was observed to efficiently sensitize pancreatic cancer to 5-(tetradecyloxy)-2-furoic acid (TOFA, an acetyl-CoA carboxylase inhibitor) and ß-Lap (an NADPH:quinone oxidoreductase (NQO1) inhibitor) via enhancing cancer cell apoptosis [241, 298, 299]. In particular, CB-839 (telaglenastat), another GLS inhibitor, has moved on to clinical trials and exhibits promise as potential drug for renal cell carcinoma (NCT03428217; Table 2), hematological malignancies (NCT03428217 and NCT02071888; Table 2), non-small cell lung cancer (NSCLC) (NCT02071862; Table 2), and even those drug-resistant tumors (NCT03944902 and NCT03798678; Table 2). Recently, GPT2 has been demonstrated to promote cell survival by supporting the TCA cycle after GLS inhibition [54]. In that context, inhibition of GPT2 using aminooxyacetate (AOA), a transaminase inhibitor, could thus sensitize cancer cells to BPTES.

### Targeting Ca^2+^ homeostasis

Mitochondria have evolved Ca^2+^ influx and efflux systems to maintain cellular Ca^2+^ homeostasis. Proper mitochondrial Ca^2+^ ensures respiration efficacy and ATP production, while Ca^2+^ overload can induce mitochondria-mediated apoptosis [300–303]. Therefore, mitochondrial Ca^2+^ signaling pathways engage multifaceted roles in regulating cell fate and are beneficial for tumorigenesis. Studies are under way to identify the proteins involved in mitochondrial Ca^2+^ signaling pathways as alternative targets for cancer therapy, and to evaluate the potential for increasing the sensitivity toward chemotherapeutic treatment. Compounds that modulate mitochondrial porins such as VDACs and ANT, including lonidamine, arsenites, and steroid analogs, have been documented to disturb the mitochondrial Ca^2+^ balance and elicit mitochondrial apoptosis, thus showing potent antitumor efficacy as well as drug resistance overcoming activity [243, 244, 304]. In addition, a mitochondrial Na^+^/Ca^2+^ exchanger inhibitor, CGP-37157, resulted in a persistent mitochondrial Ca^2+^ rise and may serve as a promising agent to overcome TRAIL (tumor necrosis factor-related apoptosis-inducing ligand) resistance [245, 303]. In ovarian cancer cells, the overexpression of Bcl-2 attenuated cisplatin cytotoxicity by downregulating ER-mitochondrial Ca^2+^ signal transduction. Thus, targeting Bcl-2-mediated Ca^2+^ signal might be a potential approach to overcome drug resistance in ovarian cancer [305].

### Current challenges in targeting mitochondria

The mitochondrion is perhaps the most challenging target for cancer therapy [306, 307]. The constant alterations in mitochondrial structure and position contribute greatly to the failure of targeted agents, the bias in drug toxicity, and drug dosage prediction [308]. For example, the doses of metformin that could reduce the proliferation of cancer cells in laboratory models (in vitro cell lines and in vivo mouse models) were 10- to 1,000-fold higher than those that are deemed safe clinically [309]. This makes it urgent to assess the efficacy and safety of higher doses of metformin to determine its clinical potential. Second, many mitochondrial inhibitors are delivered into the mitochondria depending on the MMP. As such, imported drugs could inhibit ETC complexes and diminish the MMP, leading to decreased total agent importation [310, 311]. In addition, targeting the ETC is likely to be fraught with severe side effects. Some ETC inhibitors are considered neurotoxic, such as rotenone [312, 313], and some, such as cyanide, are even lethally toxic [314, 315]. In particular, IACS-010759, a CI inhibitor being advanced to clinical trials (NCT03291938 and NCT02882321; Table 2) [238, 316], has been associated with neuropathy and visual changes [49]. Moreover, metabolic plasticity promotes cancer cells to shift their metabolic features upon targeting specific metabolic vulnerabilities [317]. Furthermore, the selection of metabolic targets for therapeutic intervention has often been done in cell culture systems, where the metabolism of initial tumor-derived cells may be significantly affected by culture conditions [318]. These systems also do not recapitulate tumor heterogeneity and complex inter-tumor and tumor–host interactions [47].

## Strategies for overcoming mitochondria-targeting bottlenecks to combat drug resistance

As mentioned above, limitations in drug sources and drug targeting challenge the application of targeting mitochondria for improving therapeutic efficiency in cancer treatment. Therefore, researchers are seeking new strategies to achieve a competitive advantage in targeting mitochondria for cancer therapy. Redevelopment and reuse of old drugs (repurposing/repositioning) represent such an opportunity to replenish the inventory of mitochondrial-targeted antitumor drugs [224]. Besides, the use of targeted nanomedicines offers innovative therapeutic strategies to overcome multiple barriers and selectively transport drug molecules to the mitochondria [319, 320]. Additionally, mitochondrial transplantation is an emerging approach that exerts antitumor potential by restoring mitochondrial function [321].

### Drug repurposing for overcoming mitochondria-targeting bottlenecks

Drug repositioning is a strategy to identify medications that were initially used for the treatment of other noncancer illnesses for tumor therapy, based on an accumulated understanding of their mechanisms of action [322–324]. The advantages of this approach include, but are not limited to, the already established pharmacokinetic, pharmacodynamic, and toxicity profiles, their rapid progress into clinical trials, the significantly lower associated development cost as well as a relatively less risky business plan [325–327]. In recent years, technological innovation combined with the development of big data repositories and the analytical methods, as well as the emergence of a variety of innovative computational methods and in silico approaches, have greatly promoted the process of drug repurposing [328–331]. In this section, we present various promising repurposed non-oncology drugs that disrupt specific mitochondrial components and their functions for preclinical or clinical management of cancer drug resistance (Table 3).

**Table 3:** List of repurposed mitochondria-targeted drugs

Original application	Repurposed drug	Effects on mitochondria	Effects on cancer	Cancer type
Antidiabetes	Metformin	↓Complex I activity, ↓Respiration, ↓ATP production	↓Cell growth, ↑cell death, overcome chemoresistance	Breast cancer, colorectal cancer, lung cancer, ovarian cancer, etc.
	HL156A	↓MMP, ↑ROS, ↑caspase-3 and caspase-9	↑Apoptosis, antiproliferative, Radio sensitizing	Oral cancer
	Phenformin	↓Complex I, ↓respiration, ↓ATP production	Antiproliferative, radio sensitizing, overcome chemoresistance	Colorectal cancer, breast cancer
	Exendin-4 (Exe-4)	Mitochondrial dysfunction, ↑ROS,	Overcome chemoresistance	Endometrial cancer
	Canagliflozin	↓Complex I, ↓respiration	Antiproliferative, Radio sensitizing, overcome chemoresistance	Prostate cancer, lung cancer, etc.
	Pioglitazione	↓Oxygen consumption	Antiproliferative, overcome chemoresistance	Prostate cancer
Antibiotics	Tigecycline	↓mtDNA-encoded proteins, ↓Respiration	Overcome chemoresistance	Renal carcinoma, ovarian cancer
	Levofloxacin	Mitochondrial dysfunction, oxidative damage	Inhibit proliferation, induce apoptosis	Lung cancer
Anthelminthic	Niclosamide	Mitochondrial dysfunction, activated Bax and caspases-3	↑Apoptosis, ↓Migration, ↓Invasion	Thyroid cancer, chondrosarcoma tumor
	Ivermectin	↓Mitochondrial respiration, ↓Membrane potential, ↓ATP levels	Inhibit angiogenesis, ↓growth and survival	Glioblastoma
Antimalarial agents	Artemisinin	↓MMP, ↑ROS, ↑Ca^2+^	Cell cycle arrest, ↑Apoptosis, Anti-angiogenesis	Colorectal cancer, breast cancer
	Artesunate	Mitophagy, ↓GSH, ↑ROS	↑Cell death	Cervical cancer
Antifungal agents	Itraconazole	VDAC1 inhibition, mitochondrial metabolism disruption	Inhibit angiogenesis, ↓Growth and survival	Breast cancer, liver cancer
	Ketoconazole	Mitophagy	Antiproliferative, overcome chemoresistance	HCC
	Econazole	Ca^2+^ channel inhibition, cytochrome c leakage	↑Cell death, Anti-tumorigenesis	Leukemia, colorectal cancer
Antihypertension	Prazosin	↓MMP,	Reduced tumor mass	Prostate
	Quercetin	↓MMP,	Antitumor, Radio sensitizing	Lung cancer, gastric cancer
	Lercanidipine	Mitochondrial Ca^2+^ overload, mitochondrial vacuolation	↑Apoptosis, chemosensitization	Breast cancer
	Telmisartan	Mitochondrial fission, ROS accumulation	↑Apoptosis, chemosensitization	Melanoma
Antidepressants	Imipramine	Stressed mitochondria restoration	Hijack aggressive character of cancer	Glioblastoma
	Amitriptyline	Stressed mitochondria restoration	Hijack aggressive character of cancer	Glioma
	Chlorimipramine	↓Complex III activity, ↓MMP, mitochondrial swelling and vacuolation	Antitumor	Glioma
	Fluoxetine	Mitochondrial dysfunction	Antiproliferative, overcome chemoresistance	Colorectal cancer, breast, Ovarian cancer
	Depression	Mitochondrial dysfunction	↑Apoptosis,	Bladder cancer
Antiepileptic drug	Valproic acid	↓Respiration, ↓ATP production, ↑ROS	Antiproliferative, Pro-apoptotic, chemosensitization	Thoracic cancer, lung cancer, colorectal cancer
Treatment for pain	Aspirin	Activated Bax and caspases-3, cytochrome c leakage	↑Apoptosis	Cervical cancer
Treatment for rheumatism	Indomethacin	Impairs mitochondrial dynamics	↑Apoptosis, chemosensitization	Lung cancer, gastric cancer, etc.
	Auranofin	Inhibits mitochondrial TrxR	Antiproliferative	Lung cancer, ovarian carcinomas
Treatment for stomachache, abdominal pain, rheumatism	Angelica polymorpha maxim root extract	↓MMP, activated Bax and caspases-3	↑Apoptosis	Neuroblastoma
Treatment for rheumatism, Liver cirrhosis	Euphorbia formosana Hayata (EF)	Mitochondria dysfunction	Tumor suppression	Leukemic cells
Treatment for thalassemia, Friedreich’s ataxia kidney disease	Deferiprone	Suppress mitochondrial metabolism, ↓Respiration, ↑ROS	Antiproliferative, Reduce migration	Prostate, Breast cancer, etc.
Iron chelator	VLX600	↓Respiration, ↓ATP production	Antitumor, chemosensitization	Ovarian cancer, colorectal cancer, etc.
Copper overload disorder	Tetrathiomolybdate	↓Respiration, ↓ATP production	Inhibit angiogenesis, antitumor	Papillary thyroid cancer
Alcohol-aversion drug	Disulfiram	Mitochondrial fission, ↓MMP	Antitumor, chemosensitization	Melanoma, colorectal cancer
Copper-chelating agent	Elesclomol	Interacts with ETC, ↑ROS	↑Apoptosis	Colorectal cancer, leukemia, etc.
Palliative effects	Cannabinoids	Mitochondrial damage, ↑ROS	Reduce proliferation, induce apoptosis and autophagy, Inhibit invasion and angiogenesis, Improve chemosensitivity	Oral cancer, Lung cancer, etc.

#### Repositioning antidiabetic drugs

Metformin, an approved antidiabetic drug which has been used in cancer therapy, is one of the most successful repurposed drugs [332–335]. Several signaling pathways, including insulin/IGF1, NF-κB, AMPK/mTOR/PI3K, Ras/Raf/Erk, Wnt, Notch, and TGF-β signaling, have been identified to be involved in its antitumor effect [336–344]. Besides, metformin has been well demonstrated to target mitochondria and induce cytotoxic effects [345–348]. Numerous preclinical studies and clinical trials are investigating the therapeutic potential of metformin in many types of tumors [349–351]. Consistent with this, metformin was proven to enhance the anticancer effect of radio- or chemotherapies. For instance, Lee et al. observed that metformin could overcome resistance to cisplatin by downregulating RAD51 expression, representing a novel strategy in TNBC management [255]. In addition, in NSCLC, metformin acts synergistically with sorafenib to inhibit cell proliferation by activating AMPK, which holds significant potential to be tested in prospective clinical trials [352].

Other biguanides also exhibit enhanced antiproliferative or radio-sensitizing effects in cancer cells. For instance, HL156A, a metformin analog, markedly decreased MMP and induced ROS levels to activate caspase-3- and caspase-9-mediated apoptosis, thus suppressing tumor growth [353]. This study suggests the potential value of HL156A as a candidate for the treatment of oral cancer. Phenformin, a potent mitochondrial ETC inhibitor, also displayed remarkable anticancer activity against several tumors [354]. In colorectal cancer, phenformin could overcome hypoxic radio resistance through inhibition of mitochondrial respiration [355]. In breast cancer, phenformin synergistically decreased respiration and ATP production with oxamate, an inhibitor of lactate dehydrogenase, to inhibit tumor growth [356]. Furthermore, phenformin and oxamate displayed synergistic anticancer effects through simultaneously inhibiting mitochondrial complex I and cytosol LDH in this study.

Moreover, several other antidiabetic drugs have also been successively repurposed for cancer therapy and drug resistance management. Exendin-4 (Exe-4), a GLP-1 receptor agonist, was reported to elevate mitochondrial ROS and trigger subsequent apoptosis, which attenuated hyperglycemia-induced chemoresistance and sensitized human endometrial cancer cells to cisplatin treatment [357]. Canagliflozin, another antidiabetic drug, was identified to inhibit the proliferation of lung and prostate cancer cells, alone or in combination with ionizing radiation or chemotherapy using docetaxel by inhibiting mitochondrial CI supported respiration [358]. In addition, piperazine also targeted mitochondria to inhibit oxygen consumption, thus exhibiting an additive effect on inhibiting cell proliferation in combination with the glycolysis inhibitor 2-deoxyglucose (2-DG) [359].

Overall, repurposing antidiabetic drugs provides a plethora of candidates to suppress the growth of many types of tumors by targeting mitochondria. These drugs could not only increase the efficacy of standard therapies, but also reduce their side effects by potentially modulating metabolic plasticity.

#### Repositioning antimicrobial agents

Antimicrobial therapeutics, including antibiotics, anthelminthic and antifungal drugs, have been repurposed against tumors (e.g., breast, liver, colorectal and lung cancers, glioblastoma, multiple myeloma, and leukemia). A particularly important mechanism underlying their anticancer effects is interfering with mitochondrial function [360]. Examples of antibiotics that suppress cancer by altering mitochondria include the chloramphenicol family and tetracycline [361]. For instance, tigecycline preferentially inhibits the translation of mtDNA-encoded proteins to restrain the mitochondrial respiratory chain, causing mitochondrial dysfunction and increased oxidative stress, thus providing a therapeutic strategy for overcoming chemoresistance in human renal cell carcinoma and ovarian cancer [362, 363]. The antibiotic drug levofloxacin has also been repurposed to inhibit proliferation and trigger apoptosis of lung cancer cells by inducing mitochondrial dysfunction and oxidative damage [364].

In addition, several anthelminthic compounds were observed to interfere with mitochondria and combat with tumors. For instance, niclosamide could induce mitochondrial dysfunction and activate Bax and caspase-3, which attenuates migratory and invasive behaviors and promote apoptosis in thyroid cancer and chondrosarcoma tumors [365, 366]. Another anthelmintic drug, ivermectin, was suggested to inhibit angiogenesis, growth, and survival by decreasing mitochondrial respiration, membrane potential, and ATP levels [367]. The well-documented antimalarial agent artemisinin and its derivatives also possess potent anticancer activity through mitochondria-related pathways, manifesting as significantly reduced MMP, increased intracellular ROS and Ca^2+^ levels, and upregulated apoptosis-associated proteins [368, 369]. In particular, artesunate was reported to induce PINK1-dependent mitophagy to alter the cellular redox status in HeLa cells [370]. In addition, atovaquone, another antimalarial agent, can inhibit mitochondrial complex III, thereby increasing the efficacy of radiotherapy [371].

Antifungal agents also play an important role in drug repositioning strategies for the treatment of various tumors [372–374]. Itraconazole is among the most well-studied broad spectrum antifungal agents for cancer treatment [374–378]. It has been reported that itraconazole can interact with mitochondrial protein VDAC1 and modulate the AMPK/mTOR signaling axis [379, 380]. Another study has demonstrated that itraconazole elicited apoptosis by altering MMP, reducing Bcl-2 expression and elevating caspase-3 activity [381]. Our group previously found that ketoconazole, a P450 inhibitor traditionally used for antifungal treatment [382], elicited PINK1/Parkin-mediated mitophagy and apoptosis, thereby suppressing HCC growth alone or synergistically with sorafenib [383]. In addition, Econazole (Eco), a potent agent used for tackling superficial mycosis, is now well recognized as an antagonist for store-operated Ca^2+^ channels to induce cell death of leukemia [384–386]. Expectedly, it has now been shown to trigger mitochondrial-mediated apoptosis and cause cytochrome c leakage and apoptosis-inducing factor (AIF) translocation [387].

In conclusion, repurposing of broadly antimicrobial compounds emerges as an important strategy to provide complementary and alternative first-line drugs for effectively targeting mitochondria in cancer cells. We believe that repositioning antimicrobial agents will be an important topic in realizing the reversion of cancer drug resistance by eliciting mitochondria-dependent apoptosis.

#### Repositioning anti-cardiovascular disease drugs

Anti-cardiovascular disease drugs are another class of compounds that have attracted interest for their anticancer efficacy. An example is prazosin, an orally active postsynaptic selective alpha 1-adrenoreceptor antagonist used in treating hypertension, congestive heart failure (CHF), and even posttraumatic stress disorder (PTSD). It has been recognized to possess anticancer activity in some types of cancer by modulating the PI3K/Akt/mTOR signaling pathway [388]. Further, prazosin was demonstrated to intensify docetaxel-induced toxicity in prostate cancer cells [389]. In addition, another study demonstrated that prazosin triggers mitochondria-mediated caspase executing apoptotic pathways in PC-3 cells, thus significantly reducing tumor mass in PC-3-derived cancer xenografts [390]. Quercetin, a bioflavonoid with multiple activities including antihypertensive, and anti-inflammatory, has been repurposed for cancer treatment [391–393]. Accumulating studies have been devoted to exploring the molecular basis underlying the antitumor efficacy of quercetin. The decrease in MMP and subsequent apoptosis represent potential mechanisms [394–396]. Another antihypertensive drug, lercanidipine, was shown to induce apoptosis accompanied by severe vacuolation derived from the ER and mitochondria, thereby enhancing the cytotoxicity of various proteasome inhibitors, including bortezomib, carfilzomib, and ixazomib, in many solid tumor cells [397]. Furthermore, a widely used and safe antihypertensive drug, telmisartan, was suggested to alter cell bioenergetics by triggering mitochondrial fission and ROS accumulation, thereby sensitizing melanoma cells to treatment with vemurafenib [398].

Taken together, anti-cardiovascular disease drugs hold great potential to be endowed with novel characteristics to tackle tumors in a mitochondria-dependent way.

#### Repositioning antidepressant drugs and anti-neurodegenerative drugs

It has been increasingly recognized that antidepressant drugs exert anti-neoplastic properties, in addition to their well-documented ability to modulate neurotransmission [399–402]. Tricyclic antidepressants and their analogs are among the most well-studied repurposed drugs for cancer therapy. They have exhibited excellent efficacy in halting cancer cell growth and metastasis [403–406]. Interestingly, the antitumor efficacy of imipramine and amitriptyline primarily relies on their metabolic modulating ability in restoring the proper function of mitochondria, which differs from those functioning through disturbing mitochondria [407]. For instance, recent investigations showed that imipramine and amitriptyline restore stressed mitochondria and stimulate their function to hijack the aggressive character of cancer caused by mitochondrial dysfunction [408]. Chlorimipramine, another tricyclic antidepressant, has been shown to specifically inhibit mitochondrial CIII and cause decreased MMP as well as mitochondrial swelling and vacuolation, thus exhibiting a selective antitumor effect [409]. In addition, fluoxetine has been reported to increase doxorubicin accumulation within multiple drug-resistant (MDR) cells and inhibit drug efflux both in vivo and in vitro in resistant tumor models [410]. It is also able to induce mitochondria-mediated cell death in human epithelial ovarian cancer [411, 412]. Moreover, nortriptyline can induce both Fas, FasL, FADD axis-mediated extrinsic apoptosis and mitochondria dysfunction-triggered intrinsic apoptosis, thus suppressing bladder tumor growth in vivo [413].

Agents for treating neurodegenerative disease (such as Alzheimer’s and Epilepsy) have also been observed to be efficacious in the prevention and treatment of tumors [414, 415]. Valproic acid (VPA), an antiepileptic drug, has been shown to inhibit class I HDAC and exert antiproliferative, pro-apoptotic, and chemo-sensitizing effects in human lung cancer and colorectal cancer by restraining the cell cycle and eliciting ROS generation [416, 417]. In addition, VPA significantly induced mitochondrial dysfunction, thus reducing respiration and ATP production causing mitochondria-dependent apoptosis, which potentiated TRAIL-mediated cytotoxicity on cultured thoracic cancer and HCC cells [418, 419].

#### Repositioning anti-inflammatory and antirheumatic drugs

Nonsteroidal anti-inflammatory drugs (NSAIDs) (e.g., indomethacin, ibuprofen, aspirin, and diclofenac) are the most commonly prescribed compounds for treating pain and inflammation [420–422]. It is widely accepted that NSAIDs possess anti-neoplastic effects in a wide spectrum of cancers [423–426]. In fact, prolonged NSAID administration reduces the risk of developing tumors [427, 428], and these non-oncology drugs are now applied to combination therapeutic regimens to potentiate the efficacy of chemotherapy and radiotherapy [429]. Based on the inhibitory effect on prostaglandin-synthesizing cyclooxygenases 1 and 2 (COX-1/2) and the role of nonsteroidal anti-inflammatory drug-induced gene NAG-1 in initiating the intrinsic apoptosis pathway [430], the mechanism of action underlying their antitumor efficacy is strongly related to mitochondrial dysfunction and ROS production caused by inhibition of mitochondrial respiration [431, 432]. This is best exemplified by aspirin, an FDA-approved NSAID for the treatment of pain and fever [433, 434]. Studies focusing on its antitumor mechanisms revealed that aspirin causes cytochrome c leakage and induces caspase-dependent apoptosis in cancer cells [435]. This mitochondrial damage is also probably responsible for the circumvention of resistance and sensitization to cisplatin by asplatin, a Pt(iv) prodrug of cisplatin, due to the ligation of aspirin [436]. Indomethacin, another NSAID initially used for treating rheumatic disease, has been found to induce mitochondria-mediated apoptosis in doxorubicin-resistant lung cancer cells through an MRP1-dependent mechanism [437]. In addition, indomethacin can activate the PKCζ-p38-DRP1 pathway to impair mitochondrial dynamics, thus inducing apoptosis in gastric cancer [438].

Other groups of anti-inflammatory and/or antirheumatic drugs also exhibit antitumor efficacy. Auranofin, an inhibitor of thioredoxin reductase (TrxR) initially developed for the treatment of rheumatoid arthritis, exhibited anticancer activity against various tumor types. It was approved for clinical trials in lung and ovarian carcinomas [439–443]. Further investigations revealed that auranofin targets both the cytosolic and mitochondrial forms of TrxR, indicating that mitochondrial alterations might participate in the inhibitory effect of auranofin on cancer [444, 445]. In addition, Euphorbia formosana Hayata (EF), a Taiwanese medicinal plant for the treatment of rheumatism, has been repurposed for tumor suppression by eliciting apoptosis via the Fas and mitochondrial pathways in leukemic cells [446].

In summary, anti-inflammatory agents, pain-relieving medication, and antirheumatic drugs are now documented to be effective again diverse critical disorders including cancer, for which mitochondrial-related mechanisms are well recognized to be involved in their antitumor effects.

#### Repositioning ion chelating agents

Ion chelating agents represent a category of effective antitumor agents by targeting mitochondria, as mitochondria use metals (such as iron, copper, calcium, zinc) for the synthesis of cofactors of oxidation–reduction enzymes [447]. Deferiprone (DFP), an iron chelator used clinically in thalassemia, kidney disease, and Friedreich’s ataxia, has been identified to reduce the proliferation and migration of cancer cells [448, 449]. The underlying mechanisms are well documented to involve the suppression of mitochondrial metabolism and the respiration rate, as well as induction of ROS production [450, 451]. VLX600, a recently designed iron chelator, has been characterized as a mitochondrial OXPHOS inhibitor which exhibited outstanding antitumor ability ovarian and breast and colorectal cancers [452–454]. Intriguingly, VLX600 was reported to inhibit mitochondrial respiration and augment the efficacy of imatinib in gastrointestinal stromal tumors [455]. It has also been suggested to sensitize ovarian cancer cells to platinum agents and PARPis (two standard-of-care therapies) [456].

Another metal with important functions in cancer progression is copper. Tetrathiomolybdate, a copper-chelating drug used in the treatment of copper overload disorder, has also shown obvious antitumor effects. Besides reducing angiogenesis, it can impair mitochondrial respiration as well as ATP production mainly by inhibiting copper-dependent mitochondrial C IV activity [457, 458]. In recent decades, disulfiram, the alcohol-aversion drug which functions in a copper complex to treat alcohol abuse [459], has attracted considerable attention for its alone or synergetic anticancer activity [460–465]. It functions as a disulfiram-Cu^2+^ complex (DSF-Cu^+^/Cu^2+^) to induce mitochondrial fission and reduce MMP, thus suppressing tumors via a redox-related apoptosis process [466]. In addition, elesclomol exerts potent anticancer activity by inducing oxidative stress and apoptosis [467–471]. Mechanistically, elesclomol forms an elesclomol-Cu (II) complex by chelating copper (Cu) outside of cells, which rapidly transports copper into the mitochondria, thus inducing mitochondrial ROS accumulation [472, 473]. Other types of metal chelators, including zinc and calcium chelating agents, have also been recognized as effective antitumor agents [474, 475].

Additionally, there are other compounds that do not belong to the groups discussed above which could be repositioned for cancer therapy via mitochondrial-mediated mechanisms. For instance, besides the palliative effects, cannabinoids and their analogs have shown promise as antitumor agents to reduce proliferation, induce apoptosis and autophagy, inhibit invasion and angiogenesis, and improve chemosensitivity to anticancer drugs [476]. Unequivocally, cannabinoids have been demonstrated to disrupt mitochondria damage and trigger ROS production both in human primary tumors and those resistant to chemotherapeutic drugs [477–479]. Furthermore, many commonly used chemotherapeutic drugs have been proven to interfere with mitochondria to promote anticancer effects, including, but not limited to, cisplatin [480, 481], doxorubicin [482, 483], sorafenib [484], and tamoxifen [485]. This broad variety of agents provide a plethora of options for tumor therapy by targeting mitochondria. We believe that targeted delivery of these drugs to mitochondria could benefit cancer treatment and overcome drug resistance.

In summary, repurposing non-oncology drugs is considered as an effective strategy to alleviate the current lack of mitochondria-targeting drugs. It holds the potential to develop effective agents in a short time period with lower development costs. However, it is not trivial to successfully apply suitable non-oncology drugs as anticancer therapeutics. Assessment of their effectiveness and understanding the underlying mechanistic in preclinical models are critical.

### Mitochondria-targeted drug delivery system and multifunctional strategy

In recent years, organelle-specific delivery of bioactive molecules has been widely utilized for cancer treatment to achieve high selectivity, maximum therapeutic effects, minimum side effects, and minor resistance [486–490]. Mitochondria-targeting therapeutic strategies can directly affect the mitochondrial membrane or matrix, mitochondrial metabolism, and the mitochondrial apoptosis or regulatory signaling pathways [306, 491–494]. Researchers have developed or identified a number of mitochondria-targeted drug delivery systems (MTDDSs), with most of them currently transporting chemotherapeutics into the mitochondria based on the high membrane potential across the inner mitochondrial membrane or the mitochondrial protein import machinery [495–498]. The following section will provide insights into the application of novel mitochondria-targeting strategies for cancer therapy (Table 4).

**Table 4:** Representative mitochondria-targeting therapeutic regimens

Mitochondria-targeted machinery	Mitochondria-targeted delivery method	Therapeutic regimens	Molecular mechanisms	Effects on cancer	Ref
Mitochondrial protein import machinery	Mitochondria-targeting signal peptides	M-ChiP	ROS production	Necrosis, enhanced therapeutic effect with reduced side effect	[580]
		AuNR@MSN-ICG-β-CD/Ada-RLA/CS (DMA)-PEG	ROS production; local hyperthermia	Enhanced antitumor effect with minimal side effect	[581]
		p53-BakMTS (or BaxMTS) and DBD-BakMTS (or BaxMTS)	Activation of MOMP and caspase-9	Apoptosis	[507]
		DOX/CEL-MTS-R8H3	ROS production; inhibition of P-gp efflux activity	Overcoming drug resistance in breast cancer	[509]
	Protein nanoparticle	GST-MT-3(Co^2+^) NPs	ROS production; reduction of MMPs	Suppressing tumors and prolonging survival	[508]
Cell-penetrating peptide-based	Cell-penetrating peptide	Pal-pHK-pKV	Targeting the VDAC1-hexokinase-II complex	Amplifying lung cancer cell death	[524]
		DGLipo NPs	ROS production	Overcoming multidrug resistance	[582]
		TAT-PEG-DOPE system	/	Selectively killing tumor cells	[523]
		pHK-PAS	Cytochrome c release; disruption of the mitochondria-HKII association	Apoptosis	[525]
	MTP3	Mitochondrion-targeting prodrug (compound 17, doxorubicin-based prodrug)	Mitochondrial depolarization	Enhanced cytotoxicity against human tumor cells while negligible toxicity toward normal cells	[583]
Delocalized lipophilic cation (DLC)-based	Triphenylphosphonium (TPP)	TPP-LND-DOX NPs	Apoptosis	Conquering drug resistance	[243]
		TPP-PEG-L	Apoptosis	Enhancing paclitaxel-induced cytotoxicity and antitumor efficacy	[540]
		TPGS1000-TPP-Targeting paclitaxel liposomes	Apoptosis	Inhibiting drug-resistant lung cancer	[541]
		THMSNs@LMDI	Singlet oxygen generation	Sensitizing A549/MCF-7 cells to doxorubicin	[578]
		Fe3O4@Dex/TPP/PpIX/ss-mPEG	Fenton reaction	Improving antitumor therapeutic efficacy	[574]
		TPP-PF127-HA	Cytochrome c release; activation of caspase-3 and caspase-9	Eradicating drug-resistant of lung cancer	[497]
		Targeted Sunitinib Liposomes and Targeted Vinorelbine Liposomes	Activation of caspase-9 and caspase-3	Treating invasive breast cancer	[584]
	Dequalinium (DQA)	DQA-PEG2000-DSPE	Apoptosis	Enhancing the anticancer efficacy against cisplatin-resistant A549 cells	[545]
		DQA and folate-loaded functional DOX nanoparticles	Activation of caspase-9 and caspase-3 cascade	Overcoming multidrug-resistant cancers	[546]
	Metal complexes	Ir-photoacid generator (PAG)	^1^O_2_ generation	Killing cancer cells effectively even under hypoxic conditions	[585]
	Guanidinium	BZP, TPY, PPY, THPY	ROS elevation	Against cisplatin-resistant A549 cells	[586]
Synthetic secretion system in *E. coli*	T3SS	enT3SS	Cytotoxic activity	Eliminating tumors and reducing the mortality of tumor-bearing animals	[553]
Others	Coumarin	Bromocoumarin platinum 1	p53 apoptosis pathway	Overcoming cisplatin resistance	[556]
		ZIF-90@DDP	/	Overcoming platinum-resistant ovarian cancer	[557]
	Berberine (BBR)	Paclitaxel (PTX)-ss-BBR	ROS production; G2/M arrest	Enhancing the effect of CT in A549 cells	[587]
	d-(KLAKLAK)2	d-(KLAKLAK)2	Cytochrome c release	Enhancing the anticancer efficacy	[588]
	Ion-pair stabilized lipid matrix	Bio-nFeR	Apoptosis; Modulation of lipid metabolism	Enhanced bioavailability; Against multiple cancer stem cells	[589]
Enzymatic self-assembly	Enzymatic cleavage of branched peptides	Flag-(C16)2- CLRP	Inhibition of the mitochondrial protein synthesis; Cytochrome c release	Sensitizing cancer cells to cisplatin	[319, 590]

#### Mitochondrial protein import machinery-based targeting strategies

Except for a small number of mitochondria-encoded factors (e.g., key proteins in the ETC, rRNAs, tRNAs), the vast majority of proteins present in the mitochondria are encoded by the nucleus and translocated from the cytosol [499–503]. Transporting machinery protein complexes (e.g., TIM/TOM complex) recognize and transport these proteins from the cytoplasm to the mitochondria, where proteins with mitochondria-targeting signal peptides (MTSs) are escorted from the cytosol to mitochondrial outer membrane [504, 505]. MTSs always exhibit positive charge and easily form amphiphilic α-helices and thus have been successfully used for the selective and effective delivery of therapeutics to mitochondria for disease treatment, including cancer therapy [506]. In addition, MTSs conjugate to, and deliver, a variety of cargo molecules (e.g., proteins, nucleic acids). For instance, p53-BakMTS/p53-Bax were synthesized via fusing p53 or its DNA-binding domain (DBD) to MTSs from Bak or Bax by Matissek et al. This regiment is capable of targeting p53 to the mitochondria and executing mitochondria-mediated apoptosis in cancers [507]. Several mitochondria-targeting units take advantage of the IMM-embedded transporters. For example, a self-assembled protein nanoparticle named GST-MT-3(Co^2+^) NPs was prepared by Zhu et al., via covalently conjugating paclitaxel to GST-MT-3(Co^2+^), to specifically target mitochondria. Co^2+^ in the NPs depolarized the MMP and elevated ROS, which subsequently induced apoptosis to execute antitumor effects. Intriguingly, this nanoparticle exhibited a synergistic effect manifesting as 50-fold lower paclitaxel dosage which possessed a highly effective antitumor effect [508]. Similarly, a functional hybrid peptide (MTS-R8H3) was used to prepare a modified targeted liposome, DOX/CEL-MTS-R8H3 lipo, for codelivery of doxorubicin hydrochloride (DOX) and celecoxib (CEL) [509]. This liposome codelivery system exhibited remarkable treatment efficacy on killing DOX-resistant MCF-7 (MCF-7/ADR) cells, providing a promising strategy for overcoming drug resistance in breast cancer.

#### Cell-penetrating peptide-based mitochondria-targeting strategies

Cell-penetrating peptides (CPPs) are nontoxic, short, cationic, and/or amphipathic peptides able to directly cross the cellular membrane [510–512]. They serve as a popular and efficient vector for delivering a broad variety of cargoes, including oligonucleotides, proteins, and therapeutics [513–515]. Many efforts are being made to improve their cell specificity for selective uptake by tumor cells, permitting medical applications [516–518]. Modifying the CPPs according to microenvironment condition is a widely used strategy. Particularly, mitochondria-penetrating peptides (MPPs) have been developed to deliver a variety of antitumor cargoes into mitochondria, which can inhibit tumor growth in vivo and in vitro [519–522]. For example, Dox was intercalated into the Cyt c aptamer contained DNA duplex and subsequently loaded in the dendrigraftpoly-L-lysines (DGL) and combine to cyclopeptide RA-V contained pH-sensitive liposomal shells, for preparing a MPP-modified DGLipo NPs. This system could successively deliver both DOX and RA-V into lysosome and mitochondria of cancer cells, and achieved a spatiotemporally controlled release of them to monitor cytochrome c release and apoptotic process, leading to enhanced therapeutic outcomes in MDR tumors [462]. In addition, the TAT-PEG-DOPE system (methoxy (polyethylene glycol)-2000–1, 2-dioleoyl-sn-glycero-3-phosphoethanolamine (mPEG-DOPE) and transactivator of transcription (TAT) peptide conjugated PEG-DOPE) is an example, in which sulfonamide will lose charge and detach when it suffers a decrease in pH, so that exposed TAT can interact and take the drug-loaded micelles to selectively kill tumor cells [523]. Several other novel CPPs for targeting cancer cell mitochondria, including Pal-pHK-pKV, an engineered peptide performed with the N-terminus of the HK-II protein [524]; pHK-PAS, achieved by covalently coupling N-terminal 15 aa of HKII (pHK) to a short, penetration-accelerating sequence (PAS) [525]; MTP3, another engineered peptide synthesized via resin-based solid-phase peptide synthesis, are also serving as efficient tools to deliver exogenous therapeutics into mitochondria and representing promising strategy in cancer therapy.

#### Delocalized lipophilic cation (DLC)-based mitochondria-targeting strategies

It has been well demonstrated that the MMPs of tumor cells are usually higher than that of non-malignant cells [526–528]. The hydrophobic surface areas and delocalized positive charge of DLCs permit them to rapidly pass through membrane bilayers and accumulate in cancer cells because of the more negative MMPs in cancer cells [529–532]. This offers a selective drug delivery approach to deliver compounds to tumors with little toxicity to normal healthy cells.

While Rhodamin123 was the first DLC identified to markedly inhibit the growth of carcinoma cell lines and prolong the survival of tumor-bearing mice [533, 534], the triphenylphosphonium (TPP) cation is the most well-documented DLC that has been used for mitochondria targeting [346, 495, 535]. TPP^+^ cations were conjugated to a wide variety of synthesized residues and incorporated into the liposomal lipid bilayer to make drug delivery systems for mitochondria targeting and tumor suppression [536–539]. For example, Biswas et al. synthesized a polyethylene glycol-phosphatidylethanolamine (PEG-PE) and TPP^+^ group modified liposomes (TPP-PEG-L). TPP-PEG-L has been demonstrated to enhance paclitaxel-induced cytotoxicity and antitumor efficacy compared to plain liposomes (PL) from efficient mitochondria targeting [540]. In addition, a D-α-tocopheryl polyethylene glycol-1000 succinate-triphenylphosphine (TPGS1000-TPP) was incorporated onto the surface of paclitaxel liposomes to prepare TPGS1000-TPP conjugate. This regiment could selectively accumulate into the mitochondria and initiate caspase-9- and caspase-3-mediated apoptosis, thereby exhibiting significant anticancer efficacy in drug-resistant A549/cDDP xenograft and cells [541].

Dequalinium chloride (DQA) has been regarded as a new class of anti-carcinoma agents based on its selective localization and accumulation within the mitochondria of cancer cells [542–544]. For example, a dequalinium polyethylene glycol-distearoylphosphatidyl-dylethanolamine (DQA-PEG2000-DSPE) conjugate was synthesized to develop mitochondrial-targeted resveratrol liposomes to overcome drug resistance. This mitochondrial-targeted liposome is significantly accumulated in the mitochondria and induces apoptosis in both nonresistant and resistant cancer cells by dissipating MMPs. In addition, cotreating this liposome with vinorelbine liposomes remarkably enhanced the anticancer efficacy against cisplatin-resistant A549 cells [545]. Furthermore, functional nanoparticles based on DQA were developed for targeted delivery of classical cytotoxic anticancer drugs (such as doxorubicin) to tumor cells, which showed significant anticancer efficacy in a drug-resistant tumor model via triggering cytochrome c release and mitochondrial apoptosis [546].

#### Newly developed mitochondria-targeting units-based strategies

In recent years, numerous drug delivery systems, including liposomes, micelles, “smart” polymers, and hydrogels, have been developed for cancer therapy [547–552]. For instance, to achieve accurate delivery to mitochondria with high specificity and low size, a native genetic system encoded in *Salmonella* pathogenicity island-1 (SPI-1) was used by Lim et al. [553]. In their study, *E. coli* carrying synthetic T3SS and MTD on plasmids could eliminate tumors and reduce the mortality of tumor-bearing animals. Furthermore, another study developed a peri-mitochondrial enzymatic self-assembly system to deliver chloramphenicol (CLRP) to the mitochondria in cancer cells. Importantly, their results suggested that this new system could overcome cisplatin resistance by inhibiting the synthesis of mitochondrial proteins.

Modifying traditional drugs with newly developed mitochondria-targeting units also exhibited potential to reduce side effects and reverse drug resistance to some extent [554, 555]. For instance, Ma et al. designed bromocoumarin platinum 1 therapeutic (a coumarin-Pt (IV) prodrug) to simultaneously target mitochondria and nuclei [556]. This therapy allows simultaneous accumulation of high concentrations of Pt in both the nDNA and mtDNA, thus triggering apoptosis to overcome cisplatin resistance. Moreover, p53 activation promoted Pt–DNA-induced apoptosis in cancer cells, leading to obvious anticancer activity with this prodrug. In addition, Xing and co-workers synthesized a mitochondria-targeting zeolitic imidazole framework loaded with platinum (ZIF-90@ DDP) to kill cancer cells by promoting effective drug release under specific pH and ATP levels, thus providing a new strategy for reversing platinum resistance in ovarian cancer [557].

#### Multifunctional drug delivery strategies

At present, mitochondria-targeting photothermal therapy (PTT), photodynamic therapy (PDT), chemo-dynamic therapy (CDT), and related combinational therapies have attracted global attention due to their advantages of a wide therapeutic range, minimal toxicity, excellent safety profile, noninvasiveness, and low drug resistance [558–560]. PTT triggers thermal damage by conversing light energy into heat to kill cancer cells [561–563]. In recent years, a variety of photothermal materials, including inorganic nanomaterials (such as gold nanocages, gold nanorods, and other gold nanostructures), transition metal sulfide or oxide nanoparticles, have been developed to improve the energy conversion from near infrared (NIR) light [564, 565]. As such, PTT has shown remarkable achievements in the treatment of various tumors [566–569]. PDT is available for treating a broad variety of cancers through local ROS production only in the light-exposed region by utilizing photosensitizer (PS), light, and oxygen [570–573]. Recently, Fe_3_O_4_@Dex-TPP nanoparticles have been prepared by coprecipitation in TPP-grafted dextran (Dex-TPP) and Fe^2+^/Fe^3+^ and then incorporated with the photosensitizers of protoporphyrin IX (PpIX) and glutathione-responsive mPEG-ss-COOH to form a fenton reaction-assisted PDT, noted Fe_3_O_4_@Dex/TPP/PpIX/ss-mPEG nanoparticles [574]. This nanoparticle targets mitochondria by photoinduced internalization, leading to ROS generation and the fenton reaction-produced O_2_, thus significantly improving the therapeutic efficacy on tumor. In addition, Zeng et al. synthesized bifunctional nanoprobe (FA-NPs-DOX) by loading DOX to NaYF4:Yb/Tm-TiO2 inorganic photosensitizers for in vivo inorganic PDT [575]. In this study, folic acid (FA) targeting and NIR-triggered inorganic PDT accelerated the release of DOX and promoted the inhibition rate in drug-sensitive MCF-7 and resistant MCF-7/ADR cells.

In addition, other therapies, including CDT [576, 577], sonodynamic therapy (SDT), gas therapy, radiation therapy (RDT), alone or in combination with other treatments targeting mitochondria to inhibit tumors, are emerging, as described in a comprehensive review [491]. For instance, Shi et al. designed a mitochondria-targeted hollow mesoporous silica nanoparticles (THMSNs) loaded with L-menthol (LM) to carry DOX and NIR dye indocyanine green (ICG), named THMSNs@LMDI [578]. Under NIR irradiation, this system simultaneously produces photodynamic and photothermal therapy effect via DOX release and apoptosis activation, thereby sensitizing A549/MCF-7 cells to DOX. Intriguingly, a specific targeting of mitochondria and imaging-guided chemo-photothermal therapy against cisplatin resistance was proposed by Yang and colleagues [579]. In this work, Pt (IV)-NPs, a nanoparticle precisely assembled by biotin-labeled Pt (IV) prodrug derivative and cyclodextrin-functionalized IR780, integrated with targeting units, imaging moieties into a single regiment to overcome and even completely eliminate cisplatin resistance A549R tumors, thus providing a beneficial precise therapeutic. Undoubtedly, combination therapies achieve synergistic effect of anticancer and hold more beneficial for future clinical translation.

In summary, the development of mitochondria-targeting units and combinational strategies for cancer therapy has achieved precise treatment at lower drug doses (Table 4), offering excellent prospects for improving the therapeutic effect and overcoming drug resistance.

### Therapeutic applications of mitochondrial transplantation

The transplantation of mitochondria from healthy cells to abnormal cells has emerged as a novel and attractive therapeutic strategy to treat diseases caused by mitochondria damage or dysfunction [591–595]. While intercellular mitochondrial transfer functions as essential stress-adaptive mechanism to endow cancer cells with resistance to chemotherapy [175, 176], mitochondrial transplantation (mtTP) has been used in preclinical and clinical studies to restore mitochondrial function for cancer therapy and eliminate drug resistance [596–599]. For example, Chang et al. transferred mitochondria into breast cancer cell lines [321]. The results suggested that mitochondria transplantation-induced cell apoptosis inhibited cell growth and decreased oxidative stress, thereby increasing the susceptibility of both MCF-7 and MDA-MB-231 breast cancer cells to doxorubicin and paclitaxel. In addition, intercellular endocytosis (e.g., mitochondria internalization) was suggested to enhance the TCA cycle and aerobic respiration, attenuate glycolysis, and reactivate the mitochondrial apoptotic pathway, thereby inhibiting malignant proliferation and enhancing the radiosensitivity of gliomas in vitro and in vivo [600].

Overall, mtTP appears to be a very promising therapeutic option to fine-tune mitochondria function in cancer cells so that drug resistance might be overcome. However, research on mitochondrial transplantation for cancer treatment is still in its infancy. Further investigations including preclinical and clinical studies are required to determine if it is effective in sensitizing cancer cells to radio- or chemotherapy. Additionally, various technical and ethical issues need to be addressed before its actual clinical application.

## Conclusions and perspectives

**Fig. 4:**
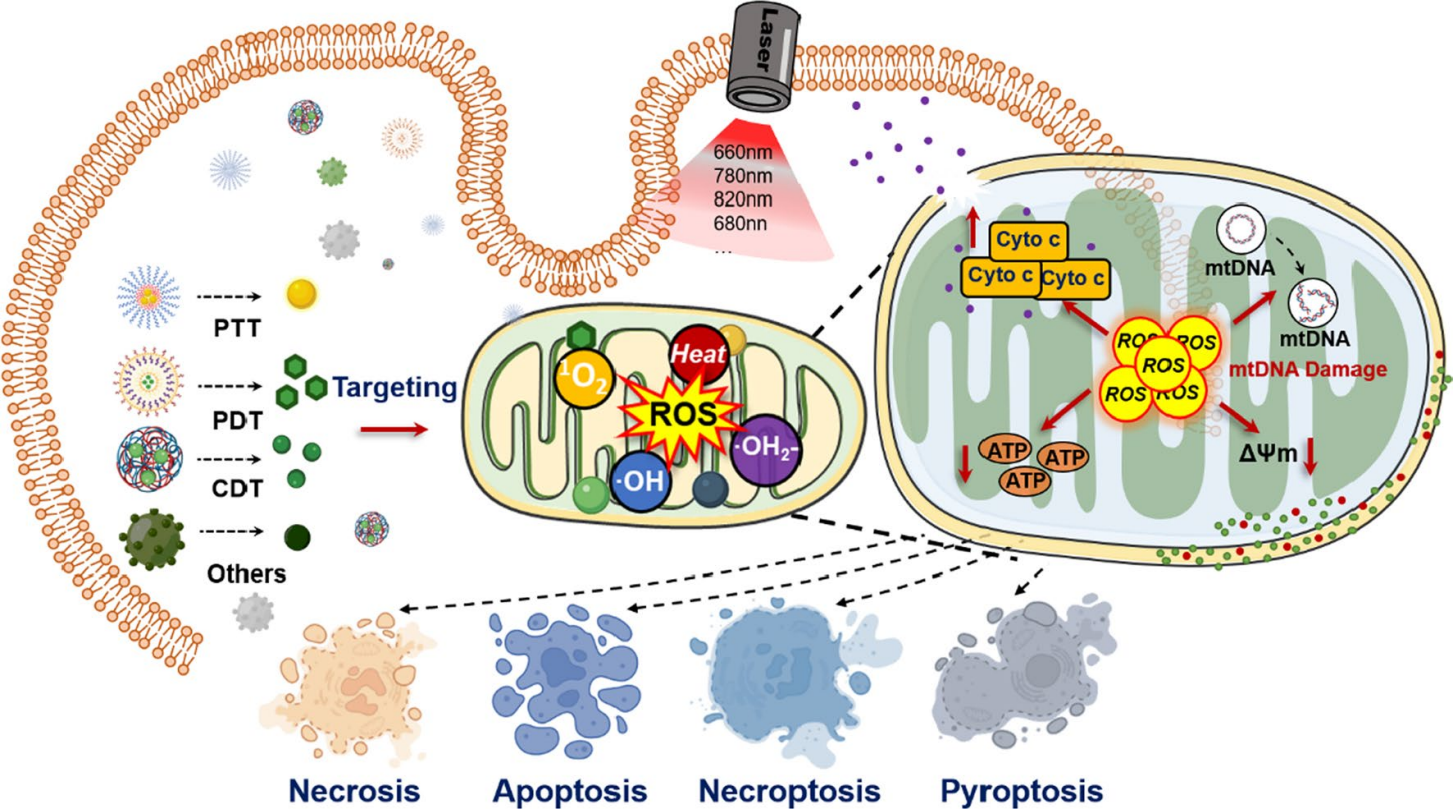
Schematic illustration of the mitochondria-targeting strategies and their anticancer effect. Integrated therapeutics include, but are not limited to, PTT, PDT, and CDT. Their function requires the rational design, functionalization, and application of diverse mitochondria-targeting units, such as organic phosphine/sulfur salts, QA salts, transition metal complexes, and MTPs. The generation of superoxide (**·**O_2_^−^), singlet oxygen (^1^O_2_), **·**OH or heat results in mitochondrial damage, thus inhibiting energy supply and triggering cancer cells death

Mitochondria are crucial players in cancer cell survival, as they are the bioenergetic and biosynthetic hub that coordinates cellular respiration, FAO, the TCA cycle, ETC, Ca^2+^ signaling, and redox homeostasis. Cancer drug resistance, as an adaptive strategy employed by cancer cells to survive stress conditions, is inevitably associated with mitochondrial-related pathways [62, 82, 601]. In fact, emerging evidence strongly indicates that resistant tumor cells exhibit high mitochondrial respiration and OXPHOS status [602, 603]. Therefore, targeting mitochondria represents a promising cancer treatment avenue and chemoresistance overcoming strategy. In this review, we have elaborated on the mechanisms of mitochondrial dynamics in number, structure, and location to maintaining mitochondrial function to endow cancer cells with metabolic flexibility for adapting to stress conditions, with an emphasis on their regulatory role in drug resistance. We have also summarized recent advances that focus on developing therapeutics that specifically target the mitochondria for cancer therapy. Notably, two representative compounds, metformin and CPI-613, have been taken on to phase III clinical trials (Table 2). Lastly, we have highlighted the repurposing of “old” drugs for mitochondria targeting in tumor therapy with the potential to effectively kill tumors. The development of mitochondria-targeting approaches will undisputedly boost the precision of cancer treatment at lower drug doses (Fig. 4, Table 5).

**Table 5:** Overview of mitochondria-targeting strategies for cancer treatment

Mitochondria-targeting strategies	Description	Compositions	Characteristics	Refs.
Conventional chemotherapy	A promising strategy that directly acts on mitochondria to produce toxic substances to cells and induces cancer cell death by endogenous chemical energy without the stimulation of external light sources	CT drugs (Betulinic Acid, Resveratrol, Ditercalinium Chloride, Benzo-α-pyrone (coumarin), α-TOS, Organic Arsenicals)	Easy to penetrate mitochondrial membrane and target mitochondria due to high lipid solubility; high effectiveness of tumor treatment	[556, 604, 605]
Nanoplatform loaded with CT/RT drugs	CT/RT drugs are modified by mitochondria-targeting units or designed as nanoplatforms for cancer treatment	CT/RT drugs (Lonidamine, Paclitaxel, Doxorubicin, Cisplatin); mitochondria-targeting units	High effectiveness of tumor treatment	[557, 587, 606]
CDT	A burgeoning therapy through undergoing a fenton reaction or a fenton-like reaction, which reacts with excessive intracellular hydrogen peroxide (H_2_O_2_) in tumor tissues to generate hydroxyl radicals (·OH)	CT drugs; mitochondria-targeting units	Low invasiveness; consumption of endogenous H_2_O_2_ without external energy; little normal tissue toxicity	[576, 577]
PTT	Killing cancer cells with thermal damage (conversion of light energy into heat) utilizing an external light source (usually near-infrared (NIR) light) and photothermal agent as heat-generating source; PTT has strong absorption characteristics for NIR	Photothermal materials, external light source; mitochondria-targeting unit	Deep penetration and minimal damage to surrounding healthy tissue; noninvasiveness; Minimal side effects; temporal and spatial selectivity	[567, 607, 608]
PDT	A locally targeted therapy utilizing a photosensitizer (PS), light, and oxygen to selectively kill tumors	PS, light, oxygen, serval lipophilic, and cationic groups	Accurate controllability; minimal drug resistance	[562, 570, 572]
RT-RDT	Stimulating PS to produce ^1^O_2_ to kill tumors under ionizing radiation	PS, ^1^O_2_, and mitochondria-targeting unit	Reach deeper tissues; low dosage possessing effective therapeutic effect	[609, 610]
SDT	To kill cancer cells by stimulating exogenous (ultrasound) to activate SDT agents for producing ROS, cavitation, air bubbles, and hyperthermia	Ultrasound, SDT agents, ROS, cavitation, air bubbles, and hyperthermia	Depth of tumor tissues can be realized by ultrasound; Achievement of noninvasive treatment; high precision of target lesion zones	[611]
Gene therapy	Replacement of defective genes by delivering wild-type ones into the host cell, or silencing a dominant mutant allele that is pathogenic to address mitochondrial diseases,	Therapeutic cargoes; delivery system	Precision treatment	[612–614]
Gas therapy	Using gaseous molecules to combat cancer	Gaseous molecules such as nitric oxide, CO, hydrogen sulfide, and hydrogen; mitochondria-targeting unit	Noninvasive in situ treatment with no depth limit	[615, 616]
Combination therapy	Combination of CT and PTT, CT and CDT, PDT and PTT, PDT and CDT; PDT and CT; PDT and Immunotherapy	/	Achieve synergistic effect of anticancer; minimize multidrug resistance; reduced pain in patients	[579, 617–620]

It is worth noting that further investigations are urgently needed to handle several key mitochondrial-related questions for their successful application in clinic cancer treatment. First, it will be pivotal to identify additional molecular mechanisms that cause the high OXPHOS status of cancer cells. It is also important to explore the roles and mechanisms of metabolic advantages in maintaining this high OXPHOS activity and how they modulate resistance to targeted or chemotherapies, as mitochondria are the hub of many metabolic pathways. Second, the roles of mitochondrial reshaping, rebuilding, and recycling are largely in a context-dependent manner, which remain vastly unexplored. Further study focusing on developing rational targeted approaches to modulate adaptive response will definitely require the possibility to accurately map dynamic processes and monitor bioenergetic and metabolic changes over a considerable time period [621]. Theoretically, drug repurposing and systematic screening approaches as well as advanced bioinformatics could replenish the inventory of antitumor drugs and break one of the current bottlenecks of drug development. However, it is important to decipher their mechanism of action and identify patients who would benefit from treatment with these compounds. In addition, more preclinical studies and clinical trials must be completed before such interventions become common practice in cancer therapy.

Modification of traditional therapeutics with mitochondria-targeting units has potential for reducing drug resistance and adverse side effects. Many of these strategies have been applied as preclinical or clinical antitumor therapies. However, safety evaluation based on biocompatibilities, release, accumulation, and metabolism is a prerequisite for their application. Indeed, limitations in the materials, such as toxicities and poor drug loadings, have restricted the further application of multifunctional nanodrugs. Future pharmaceutical research should focus on addressing the aspects mentioned above while exploring new materials. Notably, these therapeutics need to overcome both physiological and biological barriers before localizing to their target sites to take effect. What happens in these processes will affect the release of drugs and affect their antitumor efficacy. Therefore, it is important to endow the delivery system with some specific related functions. In addition to structural reformation, future research needs to investigate the mechanisms of exerting treatment, especially at the molecular level.

**Fig. 5:**
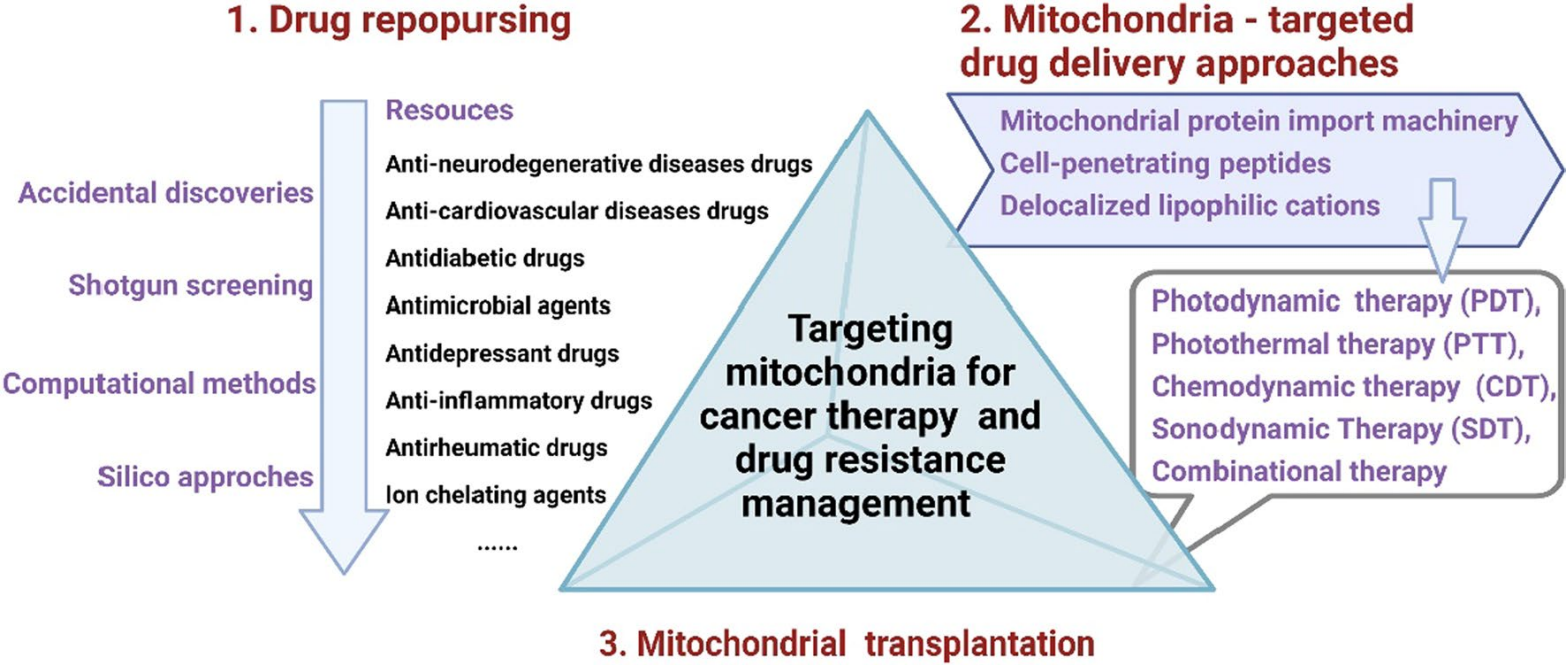
Rational design for targeting mitochondria in cancer therapy. Drug repurposing, mitochondrial-targeted nanomedicines, and mitochondrial transplantation represent opportunity to offer promising strategies for targeting mitochondria to overcome cancer drug resistance. The mitochondrial inhibitors may be used in combination with chemotherapy, radiotherapy, or even immunotherapy to provide new avenues for cancer therapeutic regimes

In the coming years, we predict that advances in omics technology, PET imaging combined with cancer genomics, will help a timely elucidation of metabolic vulnerabilities and lead to the recognition of rational combinations of mitochondria-targeting inhibitors with standard treatments, which will hopefully bring new and more effective strategies for cancer therapy and drug resistance management (Fig. 5) supporting precision/personalized medicine.

## Data Availability

Not applicable.

## Abbreviations

2-DG: 2-Deoxyglucose
3-BP: 3-Bromopyruvate
5-fu: 5-Fluorouracil
α-TOS: Alpha-tocopheryl succinate
α-KG: α-Ketoglutarate
AOA: Aminooxyacetate
AML: Acute myeloid leukemia
α-KGDH: α-Ketoglutarate dehydrogenase
AIF: Apoptosis-inducing factor
AREs: Antioxidant response elements
AKT: RAC-alpha serine/threonine-protein kinase
BNIP3/NIX: Bcl2/adenovirus E1B 19 kDa interacting protein 3
BCL2L13: Bcl-2-like protein 13
BMSCs: Bone marrow stromal cells
Bz-423: 1:4-Benzodiazepine derivative
BITC: Benzyl isothiocyanate
CSCs: Cancer stem cells
CPPs: Cell-penetrating peptides
CDT: Chemo-dynamic therapy
CPT1/2: Carnitine palmitoyltransferase ½
CHF: Congestive heart failure
COX-1/2: Cyclooxygenases 1 and 2
CLRP: Chloramphenicol
DQA: Dequalinium chloride
Dox: Doxorubicin
DRP1: Dynein-related protein 1
DFP: Deferiprone
DCA: Dichloroacetate
DHODH: Dihydroorotate dehydrogenase
DIM: 3,30-Diindolylmethane
EGFR: Epidermal growth factor receptor
EVs: Extracellular vesicles
ETC: Electron transport chain
ER: Endoplasmic reticulum
ESCC: Esophageal squamous cell carcinoma
EF: Euphorbia formosana Hayata
FUNDC1: FUN14 domain-containing protein 1
FDX1: Ferredoxin 1
FIS1: Fission protein homologous protein 1
FAO: Fatty acid oxidation
FAs: Fatty acids
GCS: Glycine cleavage system
GLUT1: Glucose transporter 1
GLS: Glutaminase
GLUD1: Glutamate dehydrogenase 1
GOT2: Glutamate oxaloacetate transaminase 2
GPT2: Glutamate pyruvate transaminase 2
GSH: Glutathione
HK2: Hexokinase 2
HCC: Hepatocellular carcinoma
HDAC: Histone deacetylase
IMM: Inner mitochondrial membrane
IDH: Isocitrate dehydrogenase
LDH: Lactate dehydrogenase
MTHFD1: Methylenetetrahydrofolate dehydrogenase 1
MTHFD1 L: Methylenetetrahydrofolate dehydrogenase 1-like
MTHFD2: Methylenetetrahydrofolate dehydrogenase 2
MDR: Multiple drug-resistant
MTPs: Mitochondria-targeting peptides
MFN: Mitofusin
MCL1: Myeloid leukemia cell differentiation protein
MAM: Mitochondrial-associated endoplasmic reticulum membrane
MCUC: Mitochondrial Ca^2+^ uniporter complex
MSCs: Marrow stromal cells
MM: Multiple myeloma
MDPs: Mitochondrial-derived peptides
MCT1: Monocarboxylate transporter
MTDDSs: Mitochondria-targeted drug delivery systems
MTS: Mitochondria-targeting signal peptides
MPPs: Mitochondria-penetrating peptides
MPP+: 1-Methyl-4-phenylpyridinium
NSAIDs: Nonsteroidal anti-inflammatory drugs
NPs: Nanoparticles
NSCLC: Non-small cell lung cancer
NIR: Near infrared
OXPHOS: Oxidative phosphorylation
OMM: Outer mitochondrial membrane
OPA1: Optic atrophy 1
PFK: Phosphofructokinase
PEG-PE: Polyethylene glycol-phosphatidylethanolamine
PKM2: Pyruvate kinase isozymes M2
PPP: Pentose phosphate pathway
PGC1α: Peroxisome proliferator-activated receptorγcoactivator-1
PL: Plain liposomes
PI3K: Phosphatidylinositol 3-kinase
PDT: Photodynamic therapy
PTT: Photothermal therapy
PDH: Pyruvate dehydrogenase
PTSD: Posttraumatic stress disorder
PDH: Pyruvate dehydrogenase
RDT: Radiation therapy
ROS: Reactive oxygen species
RIPK1: Receptor-interacting protein kinase 1
SHMT: Serine hydroxymethyltransferase
sORFs: Short open reading frames
SPI-1: Salmonella pathogenicity island-1
SDT: Sonodynamic therapy
TFAM: Transcription factor A, mitochondrial
TKI: Tyrosine kinase inhibitors
TCA cycle: Tricarboxylic acid cycle
TNTs: Tunneling nanotubes
TNBC: Triple-negative breast cancer
TPP: Methyltriphenylphosphonium
TAT: Transactivator of transcription
TrxR: Thioredoxin reductase
UPRmt: Unfolded protein stress response
VDAC1: Voltage-dependent anion-selective channel 1
VPA: Valproic acid
VDACs: Voltage-dependent anion-selective channel proteins
VK3: Vitamin K3

## References

[1] Holohan C, Van Schaeybroeck S, Longley DB, Johnston PG (2013). Cancer drug resistance: an evolving paradigm. Nat Rev Cancer.

[2] Phan TG, Croucher PI (2020). The dormant cancer cell life cycle. Nat Rev Cancer.

[3] Hanker AB, Sudhan DR, Arteaga CL (2020). Overcoming endocrine resistance in breast cancer. Cancer Cell.

[4] Gottesman MM (2002). Mechanisms of cancer drug resistance. Annu Rev Med.

[5] Marine J-C, Dawson S-J, Dawson MA (2020). Non-genetic mechanisms of therapeutic resistance in cancer. Nat Rev Cancer.

[6] Braun TP, Eide CA, Druker BJ (2020). Response and resistance to BCR-ABL1-targeted therapies. Cancer Cell.

[7] Jiang J, Zhang L, Chen H, Lei Y, Zhang T, Wang Y, Jin P, Lan J, Zhou L, Huang Z (2020). Regorafenib induces lethal autophagy arrest by stabilizing PSAT1 in glioblastoma. Autophagy.

[8] Zhang Z, Qin S, Chen Y, Zhou L, Yang M, Tang Y, Zuo J, Zhang J, Mizokami A, Nice EC, Chen HN, Huang C, Wei X (2022). Inhibition of NPC1L1 disrupts adaptive responses of drug-tolerant persister cells to chemotherapy. EMBO Mol Med..

[9] Rohwer N, Cramer T (2011). Hypoxia-mediated drug resistance: novel insights on the functional interaction of HIFs and cell death pathways. Drug Resistance Updates.

[10] Boulos JC, Yousof Idres MR, Efferth T (2020). Investigation of cancer drug resistance mechanisms by phosphoproteomics. Pharmacol Res.

[11] Li B, Jiang J, Assaraf YG, Xiao H, Chen Z-S, Huang C (2020). Surmounting cancer drug resistance: new insights from the perspective of N-methyladenosine RNA modification. Drug Resistance updates.

[12] Jin P, Jiang J, Xie N, Zhou L, Huang Z, Zhang L, Qin S, Fu S, Peng L, Gao W (2019). MCT1 relieves osimertinib-induced CRC suppression by promoting autophagy through the LKB1/AMPK signaling. Cell Death Dis.

[13] Gong K, Guo G, Gerber DE, Gao B, Peyton M, Huang C, Minna JD, Hatanpaa KJ, Kernstine K, Cai L (2018). TNF-driven adaptive response mediates resistance to EGFR inhibition in lung cancer. J Clin Investig.

[14] Eritja N, Chen B-J, Rodríguez-Barrueco R, Santacana M, Gatius S, Vidal A, Martí MD, Ponce J, Bergadà L, Yeramian A (2017). Autophagy orchestrates adaptive responses to targeted therapy in endometrial cancer. Autophagy.

[15] Ghosh JC, Siegelin MD, Vaira V, Faversani A, Tavecchio M, Chae YC, Lisanti S, Rampini P, Giroda M, Caino MC, et al. Adaptive mitochondrial reprogramming and resistance to PI3K therapy. J Natl Cancer Inst. 2015;107. 10.1093/jnci/dju502 PMC4565533 25650317

[16] Yang L, Shi P, Zhao G, Xu J, Peng W, Zhang J, Zhang G, Wang X, Dong Z, Chen F (2020). Targeting cancer stem cell pathways for cancer therapy. Signal Transduct Target Ther.

[17] Payandeh Z, Pirpour Tazehkand A, Barati G, Pouremamali F, Kahroba H, Baradaran B, Samadi N (2020). Role of Nrf2 and mitochondria in cancer stem cells; in carcinogenesis, tumor progression, and chemoresistance. Biochimie.

[18] Qin S, Li B, Ming H, Nice EC, Zou B, Huang C. Harnessing redox signaling to overcome therapeutic-resistant cancer dormancy. Biochimica et Biophysica Acta (BBA) - Rev Cancer. 2022;1877(4):188749. 10.1016/j.bbcan.2022.188749 35716972

[19] Li B, Huang Y, Ming H, Nice EC, Xuan R, Huang C (2021). Redox Control of the Dormant Cancer Cell Life Cycle. Cells.

[20] Smith RL, Soeters MR, Wüst RCI, Houtkooper RH (2018). Metabolic flexibility as an adaptation to energy resources and requirements in health and disease. Endocr Rev.

[21] Boumahdi S, de Sauvage FJ (2020). The great escape: tumour cell plasticity in resistance to targeted therapy. Nat Rev Drug Discovery.

[22] Cao Y (2019). Adipocyte and lipid metabolism in cancer drug resistance. J Clin Investig.

[23] Iwamoto H, Abe M, Yang Y, Cui D, Seki T, Nakamura M, Hosaka K, Lim S, Wu J, He X, et al. Cancer lipid metabolism confers antiangiogenic drug resistance. Cell Metabolism. 2018;28. 10.1016/j.cmet.2018.05.005 29861385

[24] Yoshida GJ (2015). Metabolic reprogramming: the emerging concept and associated therapeutic strategies. J Exp Clin Cancer Res: CR.

[25] Liu R, Lee J-H, Li J, Yu R, Tan L, Xia Y, Zheng Y, Bian X-L, Lorenzi PL, Chen Q, et al. Choline kinase alpha 2 acts as a protein kinase to promote lipolysis of lipid droplets. Mol Cell. 2021;81. 10.1016/j.molcel.2021.05.005 34077757

[26] Liu R, Li J, Shao J, Lee J-H, Qiu X, Xiao Y, Zhang B, Hao Y, Li M, Chen Q. Innate immune response orchestrates phosphoribosyl pyrophosphate synthetases to support DNA repair. Cell Metab. 2021;33. 10.1016/j.cmet.2021.07.009 34343500

[27] Ward PS, Thompson CB (2012). Metabolic reprogramming: a cancer hallmark even warburg did not anticipate. Cancer Cell.

[28] Kreuzaler P, Panina Y, Segal J, Yuneva M. Adapt and conquer: metabolic flexibility in cancer growth, invasion and evasion. Mol Metab. 2020;33. 10.1016/j.molmet.2019.08.021 PMC7056924 31668988

[29] Lunt SY, Vander Heiden MG (2011). Aerobic glycolysis: meeting the metabolic requirements of cell proliferation. Annu Rev Cell Dev Biol.

[30] Luengo A, Li Z, Gui DY, Sullivan LB, Zagorulya M, Do BT, Ferreira R, Naamati A, Ali A, Lewis CA, et al. Increased demand for NAD relative to ATP drives aerobic glycolysis. Mol Cell. 2021;81. 10.1016/j.molcel.2020.12.012 PMC8315838 33382985

[31] Liu J, Zhang C, Hu W, Feng Z (2019). Tumor suppressor p53 and metabolism. J Mol Cell Biol.

[32] Hoxhaj G, Manning BD (2020). The PI3K-AKT network at the interface of oncogenic signalling and cancer metabolism. Nat Rev Cancer.

[33] Papagiannakopoulos T, Bauer MR, Davidson SM, Heimann M, Subbaraj L, Bhutkar A, Bartlebaugh J, Vander Heiden MG, Jacks T (2016). Circadian rhythm disruption promotes lung tumorigenesis. Cell Metab.

[34] Garcia D, Shaw RJ (2017). AMPK: mechanisms of cellular energy sensing and restoration of metabolic balance. Mol Cell.

[35] Lin S-C, Hardie DG (2018). AMPK: sensing glucose as well as cellular energy status. Cell Metab.

[36] Labuschagne CF, Zani F, Vousden KH (2018). Control of metabolism by p53: cancer and beyond. Biochim Biophys Acta.

[37] Gomes AS, Ramos H, Soares J, Saraiva L (2018). p53 and glucose metabolism: an orchestra to be directed in cancer therapy. Pharmacol Res.

[38] Boroughs LK, DeBerardinis RJ (2015). Metabolic pathways promoting cancer cell survival and growth. Nat Cell Biol.

[39] Zhang Y, Yu G, Chu H, Wang X, Xiong L, Cai G, Liu R, Gao H, Tao B, Li W, et al. Macrophage-associated PGK1 phosphorylation promotes aerobic glycolysis and tumorigenesis. Mol Cell. 2018;71. 10.1016/j.molcel.2018.06.023 30029001

[40] Park MK, Zhang L, Min K-W, Cho J-H, Yeh C-C, Moon H, Hormaechea-Agulla D, Mun H, Ko S, Lee JW, et al. NEAT1 is essential for metabolic changes that promote breast cancer growth and metastasis. Cell Metab. 2021;33. 10.1016/j.cmet.2021.11.011 PMC8813003 34879239

[41] Lin J, Xia L, Liang J, Han Y, Wang H, Oyang L, Tan S, Tian Y, Rao S, Chen X (2019). The roles of glucose metabolic reprogramming in chemo- and radio-resistance. J Exp Clin Cancer Res.

[42] Botzer LE, Maman S, Sagi-Assif O, Meshel T, Nevo I, Yron I, Witz IP (2016). Hexokinase 2 is a determinant of neuroblastoma metastasis. Br J Cancer.

[43] Marcucci F, Rumio C (2021). Glycolysis-induced drug resistance in tumors-A response to danger signals?. Neoplasia.

[44] Wong T-L, Ng K-Y, Tan KV, Chan L-H, Zhou L, Che N, Hoo RLC, Lee TK, Richard S, Lo C-M (2020). CRAF methylation by PRMT6 regulates aerobic glycolysis-driven hepatocarcinogenesis via ERK-dependent PKM2 nuclear relocalization and activation. Hepatology (Baltimore, MD).

[45] Ma L, Zong X (2020). Metabolic symbiosis in chemoresistance: refocusing the role of aerobic glycolysis. Front Oncol.

[46] Fendt S-M, Frezza C, Erez A (2020). Targeting metabolic plasticity and flexibility dynamics for cancer therapy. Cancer Discov.

[47] Méndez-Lucas A, Lin W, Driscoll PC, Legrave N, Novellasdemunt L, Xie C, Charles M, Wilson Z, Jones NP, Rayport S (2020). Identifying strategies to target the metabolic flexibility of tumours. Nat Metab.

[48] Oshima N, Ishida R, Kishimoto S, Beebe K, Brender JR, Yamamoto K, Urban D, Rai G, Johnson MS, Benavides G, et al. Dynamic imaging of LDH inhibition in tumors reveals rapid in vivo metabolic rewiring and vulnerability to combination therapy. Cell Rep. 2020;30. 10.1016/j.celrep.2020.01.039 PMC7039685 32049011

[49] Stine ZE, Schug ZT, Salvino JM, Dang CV (2022). Targeting cancer metabolism in the era of precision oncology. Nat Rev Drug Discov.

[50] Wang K, Jiang J, Lei Y, Zhou S, Wei Y, Huang C (2019). Targeting metabolic-redox circuits for cancer therapy. Trends Biochem Sci.

[51] Jourdain AA, Begg BE, Mick E, Shah H, Calvo SE, Skinner OS, Sharma R, Blue SM, Yeo GW, Burge CB, et al. Loss of LUC7L2 and U1 snRNP subunits shifts energy metabolism from glycolysis to OXPHOS. Mol Cell. 2021;81. 10.1016/j.molcel.2021.02.033 PMC8314041 33852893

[52] Guitart AV, Panagopoulou TI, Villacreces A, Vukovic M, Sepulveda C, Allen L, Carter RN, van de Lagemaat LN, Morgan M, Giles P (2017). Fumarate hydratase is a critical metabolic regulator of hematopoietic stem cell functions. J Exp Med.

[53] Stuani L, Sabatier M, Saland E, Cognet G, Poupin N, Bosc C, Castelli FA, Gales L, Turtoi E, Montersino C, et al. Mitochondrial metabolism supports resistance to IDH mutant inhibitors in acute myeloid leukemia. J Exp Med. 2021;218. 10.1084/jem.20200924 PMC7995203 33760042

[54] Kim M, Gwak J, Hwang S, Yang S, Jeong SM (2019). Mitochondrial GPT2 plays a pivotal role in metabolic adaptation to the perturbation of mitochondrial glutamine metabolism. Oncogene.

[55] Eisner V, Picard M, Hajnóczky G (2018). Mitochondrial dynamics in adaptive and maladaptive cellular stress responses. Nat Cell Biol.

[56] Missiroli S, Perrone M, Genovese I, Pinton P, Giorgi C (2020). Cancer metabolism and mitochondria: finding novel mechanisms to fight tumours. EBioMedicine.

[57] Marchi S, Giorgi C, Galluzzi L, Pinton P (2020). Ca Fluxes and Cancer. Mol Cell.

[58] Song K-H, Kim J-H, Lee Y-H, Bae HC, Lee H-J, Woo SR, Oh SJ, Lee K-M, Yee C, Kim BW (2018). Mitochondrial reprogramming via ATP5H loss promotes multimodal cancer therapy resistance. J Clin Investig.

[59] Guerra F, Arbini AA, Moro L (2017). Mitochondria and cancer chemoresistance. Biochim Biophys Acta Bioenerg.

[60] Han Y, Kim B, Cho U, Park IS, Kim SI, Dhanasekaran DN, Tsang BK, Song YS (2019). Mitochondrial fission causes cisplatin resistance under hypoxic conditions via ROS in ovarian cancer cells. Oncogene.

[61] Xie L, Shi F, Li Y, Li W, Yu X, Zhao L, Zhou M, Hu J, Luo X, Tang M (2020). Drp1-dependent remodeling of mitochondrial morphology triggered by EBV-LMP1 increases cisplatin resistance. Signal Transduct Target Ther.

[62] Kuntz EM, Baquero P, Michie AM, Dunn K, Tardito S, Holyoake TL, Helgason GV, Gottlieb E (2017). Targeting mitochondrial oxidative phosphorylation eradicates therapy-resistant chronic myeloid leukemia stem cells. Nat Med.

[63] Liu J, Zhu C, Xu L, Wang D, Liu W, Zhang K, Zhang Z, Shi J (2020). Nanoenabled intracellular calcium bursting for safe and efficient reversal of drug resistance in tumor cells. Nano Lett.

[64] Yao J, Wang J, Xu Y, Guo Q, Sun Y, Liu J, Li S, Guo Y, Wei L. CDK9 inhibition blocks the initiation of PINK1-PRKN-mediated mitophagy by regulating the SIRT1-FOXO3-BNIP3 axis and enhances the therapeutic effects involving mitochondrial dysfunction in hepatocellular carcinoma. Autophagy. 2021. 10.1080/15548627.2021.2007027 PMC9450969 34890308

[65] Giacomello M, Pyakurel A, Glytsou C, Scorrano L (2020). The cell biology of mitochondrial membrane dynamics. Nat Rev Mol Cell Biol.

[66] Pernas L, Scorrano L (2016). Mito-morphosis: mitochondrial fusion, fission, and cristae remodeling as key mediators of cellular function. Annu Rev Physiol.

[67] Chan DC (2020). Mitochondrial dynamics and its involvement in disease. Annu Rev Pathol.

[68] Porporato PE, Filigheddu N, Pedro JMB, Kroemer G, Galluzzi L (2018). Mitochondrial metabolism and cancer. Cell Res.

[69] Burke PJ (2017). Mitochondria, bioenergetics and apoptosis in cancer. Trends Cancer.

[70] Garbincius JF, Elrod JW (2022). Mitochondrial calcium exchange in physiology and disease. Physiol Rev.

[71] Peoples JN, Saraf A, Ghazal N, Pham TT, Kwong JQ (2019). Mitochondrial dysfunction and oxidative stress in heart disease. Exp Mol Med.

[72] Willems PH, Rossignol R, Dieteren CE, Murphy MP, Koopman WJ (2015). Redox homeostasis and mitochondrial dynamics. Cell Metab.

[73] Bock FJ, Tait SWG (2020). Mitochondria as multifaceted regulators of cell death. Nat Rev Mol Cell Biol.

[74] Sabharwal SS, Schumacker PT (2014). Mitochondrial ROS in cancer: initiators, amplifiers or an Achilles' heel?. Nat Rev Cancer.

[75] Stacpoole PW. Therapeutic targeting of the pyruvate dehydrogenase complex/pyruvate dehydrogenase kinase (PDC/PDK) axis in cancer. J Natl Cancer Inst. 2017;109. 10.1093/jnci/djx071 29059435

[76] Morris JPt, Yashinskie JJ, Koche R, Chandwani R, Tian S, Chen CC, Baslan T, Marinkovic ZS, Sanchez-Rivera FJ, Leach SD, et al. alpha-Ketoglutarate links p53 to cell fate during tumour suppression. Nature. 2019;573:595–9. 10.1038/s41586-019-1577-5 PMC6830448 31534224

[77] Wang YP, Sharda A, Xu SN, van Gastel N, Man CH, Choi U, Leong WZ, Li X, Scadden DT (2021). Malic enzyme 2 connects the Krebs cycle intermediate fumarate to mitochondrial biogenesis. Cell Metab.

[78] Dalla Pozza E, Dando I, Pacchiana R, Liboi E, Scupoli MT, Donadelli M, Palmieri M (2020). Regulation of succinate dehydrogenase and role of succinate in cancer. Semin Cell Dev Biol.

[79] Izzo V, Bravo-San Pedro JM, Sica V, Kroemer G, Galluzzi L (2016). Mitochondrial permeability transition: new findings and persisting uncertainties. Trends Cell Biol.

[80] McGranahan N, Swanton C (2017). Clonal heterogeneity and tumor evolution: past, present, and the future. Cell.

[81] Ciplea AG, Richter KD (1988). The protective effect of Allium sativum and crataegus on isoprenaline-induced tissue necroses in rats. Arzneimittelforschung.

[82] Lee K-M, Giltnane JM, Balko JM, Schwarz LJ, Guerrero-Zotano AL, Hutchinson KE, Nixon MJ, Estrada MV, Sánchez V, Sanders ME, et al. MYC and MCL1 cooperatively promote chemotherapy-resistant breast cancer stem cells via regulation of mitochondrial oxidative phosphorylation. Cell Metab. 2017;26. 10.1016/j.cmet.2017.09.009 PMC5650077 28978427

[83] Wai T, Langer T (2016). Mitochondrial dynamics and metabolic regulation. Trends Endocrinol Metab.

[84] Desai R, East DA, Hardy L, Faccenda D, Rigon M, Crosby J, Alvarez MS, Singh A, Mainenti M, Hussey LK, et al. Mitochondria form contact sites with the nucleus to couple prosurvival retrograde response. Sci Adv. 2020;6. 10.1126/sciadv.abc9955 PMC11206220 33355129

[85] Drabik K, Malińska D, Piecyk K, Dębska-Vielhaber G, Vielhaber S, Duszyński J, Szczepanowska J. Effect of chronic stress present in fibroblasts derived from patients with a sporadic form of AD on mitochondrial function and mitochondrial turnover. Antioxidants (Basel, Switzerland). 2021;10. 10.3390/antiox10060938 PMC8229029 34200581

[86] LeBleu VS, O'Connell JT, Gonzalez Herrera KN, Wikman H, Pantel K, Haigis MC, de Carvalho FM, Damascena A, Domingos Chinen LT, Rocha RM, et al. PGC-1α mediates mitochondrial biogenesis and oxidative phosphorylation in cancer cells to promote metastasis. Nat Cell Biol. 2014;16. 10.1038/ncb3039 PMC4369153 25241037

[87] Dang CV, Le A, Gao P (2009). MYC-induced cancer cell energy metabolism and therapeutic opportunities. Clin Cancer Res.

[88] Li F, Wang Y, Zeller KI, Potter JJ, Wonsey DR, O'Donnell KA, Kim J-W, Yustein JT, Lee LA, Dang CV (2005). Myc stimulates nuclearly encoded mitochondrial genes and mitochondrial biogenesis. Mol Cell Biol.

[89] Yun CW, Han Y-S, Lee SH. PGC-1α Controls mitochondrial biogenesis in drug-resistant colorectal cancer cells by regulating endoplasmic reticulum stress. Int J Mol Sci. 2019;20. 10.3390/ijms20071707 PMC6480203 30959809

[90] Jiang J, Wang K, Chen Y, Chen H, Nice EC, Huang C (2017). Redox regulation in tumor cell epithelial-mesenchymal transition: molecular basis and therapeutic strategy. Signal Transduct Target Ther..

[91] Zhang G, Frederick DT, Wu L, Wei Z, Krepler C, Srinivasan S, Chae YC, Xu X, Choi H, Dimwamwa E (2016). Targeting mitochondrial biogenesis to overcome drug resistance to MAPK inhibitors. J Clin Invest.

[92] Xu R, Luo X, Ye X, Li H, Liu H, Du Q, Zhai Q (2021). SIRT1/PGC-1α/PPAR-γ correlate with hypoxia-induced chemoresistance in non-small cell lung cancer. Front Oncol.

[93] Gopal YNV, Rizos H, Chen G, Deng W, Frederick DT, Cooper ZA, Scolyer RA, Pupo G, Komurov K, Sehgal V (2014). Inhibition of mTORC1/2 overcomes resistance to MAPK pathway inhibitors mediated by PGC1α and oxidative phosphorylation in melanoma. Can Res.

[94] McCann E, O'Sullivan J, Marcone S (2021). Targeting cancer-cell mitochondria and metabolism to improve radiotherapy response. Translational oncology.

[95] Kondapalli C, Kazlauskaite A, Zhang N, Woodroof HI, Campbell DG, Gourlay R, Burchell L, Walden H, Macartney TJ, Deak M (2012). PINK1 is activated by mitochondrial membrane potential depolarization and stimulates Parkin E3 ligase activity by phosphorylating Serine 65. Open Biol.

[96] O'Flanagan CH, Morais VA, Wurst W, De Strooper B, O'Neill C (2015). The Parkinson's gene PINK1 regulates cell cycle progression and promotes cancer-associated phenotypes. Oncogene.

[97] Wu H, Wang Y, Li W, Chen H, Du L, Liu D, Wang X, Xu T, Liu L, Chen Q (2019). Deficiency of mitophagy receptor FUNDC1 impairs mitochondrial quality and aggravates dietary-induced obesity and metabolic syndrome. Autophagy.

[98] Yan C, Gong L, Chen L, Xu M, Abou-Hamdan H, Tang M, Désaubry L, Song Z (2020). PHB2 (prohibitin 2) promotes PINK1-PRKN/Parkin-dependent mitophagy by the PARL-PGAM5-PINK1 axis. Autophagy.

[99] Murakawa T, Yamaguchi O, Hashimoto A, Hikoso S, Takeda T, Oka T, Yasui H, Ueda H, Akazawa Y, Nakayama H (2015). Bcl-2-like protein 13 is a mammalian Atg32 homologue that mediates mitophagy and mitochondrial fragmentation. Nat Commun.

[100] Guan Y, Wang Y, Li B, Shen K, Li Q, Ni Y, Huang L (2021). Mitophagy in carcinogenesis, drug resistance and anticancer therapeutics. Cancer Cell Int.

[101] Hou H, Er P, Cheng J, Chen X, Ding X, Wang Y, Chen X, Yuan Z, Pang Q, Wang P (2017). High expression of FUNDC1 predicts poor prognostic outcomes and is a promising target to improve chemoradiotherapy effects in patients with cervical cancer. Cancer Med.

[102] Wu H, Wang T, Liu Y, Li X, Xu S, Wu C, Zou H, Cao M, Jin G, Lang J (2020). Mitophagy promotes sorafenib resistance through hypoxia-inducible ATAD3A dependent Axis. J Exp Clin Cancer Res: CR.

[103] Yamashita K, Miyata H, Makino T, Masuike Y, Furukawa H, Tanaka K, Miyazaki Y, Takahashi T, Kurokawa Y, Yamasaki M (2017). High expression of the mitophagy-related protein Pink1 is associated with a poor response to chemotherapy and a poor prognosis for patients treated with neoadjuvant chemotherapy for esophageal squamous cell carcinoma. Ann Surg Oncol.

[104] Villa E, Proïcs E, Rubio-Patiño C, Obba S, Zunino B, Bossowski JP, Rozier RM, Chiche J, Mondragón L, Riley JS (2017). Parkin-independent mitophagy controls chemotherapeutic response in cancer cells. Cell Rep.

[105] Su Y-C, Davuluri GVN, Chen C-H, Shiau D-C, Chen C-C, Chen C-L, Lin Y-S, Chang C-P (2016). Galectin-1-induced autophagy facilitates cisplatin resistance of hepatocellular carcinoma. PLoS ONE.

[106] Pietrocola F, Izzo V, Niso-Santano M, Vacchelli E, Galluzzi L, Maiuri MC, Kroemer G (2013). Regulation of autophagy by stress-responsive transcription factors. Semin Cancer Biol.

[107] Chen H, Vermulst M, Wang YE, Chomyn A, Prolla TA, McCaffery JM, Chan DC (2010). Mitochondrial fusion is required for mtDNA stability in skeletal muscle and tolerance of mtDNA mutations. Cell.

[108] Patten DA, Wong J, Khacho M, Soubannier V, Mailloux RJ, Pilon-Larose K, MacLaurin JG, Park DS, McBride HM, Trinkle-Mulcahy L (2014). OPA1-dependent cristae modulation is essential for cellular adaptation to metabolic demand. EMBO J.

[109] da Silva Rosa SC, Martens MD, Field JT, Nguyen L, Kereliuk SM, Hai Y, Chapman D, Diehl-Jones W, Aliani M, West AR (2021). BNIP3L/Nix-induced mitochondrial fission, mitophagy, and impaired myocyte glucose uptake are abrogated by PRKA/PKA phosphorylation. Autophagy.

[110] Liu H, Ho PW-L, Leung C-T, Pang SY-Y, Chang EES, Choi ZY-K, Kung MH-W, Ramsden DB, Ho S-L. Aberrant mitochondrial morphology and function associated with impaired mitophagy and DNM1L-MAPK/ERK signaling are found in aged mutant Parkinsonian LRRK2 mice. Autophagy. 2021;17:3196–220. 10.1080/15548627.2020.1850008 PMC8526027 33300446

[111] Bao D, Zhao J, Zhou X, Yang Q, Chen Y, Zhu J, Yuan P, Yang J, Qin T, Wan S (2019). Mitochondrial fission-induced mtDNA stress promotes tumor-associated macrophage infiltration and HCC progression. Oncogene.

[112] Osman C, Noriega TR, Okreglak V, Fung JC, Walter P (2015). Integrity of the yeast mitochondrial genome, but not its distribution and inheritance, relies on mitochondrial fission and fusion. Proc Natl Acad Sci USA.

[113] Yu Y, Peng X-D, Qian X-J, Zhang K-M, Huang X, Chen Y-H, Li Y-T, Feng G-K, Zhang H-L, Xu X-L (2021). Fis1 phosphorylation by Met promotes mitochondrial fission and hepatocellular carcinoma metastasis. Signal Transduct Target Ther.

[114] Rambold AS, Kostelecky B, Elia N, Lippincott-Schwartz J (2011). Tubular network formation protects mitochondria from autophagosomal degradation during nutrient starvation. Proc Natl Acad Sci USA.

[115] Hosseinzadeh A, Bahrampour Juybari K, Kamarul T, Sharifi AM (2019). Protective effects of atorvastatin on high glucose-induced oxidative stress and mitochondrial apoptotic signaling pathways in cultured chondrocytes. J Physiol Biochem.

[116] Nanda N (1990). Fine structure of the erythrocytic stages of Plasmodium vivax and the host cell alterations. Indian J Malariol.

[117] Han X-J, Yang Z-J, Jiang L-P, Wei Y-F, Liao M-F, Qian Y, Li Y, Huang X, Wang J-B, Xin H-B (2015). Mitochondrial dynamics regulates hypoxia-induced migration and antineoplastic activity of cisplatin in breast cancer cells. Int J Oncol.

[118] Chen X, Glytsou C, Zhou H, Narang S, Reyna DE, Lopez A, Sakellaropoulos T, Gong Y, Kloetgen A, Yap YS (2019). Targeting mitochondrial structure sensitizes acute myeloid leukemia to venetoclax treatment. Cancer Discov.

[119] Song J, Zhao W, Lu C, Shao X (2019). LATS2 overexpression attenuates the therapeutic resistance of liver cancer HepG2 cells to sorafenib-mediated death via inhibiting the AMPK-Mfn2 signaling pathway. Cancer Cell Int.

[120] Decker CW, Garcia J, Gatchalian K, Arceneaux D, Choi C, Han D, Hernandez JB (2020). Mitofusin-2 mediates doxorubicin sensitivity and acute resistance in Jurkat leukemia cells. Biochem Biophys Rep.

[121] Wang W-J, Lai H-Y, Zhang F, Shen W-J, Chu P-Y, Liang H-Y, Liu Y-B, Wang J-M. MCL1 participates in leptin-promoted mitochondrial fusion and contributes to drug resistance in gallbladder cancer. JCI Insight. 2021;6. 10.1172/jci.insight.135438 PMC8410075 34156978

[122] Li S, Wu Y, Ding Y, Yu M, Ai Z (2018). CerS6 regulates cisplatin resistance in oral squamous cell carcinoma by altering mitochondrial fission and autophagy. J Cell Physiol.

[123] Tomková V, Sandoval-Acuña C, Torrealba N, Truksa J (2019). Mitochondrial fragmentation, elevated mitochondrial superoxide and respiratory supercomplexes disassembly is connected with the tamoxifen-resistant phenotype of breast cancer cells. Free Radic Biol Med.

[124] Cai J, Wang J, Huang Y, Wu H, Xia T, Xiao J, Chen X, Li H, Qiu Y, Wang Y (2016). ERK/Drp1-dependent mitochondrial fission is involved in the MSC-induced drug resistance of T-cell acute lymphoblastic leukemia cells. Cell Death Dis.

[125] Huang Q, Zhan L, Cao H, Li J, Lyu Y, Guo X, Zhang J, Ji L, Ren T, An J, et al. Increased mitochondrial fission promotes autophagy and hepatocellular carcinoma cell survival through the ROS-modulated coordinated regulation of the NFKB and TP53 pathways. Autophagy. 2016;12. 10.1080/15548627.2016.1166318 PMC4922447 27124102

[126] Han Y, Cho U, Kim S, Park IS, Cho JH, Dhanasekaran DN, Song YS (2018). Tumour microenvironment on mitochondrial dynamics and chemoresistance in cancer. Free Radical Res.

[127] Csordás G, Weaver D, Hajnóczky G (2018). Endoplasmic reticulum-mitochondrial contactology: structure and signaling functions. Trends Cell Biol.

[128] Dia M, Gomez L, Thibault H, Tessier N, Leon C, Chouabe C, Ducreux S, Gallo-Bona N, Tubbs E, Bendridi N (2020). Reduced reticulum-mitochondria Ca transfer is an early and reversible trigger of mitochondrial dysfunctions in diabetic cardiomyopathy. Basic Res Cardiol.

[129] Horvath SE, Daum G (2013). Lipids of mitochondria. Prog Lipid Res.

[130] Acoba MG, Senoo N, Claypool SM. Phospholipid ebb and flow makes mitochondria go. J Cell Biol. 2020;219. 10.1083/jcb.202003131 PMC7401802 32614384

[131] Shadel GS, Horvath TL (2015). Mitochondrial ROS signaling in organismal homeostasis. Cell.

[132] Zhou B, Zhang J-Y, Liu X-S, Chen H-Z, Ai Y-L, Cheng K, Sun R-Y, Zhou D, Han J, Wu Q (2018). Tom20 senses iron-activated ROS signaling to promote melanoma cell pyroptosis. Cell Res.

[133] Lewis SC, Uchiyama LF, Nunnari J. ER-mitochondria contacts couple mtDNA synthesis with mitochondrial division in human cells. Science (New York, NY). 2016;353:aaf5549. 10.1126/science.aaf5549 PMC5554545 27418514

[134] Yang M, Li C, Sun L (2021). Mitochondria-associated membranes (MAMs): a novel therapeutic target for treating metabolic syndrome. Curr Med Chem.

[135] Büsselberg D, Florea A-M. Targeting intracellular calcium signaling ([Ca]) to overcome acquired multidrug resistance of cancer cells: a mini-overview. Cancers. 2017;9. 10.3390/cancers9050048 PMC5447958 28486397

[136] Genovese I, Carinci M, Modesti L, Aguiari G, Pinton P, Giorgi C. Mitochondria: insights into crucial features to overcome cancer chemoresistance. Int J Mol Sci. 2021;22. 10.3390/ijms22094770 PMC8124268 33946271

[137] Ren T, Wang J, Zhang H, Yuan P, Zhu J, Wu Y, Huang Q, Guo X, Zhang J, Ji L (2018). MCUR1-mediated mitochondrial calcium signaling facilitates cell survival of hepatocellular carcinoma via reactive oxygen species-dependent P53 degradation. Antioxid Redox Signal.

[138] Chen L, Sun Q, Zhou D, Song W, Yang Q, Ju B, Zhang L, Xie H, Zhou L, Hu Z (2017). HINT2 triggers mitochondrial Ca influx by regulating the mitochondrial Ca uniporter (MCU) complex and enhances gemcitabine apoptotic effect in pancreatic cancer. Cancer Lett.

[139] Hall DD, Wu Y, Domann FE, Spitz DR, Anderson ME (2014). Mitochondrial calcium uniporter activity is dispensable for MDA-MB-231 breast carcinoma cell survival. PLoS ONE.

[140] Marchi S, Lupini L, Patergnani S, Rimessi A, Missiroli S, Bonora M, Bononi A, Corrà F, Giorgi C, De Marchi E (2013). Downregulation of the mitochondrial calcium uniporter by cancer-related miR-25. Current biology : CB.

[141] Zeng F, Chen X, Cui W, Wen W, Lu F, Sun X, Ma D, Yuan Y, Li Z, Hou N (2018). RIPK1 Binds MCU to mediate induction of mitochondrial Ca uptake and promotes colorectal oncogenesis. Can Res.

[142] de Brito OM, Scorrano L (2008). Mitofusin 2 tethers endoplasmic reticulum to mitochondria. Nature.

[143] Friedman JR, Lackner LL, West M, DiBenedetto JR, Nunnari J, Voeltz GK (2011). ER tubules mark sites of mitochondrial division. Science (New York, NY).

[144] Rambold AS, Cohen S, Lippincott-Schwartz J (2015). Fatty acid trafficking in starved cells: regulation by lipid droplet lipolysis, autophagy, and mitochondrial fusion dynamics. Dev Cell.

[145] van Bergeijk P, Hoogenraad CC, Kapitein LC (2016). Right time, right place: probing the functions of organelle positioning. Trends Cell Biol.

[146] Lu L, Zhang J, Gan P, Wu L, Zhang X, Peng C, Zhou J, Chen X, Su J (2021). Novel functions of CD147 in the mitochondria exacerbates melanoma metastasis. Int J Biol Sci.

[147] Wang X, Chang X, He C, Fan Z, Yu Z, Yu B, Wu X, Hou J, Li J, Su L (2021). ATP5B promotes the metastasis and growth of gastric cancer by activating the FAK/AKT/MMP2 pathway. FASEB J.

[148] Caino MC, Ghosh JC, Chae YC, Vaira V, Rivadeneira DB, Faversani A, Rampini P, Kossenkov AV, Aird KM, Zhang R (2015). PI3K therapy reprograms mitochondrial trafficking to fuel tumor cell invasion. Proc Natl Acad Sci USA.

[149] Seo JH, Agarwal E, Bryant KG, Caino MC, Kim ET, Kossenkov AV, Tang H-Y, Languino LR, Gabrilovich DI, Cohen AR (2018). Syntaphilin ubiquitination regulates mitochondrial dynamics and tumor cell movements. Can Res.

[150] Caino MC, Seo JH, Wang Y, Rivadeneira DB, Gabrilovich DI, Kim ET, Weeraratna AT, Languino LR, Altieri DC (2017). Syntaphilin controls a mitochondrial rheostat for proliferation-motility decisions in cancer. J Clin Invest.

[151] Jung J-U, Ravi S, Lee DW, McFadden K, Kamradt ML, Toussaint LG, Sitcheran R (2016). NIK/MAP3K14 regulates mitochondrial dynamics and trafficking to promote cell invasion. Curr Biol.

[152] Yi M, Weaver D, Hajnóczky G (2004). Control of mitochondrial motility and distribution by the calcium signal: a homeostatic circuit. J Cell Biol.

[153] Schuler M-H, Lewandowska A, Caprio GD, Skillern W, Upadhyayula S, Kirchhausen T, Shaw JM, Cunniff B (2017). Miro1-mediated mitochondrial positioning shapes intracellular energy gradients required for cell migration. Mol Biol Cell.

[154] Nguyen TT, Oh SS, Weaver D, Lewandowska A, Maxfield D, Schuler M-H, Smith NK, Macfarlane J, Saunders G, Palmer CA (2014). Loss of Miro1-directed mitochondrial movement results in a novel murine model for neuron disease. Proc Natl Acad Sci USA.

[155] Alshaabi H, Shannon N, Gravelle R, Milczarek S, Messier T, Cunniff B (2021). Miro1-mediated mitochondrial positioning supports subcellular redox status. Redox Biol.

[156] Caino MC, Chae YC, Vaira V, Ferrero S, Nosotti M, Martin NM, Weeraratna A, O'Connell M, Jernigan D, Fatatis A (2013). Metabolic stress regulates cytoskeletal dynamics and metastasis of cancer cells. J Clin Investig.

[157] Desai SP, Bhatia SN, Toner M, Irimia D (2013). Mitochondrial localization and the persistent migration of epithelial cancer cells. Biophys J.

[158] Wanka H, Lutze P, Staar D, Albers A, Bäumgen I, Grunow B, Peters J (2020). Non-secretory renin reduces oxidative stress and increases cardiomyoblast survival during glucose and oxygen deprivation. Sci Rep.

[159] Onodera Y, Nam J-M, Horikawa M, Shirato H, Sabe H (2018). Arf6-driven cell invasion is intrinsically linked to TRAK1-mediated mitochondrial anterograde trafficking to avoid oxidative catastrophe. Nat Commun.

[160] Altieri DC (2017). Mitochondria on the move: emerging paradigms of organelle trafficking in tumour plasticity and metastasis. Br J Cancer.

[161] Dong L-F, Kovarova J, Bajzikova M, Bezawork-Geleta A, Svec D, Endaya B, Sachaphibulkij K, Coelho AR, Sebkova N, Ruzickova A, et al. Horizontal transfer of whole mitochondria restores tumorigenic potential in mitochondrial DNA-deficient cancer cells. eLife. 2017;6. 10.7554/eLife.22187 PMC5367896 28195532

[162] Levoux J, Prola A, Lafuste P, Gervais M, Chevallier N, Koumaiha Z, Kefi K, Braud L, Schmitt A, Yacia A, et al. Platelets facilitate the wound-healing capability of mesenchymal stem cells by mitochondrial transfer and metabolic reprogramming. Cell Metab. 2021;33. 10.1016/j.cmet.2021.02.003 33657394

[163] Liu D, Gao Y, Liu J, Huang Y, Yin J, Feng Y, Shi L, Meloni BP, Zhang C, Zheng M (2021). Intercellular mitochondrial transfer as a means of tissue revitalization. Signal Transduct Target Ther.

[164] Li H, Wang C, He T, Zhao T, Chen Y-Y, Shen Y-L, Zhang X, Wang L-L (2019). Mitochondrial transfer from bone marrow mesenchymal stem cells to motor neurons in spinal cord injury rats via gap junction. Theranostics.

[165] Islam MN, Das SR, Emin MT, Wei M, Sun L, Westphalen K, Rowlands DJ, Quadri SK, Bhattacharya S, Bhattacharya J (2012). Mitochondrial transfer from bone-marrow-derived stromal cells to pulmonary alveoli protects against acute lung injury. Nat Med.

[166] Dutra Silva J, Su Y, Calfee CS, Delucchi KL, Weiss D, McAuley DF, O'Kane C, Krasnodembskaya AD. Mesenchymal stromal cell extracellular vesicles rescue mitochondrial dysfunction and improve barrier integrity in clinically relevant models of ARDS. Eur Respir J. 2021;58. 10.1183/13993003.02978-2020 PMC8318599 33334945

[167] Jackson MV, Morrison TJ, Doherty DF, McAuley DF, Matthay MA, Kissenpfennig A, O'Kane CM, Krasnodembskaya AD (2016). Mitochondrial transfer via tunneling nanotubes is an important mechanism by which mesenchymal stem cells enhance macrophage phagocytosis in the in vitro and in vivo models of ARDS. Stem Cells (Dayton, Ohio).

[168] Nasoni MG, Carloni S, Canonico B, Burattini S, Cesarini E, Papa S, Pagliarini M, Ambrogini P, Balduini W, Luchetti F (2021). Melatonin reshapes the mitochondrial network and promotes intercellular mitochondrial transfer via tunneling nanotubes after ischemic-like injury in hippocampal HT22 cells. J Pineal Res.

[169] Griessinger E, Moschoi R, Biondani G, Peyron J-F (2017). Mitochondrial transfer in the leukemia microenvironment. Trends Cancer.

[170] Shanmughapriya S, Langford D, Natarajaseenivasan K (2020). Inter and Intracellular mitochondrial trafficking in health and disease. Ageing Res Rev.

[171] Tan AS, Baty JW, Dong L-F, Bezawork-Geleta A, Endaya B, Goodwin J, Bajzikova M, Kovarova J, Peterka M, Yan B (2015). Mitochondrial genome acquisition restores respiratory function and tumorigenic potential of cancer cells without mitochondrial DNA. Cell Metab.

[172] Pasquier J, Guerrouahen BS, Al Thawadi H, Ghiabi P, Maleki M, Abu-Kaoud N, Jacob A, Mirshahi M, Galas L, Rafii S (2013). Preferential transfer of mitochondria from endothelial to cancer cells through tunneling nanotubes modulates chemoresistance. J Transl Med.

[173] Spees JL, Olson SD, Whitney MJ, Prockop DJ (2006). Mitochondrial transfer between cells can rescue aerobic respiration. Proc Natl Acad Sci U S A.

[174] Moschoi R, Imbert V, Nebout M, Chiche J, Mary D, Prebet T, Saland E, Castellano R, Pouyet L, Collette Y (2016). Protective mitochondrial transfer from bone marrow stromal cells to acute myeloid leukemic cells during chemotherapy. Blood.

[175] Marlein CR, Piddock RE, Mistry JJ, Zaitseva L, Hellmich C, Horton RH, Zhou Z, Auger MJ, Bowles KM, Rushworth SA (2019). CD38-driven mitochondrial trafficking promotes bioenergetic plasticity in multiple myeloma. Cancer Res.

[176] Marlein CR, Zaitseva L, Piddock RE, Robinson SD, Edwards DR, Shafat MS, Zhou Z, Lawes M, Bowles KM, Rushworth SA (2017). NADPH oxidase-2 derived superoxide drives mitochondrial transfer from bone marrow stromal cells to leukemic blasts. Blood.

[177] Patheja P, Sahu K (2017). Macrophage conditioned medium induced cellular network formation in MCF-7 cells through enhanced tunneling nanotube formation and tunneling nanotube mediated release of viable cytoplasmic fragments. Exp Cell Res.

[178] Guaragnella N, Giannattasio S, Moro L (2014). Mitochondrial dysfunction in cancer chemoresistance. Biochem Pharmacol.

[179] Gustafsson CM, Falkenberg M, Larsson N-G (2016). Maintenance and expression of mammalian mitochondrial DNA. Annu Rev Biochem.

[180] Tigano M, Vargas DC, Tremblay-Belzile S, Fu Y, Sfeir A (2021). Nuclear sensing of breaks in mitochondrial DNA enhances immune surveillance. Nature.

[181] Rusecka J, Kaliszewska M, Bartnik E, Tońska K (2018). Nuclear genes involved in mitochondrial diseases caused by instability of mitochondrial DNA. J Appl Genet.

[182] Garcia-Roves PM, Osler ME, Holmström MH, Zierath JR (2008). Gain-of-function R225Q mutation in AMP-activated protein kinase gamma3 subunit increases mitochondrial biogenesis in glycolytic skeletal muscle. J Biol Chem.

[183] Egan DF, Shackelford DB, Mihaylova MM, Gelino S, Kohnz RA, Mair W, Vasquez DS, Joshi A, Gwinn DM, Taylor R (2011). Phosphorylation of ULK1 (hATG1) by AMP-activated protein kinase connects energy sensing to mitophagy. Science (New York, NY).

[184] Vaeth M, Maus M, Klein-Hessling S, Freinkman E, Yang J, Eckstein M, Cameron S, Turvey SE, Serfling E, Berberich-Siebelt F, et al. Store-operated Ca entry controls clonal expansion of T cells through metabolic reprogramming. Immunity. 2017;47. 10.1016/j.immuni.2017.09.003 PMC5683398 29030115

[185] Zhao P, Ming Q, Qiu J, Tian D, Liu J, Shen J, Liu Q-H, Yang X. Ethanolic extract of folium sennae mediates the glucose uptake of L6 cells by GLUT4 and Ca. molecules (Basel, Switzerland). 2018;23. 10.3390/molecules23112934 PMC6278344 30424024

[186] Song Z, Levin BE, Stevens W, Sladek CD (2014). Supraoptic oxytocin and vasopressin neurons function as glucose and metabolic sensors. Am J Physiol Regul Integr Comp Physiol.

[187] Zhang J, Wang X, Vikash V, Ye Q, Wu D, Liu Y, Dong W (2016). ROS and ROS-mediated cellular signaling. Oxid Med Cell Longev.

[188] Sankar N, deTombe PP, Mignery GA (2014). Calcineurin-NFATc regulates type 2 inositol 1,4,5-trisphosphate receptor (InsP3R2) expression during cardiac remodeling. J Biol Chem.

[189] Tan DQ, Suda T (2018). Reactive oxygen species and mitochondrial homeostasis as regulators of stem cell fate and function. Antioxid Redox Signal.

[190] Murakami S, Motohashi H (2015). Roles of Nrf2 in cell proliferation and differentiation. Free Radical Biol Med.

[191] Gambhir L, Sharma V, Kandwal P, Saxena S (2019). Perturbation in cellular redox homeostasis: decisive regulator of T cell mediated immune responses. Int Immunopharmacol.

[192] Gonzalez-Sanchez E, Marin JJG, Perez MJ (2014). The expression of genes involved in hepatocellular carcinoma chemoresistance is affected by mitochondrial genome depletion. Mol Pharm.

[193] Shen L, Zhou L, Xia M, Lin N, Ma J, Dong D, Sun L (2021). PGC1α regulates mitochondrial oxidative phosphorylation involved in cisplatin resistance in ovarian cancer cells via nucleo-mitochondrial transcriptional feedback. Exp Cell Res.

[194] Dogan SA, Pujol C, Maiti P, Kukat A, Wang S, Hermans S, Senft K, Wibom R, Rugarli EI, Trifunovic A (2014). Tissue-specific loss of DARS2 activates stress responses independently of respiratory chain deficiency in the heart. Cell Metab.

[195] Bahat A, Perlberg S, Melamed-Book N, Isaac S, Eden A, Lauria I, Langer T, Orly J (2015). Transcriptional activation of LON Gene by a new form of mitochondrial stress: a role for the nuclear respiratory factor 2 in StAR overload response (SOR). Mol Cell Endocrinol.

[196] Tan K, Fujimoto M, Takii R, Takaki E, Hayashida N, Nakai A (2015). Mitochondrial SSBP1 protects cells from proteotoxic stresses by potentiating stress-induced HSF1 transcriptional activity. Nat Commun.

[197] Tsvetkov P, Detappe A, Cai K, Keys HR, Brune Z, Ying W, Thiru P, Reidy M, Kugener G, Rossen J (2019). Mitochondrial metabolism promotes adaptation to proteotoxic stress. Nat Chem Biol.

[198] Benayoun BA, Lee C (2019). MOTS-c: a mitochondrial-encoded regulator of the nucleus. BioEssays.

[199] Lee C, Yen K, Cohen P (2013). Humanin: a harbinger of mitochondrial-derived peptides?. Trends Endocrinol Metab.

[200] Nashine S, Cohen P, Nesburn AB, Kuppermann BD, Kenney MC (2018). Characterizing the protective effects of SHLP2, a mitochondrial-derived peptide, in macular degeneration. Sci Rep.

[201] Mangalhara KC, Shadel GS (2018). A mitochondrial-derived peptide exercises the nuclear option. Cell Metab.

[202] Kim S-J, Xiao J, Wan J, Cohen P, Yen K (2017). Mitochondrially derived peptides as novel regulators of metabolism. J Physiol.

[203] Merry TL, Chan A, Woodhead JST, Reynolds JC, Kumagai H, Kim S-J, Lee C (2020). Mitochondrial-derived peptides in energy metabolism. Am J Physiol Endocrinol Metab.

[204] Sreekumar PG, Ishikawa K, Spee C, Mehta HH, Wan J, Yen K, Cohen P, Kannan R, Hinton DR (2016). The mitochondrial-derived peptide humanin protects RPE cells from oxidative stress, senescence, and mitochondrial dysfunction. Invest Ophthalmol Vis Sci.

[205] Cobb LJ, Lee C, Xiao J, Yen K, Wong RG, Nakamura HK, Mehta HH, Gao Q, Ashur C, Huffman DM (2016). Naturally occurring mitochondrial-derived peptides are age-dependent regulators of apoptosis, insulin sensitivity, and inflammatory markers. Aging.

[206] Kim KH, Son JM, Benayoun BA, Lee C. The mitochondrial-encoded peptide MOTS-c translocates to the nucleus to regulate nuclear gene expression in response to metabolic stress. Cell metabolism. 2018;28. 10.1016/j.cmet.2018.06.008 PMC6185997 29983246

[207] Moreno Ayala MA, Gottardo MF, Zuccato CF, Pidre ML, Nicola Candia AJ, Asad AS, Imsen M, Romanowski V, Creton A, Isla Larrain M (2020). Humanin promotes tumor progression in experimental triple negative breast cancer. Sci Rep.

[208] Dieli-Conwright CM, Sami N, Norris MK, Wan J, Kumagai H, Kim S-J, Cohen P (2021). Effect of aerobic and resistance exercise on the mitochondrial peptide MOTS-c in Hispanic and Non-Hispanic White breast cancer survivors. Sci Rep.

[209] Garcia-Mayea Y, Mir C, Masson F, Paciucci R, ME LL. Insights into new mechanisms and models of cancer stem cell multidrug resistance. Semin Cancer Biol. 2020;60:166–80. 10.1016/j.semcancer.2019.07.022 31369817

[210] Lee KM, Giltnane JM, Balko JM, Schwarz LJ, Guerrero-Zotano AL, Hutchinson KE, Nixon MJ, Estrada MV, Sanchez V, Sanders ME (2017). MYC and MCL1 cooperatively promote chemotherapy-resistant breast cancer stem cells via regulation of mitochondrial oxidative phosphorylation. Cell Metab.

[211] Kim B, Jung JW, Jung J, Han Y, Suh DH, Kim HS, Dhanasekaran DN, Song YS (2017). PGC1alpha induced by reactive oxygen species contributes to chemoresistance of ovarian cancer cells. Oncotarget.

[212] Farnie G, Sotgia F, Lisanti MP (2015). High mitochondrial mass identifies a sub-population of stem-like cancer cells that are chemo-resistant. Oncotarget.

[213] Cocetta V, Ragazzi E, Montopoli M. Mitochondrial Involvement in Cisplatin Resistance. International journal of molecular sciences. 2019;20. 10.3390/ijms20143384 PMC6678541 31295873

[214] Ma Y, Wang L, Jia R (2020). The role of mitochondrial dynamics in human cancers. Am J Cancer Res.

[215] Wang Y, Liu H-H, Cao Y-T, Zhang L-L, Huang F, Yi C (2020). The role of mitochondrial dynamics and mitophagy in carcinogenesis, metastasis and therapy. Front Cell Dev Biol.

[216] Kingnate C, Charoenkwan K, Kumfu S, Chattipakorn N, Chattipakorn SC (2018). Possible roles of mitochondrial dynamics and the effects of pharmacological interventions in chemoresistant ovarian cancer. EBioMedicine.

[217] Anderson NM, Mucka P, Kern JG, Feng H (2018). The emerging role and targetability of the TCA cycle in cancer metabolism. Protein Cell.

[218] Vasan K, Werner M, Chandel NS (2020). Mitochondrial metabolism as a target for cancer therapy. Cell Metab.

[219] Pesini A, Iglesias E, Garrido N, Bayona-Bafaluy MP, Montoya J, Ruiz-Pesini E (2014). OXPHOS, pyrimidine nucleotides, and Alzheimer's disease: a pharmacogenomics approach. J Alzheimer's Dis: JAD.

[220] Morgan JM, Duncan MC, Johnson KS, Diepold A, Lam H, Dupzyk AJ, Martin LR, Wong WR, Armitage JP, Linington RG, et al. Piericidin A1 blocks Ysc Type III secretion system needle assembly. MSphere. 2017;2. 10.1128/mSphere.00030-17 PMC5311113 28217742

[221] Panina SB, Pei J, Baran N, Konopleva M, Kirienko NV (2020). Utilizing synergistic potential of mitochondria-targeting drugs for leukemia therapy. Front Oncol.

[222] Preston S, Korhonen PK, Mouchiroud L, Cornaglia M, McGee SL, Young ND, Davis RA, Crawford S, Nowell C, Ansell BRE (2017). Deguelin exerts potent nematocidal activity the mitochondrial respiratory chain. FASEB J.

[223] Moreira PI, Custódio J, Moreno A, Oliveira CR, Santos MS (2006). Tamoxifen and estradiol interact with the flavin mononucleotide site of complex I leading to mitochondrial failure. J Biol Chem.

[224] Feng J, Wang X, Ye X, Ares I, Lopez-Torres B, Martínez M, Martínez-Larrañaga M-R, Wang X, Anadón A, Martínez M-A (2022). Mitochondria as an important target of metformin: the mechanism of action, toxic and side effects, and new therapeutic applications. Pharmacol Res.

[225] Wang H, Luo J, Tian W, Yan W, Ge S, Zhang Y, Sun W (2019). γ-Tocotrienol inhibits oxidative phosphorylation and triggers apoptosis by inhibiting mitochondrial complex I subunit NDUFB8 and complex II subunit SDHB. Toxicology.

[226] Prochazka L, Koudelka S, Dong L-F, Stursa J, Goodwin J, Neca J, Slavik J, Ciganek M, Masek J, Kluckova K (2013). Mitochondrial targeting overcomes ABCA1-dependent resistance of lung carcinoma to α-tocopheryl succinate. Apoptosis.

[227] Dong L-F, Jameson VJA, Tilly D, Prochazka L, Rohlena J, Valis K, Truksa J, Zobalova R, Mahdavian E, Kluckova K (2011). Mitochondrial targeting of α-tocopheryl succinate enhances its pro-apoptotic efficacy: a new paradigm for effective cancer therapy. Free Radical Biol Med.

[228] Dörrie J, Gerauer H, Wachter Y, Zunino SJ (2001). Resveratrol induces extensive apoptosis by depolarizing mitochondrial membranes and activating caspase-9 in acute lymphoblastic leukemia cells. Can Res.

[229] Salata GC, Malagó ID, Carvalho Dartora VFM, Marçal Pessoa AF, Fantini MCdA, Costa SKP, Machado-Neto JA, Lopes LB. Microemulsion for prolonged release of fenretinide in the mammary tissue and prevention of breast cancer development. Mol Pharm. 2021;18:3401–17. 10.1021/acs.molpharmaceut.1c00319 34482696

[230] Sundberg TB, Ney GM, Subramanian C, Opipari AW, Glick GD (2006). The immunomodulatory benzodiazepine Bz-423 inhibits B-cell proliferation by targeting c-myc protein for rapid and specific degradation. Cancer Res.

[231] Alam MM, Sohoni S, Kalainayakan SP, Garrossian M, Zhang L (2016). Cyclopamine tartrate, an inhibitor of Hedgehog signaling, strongly interferes with mitochondrial function and suppresses aerobic respiration in lung cancer cells. BMC Cancer.

[232] Sohoni S, Ghosh P, Wang T, Kalainayakan SP, Vidal C, Dey S, Konduri PC, Zhang L (2019). Elevated heme synthesis and uptake underpin intensified oxidative metabolism and tumorigenic functions in non-small cell lung cancer cells. Can Res.

[233] Dekhne AS, Shah K, Ducker GS, Katinas JM, Wong-Roushar J, Nayeen MJ, Doshi A, Ning C, Bao X, Frühauf J (2019). Novel pyrrolo[3,2-]pyrimidine compounds target mitochondrial and cytosolic one-carbon metabolism with broad-spectrum antitumor efficacy. Mol Cancer Ther.

[234] Ducker GS, Ghergurovich JM, Mainolfi N, Suri V, Jeong SK, Hsin-Jung Li S, Friedman A, Manfredi MG, Gitai Z, Kim H (2017). Human SHMT inhibitors reveal defective glycine import as a targetable metabolic vulnerability of diffuse large B-cell lymphoma. Proc Natl Acad Sci U S A.

[235] Paiardini A, Fiascarelli A, Rinaldo S, Daidone F, Giardina G, Koes DR, Parroni A, Montini G, Marani M, Paone A (2015). Screening and in vitro testing of antifolate inhibitors of human cytosolic serine hydroxymethyltransferase. ChemMedChem.

[236] Jin R, Liu B, Liu X, Fan Y, Peng W, Huang C, Marcus A, Sica G, Gilbert-Ross M, Liu Y (2021). Leflunomide suppresses the growth of LKB1-inactivated tumors in the immune-competent host and attenuates distant cancer metastasis. Mol Cancer Ther.

[237] Rosenzweig M, Palmer J, Tsai N-C, Synold T, Wu X, Tao S, Hammond SN, Buettner R, Duarte L, Htut M (2020). Repurposing leflunomide for relapsed/refractory multiple myeloma: a phase 1 study. Leuk Lymphoma.

[238] Molina JR, Sun Y, Protopopova M, Gera S, Bandi M, Bristow C, McAfoos T, Morlacchi P, Ackroyd J, Agip A-NA, et al. An inhibitor of oxidative phosphorylation exploits cancer vulnerability. Nat Med. 2018;24:1036–46. 10.1038/s41591-018-0052-4 29892070

[239] Pardee TS, Anderson RG, Pladna KM, Isom S, Ghiraldeli LP, Miller LD, Chou JW, Jin G, Zhang W, Ellis LR (2018). A phase I Study of CPI-613 in combination with high-dose cytarabine and mitoxantrone for relapsed or refractory acute myeloid leukemia. Clin Cancer Res.

[240] Galan-Cobo A, Sitthideatphaiboon P, Qu X, Poteete A, Pisegna MA, Tong P, Chen P-H, Boroughs LK, Rodriguez MLM, Zhang W (2019). LKB1 and KEAP1/NRF2 pathways cooperatively promote metabolic reprogramming with enhanced glutamine dependence in -mutant lung adenocarcinoma. Cancer Res.

[241] Nishi K, Suzuki M, Yamamoto N, Matsumoto A, Iwase Y, Yamasaki K, Otagiri M, Yumita N (2018). Glutamine deprivation enhances acetyl-CoA carboxylase inhibitor-induced death of human pancreatic cancer cells. Anticancer Res.

[242] Jain S, Hu C, Kluza J, Ke W, Tian G, Giurgiu M, Bleilevens A, Campos AR, Charbono A, Stickeler E, et al. Metabolic targeting of cancer by a ubiquinone uncompetitive inhibitor of mitochondrial complex I. Cell Chem Biol. 2021. 10.1016/j.chembiol.2021.11.002 34852219

[243] Liu Y, Zhang X, Zhou M, Nan X, Chen X, Zhang X (2017). Mitochondrial-targeting lonidamine-doxorubicin nanoparticles for synergistic chemotherapy to conquer drug resistance. ACS Appl Mater Interfaces.

[244] Abraham I, Wolf CL, Sampson KE (1993). Non-glucocorticoid steroid analogues (21-aminosteroids) sensitize multidrug resistant cells to vinblastine. Cancer Chemother Pharmacol.

[245] Ohshima Y, Takata N, Suzuki-Karasaki M, Yoshida Y, Tokuhashi Y, Suzuki-Karasaki Y (2017). Disrupting mitochondrial Ca2+ homeostasis causes tumor-selective TRAIL sensitization through mitochondrial network abnormalities. Int J Oncol.

[246] Kang MH, Reynolds CP (2009). Bcl-2 inhibitors: targeting mitochondrial apoptotic pathways in cancer therapy. Clin Cancer Res.

[247] Liu L, Qi L, Knifley T, Piecoro DW, Rychahou P, Liu J, Mitov MI, Martin J, Wang C, Wu J (2019). S100A4 alters metabolism and promotes invasion of lung cancer cells by up-regulating mitochondrial complex I protein NDUFS2. J Biol Chem.

[248] Dong L, Neuzil J (2019). Targeting mitochondria as an anticancer strategy. Cancer Commun (Lond).

[249] Simons AL, Ahmad IM, Mattson DM, Dornfeld KJ, Spitz DR (2007). 2-Deoxy-D-glucose combined with cisplatin enhances cytotoxicity via metabolic oxidative stress in human head and neck cancer cells. Can Res.

[250] Murphy MP. How mitochondria produce reactive oxygen species. Biochem J. 2009;417. 10.1042/BJ20081386 PMC2605959 19061483

[251] Lemarie A, Grimm S (2011). Mitochondrial respiratory chain complexes: apoptosis sensors mutated in cancer?. Oncogene.

[252] Pang Y, Lu Y, Caisova V, Liu Y, Bullova P, Huynh T-T, Zhou Y, Yu D, Frysak Z, Hartmann I (2018). Targeting NAD/PARP DNA repair pathway as a novel therapeutic approach to -Mutated cluster I pheochromocytoma and paraganglioma. Clin Cancer Res.

[253] Fong W, To KKW (2019). Drug repurposing to overcome resistance to various therapies for colorectal cancer. Cell Mol Life Sci: CMLS.

[254] Marinello PC, Panis C, Silva TNX, Binato R, Abdelhay E, Rodrigues JA, Mencalha AL, Lopes NMD, Luiz RC, Cecchini R (2019). Metformin prevention of doxorubicin resistance in MCF-7 and MDA-MB-231 involves oxidative stress generation and modulation of cell adaptation genes. Sci Rep.

[255] Lee JO, Kang MJ, Byun WS, Kim SA, Seo IH, Han JA, Moon JW, Kim JH, Kim SJ, Lee EJ (2019). Metformin overcomes resistance to cisplatin in triple-negative breast cancer (TNBC) cells by targeting RAD51. Breast Cancer Res.

[256] Mynhardt C, Damelin LH, Jivan R, Peres J, Prince S, Veale RB, Mavri-Damelin D (2018). Metformin-induced alterations in nucleotide metabolism cause 5-fluorouracil resistance but gemcitabine susceptibility in oesophageal squamous cell carcinoma. J Cell Biochem.

[257] Arrieta O, Barrón F, Padilla M-ÁS, Avilés-Salas A, Ramírez-Tirado LA, Arguelles Jiménez MJ, Vergara E, Zatarain-Barrón ZL, Hernández-Pedro N, Cardona AF, et al. Effect of metformin plus tyrosine kinase inhibitors compared with tyrosine kinase inhibitors alone in patients with epidermal growth factor receptor-mutated lung adenocarcinoma: a phase 2 randomized clinical trial. JAMA Oncol. 2019;5:e192553. 10.1001/jamaoncol.2019.2553 PMC6735425 31486833

[258] Beloueche-Babari M, Wantuch S, Casals Galobart T, Koniordou M, Parkes HG, Arunan V, Chung Y-L, Eykyn TR, Smith PD, Leach MO (2017). MCT1 inhibitor AZD3965 increases mitochondrial metabolism, facilitating combination therapy and noninvasive magnetic resonance spectroscopy. Can Res.

[259] Dong LF, Low P, Dyason JC, Wang XF, Prochazka L, Witting PK, Freeman R, Swettenham E, Valis K, Liu J (2008). Alpha-tocopheryl succinate induces apoptosis by targeting ubiquinone-binding sites in mitochondrial respiratory complex II. Oncogene.

[260] Liang D, Wang A-T, Yang Z-Z, Liu Y-J, Qi X-R (2015). Enhance cancer cell recognition and overcome drug resistance using hyaluronic acid and α-tocopheryl succinate based multifunctional nanoparticles. Mol Pharm.

[261] Truksa J, Dong L-F, Rohlena J, Stursa J, Vondrusova M, Goodwin J, Nguyen M, Kluckova K, Rychtarcikova Z, Lettlova S (2015). Mitochondrially targeted vitamin E succinate modulates expression of mitochondrial DNA transcripts and mitochondrial biogenesis. Antioxid Redox Signal.

[262] Yan B, Stantic M, Zobalova R, Bezawork-Geleta A, Stapelberg M, Stursa J, Prokopova K, Dong L, Neuzil J (2015). Mitochondrially targeted vitamin E succinate efficiently kills breast tumour-initiating cells in a complex II-dependent manner. BMC Cancer.

[263] Yu T-J, Hsieh C-Y, Tang J-Y, Lin L-C, Huang H-W, Wang H-R, Yeh Y-C, Chuang Y-T, Ou-Yang F, Chang H-W (2020). Antimycin A shows selective antiproliferation to oral cancer cells by oxidative stress-mediated apoptosis and DNA damage. Environ Toxicol.

[264] Han YH, Park WH (2009). Growth inhibition in antimycin A treated-lung cancer Calu-6 cells via inducing a G1 phase arrest and apoptosis. Lung Cancer (Amsterdam, Netherlands).

[265] Chu M, Zheng C, Chen C, Song G, Hu X, Wang Z-W. Targeting cancer stem cells by nutraceuticals for cancer therapy. Seminars Cancer Biol. 2021. 10.1016/j.semcancer.2021.07.008 34273521

[266] Chakraborty PK, Mustafi SB, Ganguly S, Chatterjee M, Raha S (2008). Resveratrol induces apoptosis in K562 (chronic myelogenous leukemia) cells by targeting a key survival protein, heat shock protein 70. Cancer Sci.

[267] Rauf A, Imran M, Butt MS, Nadeem M, Peters DG, Mubarak MS (2018). Resveratrol as an anti-cancer agent: a review. Crit Rev Food Sci Nutr.

[268] You K-R, Wen J, Lee S-T, Kim D-G (2002). Cytochrome c oxidase subunit III: a molecular marker for N-(4-hydroxyphenyl)retinamise-induced oxidative stress in hepatoma cells. J Biol Chem.

[269] Villani MG, Appierto V, Cavadini E, Bettiga A, Prinetti A, Clagett-Dame M, Curley RW, Formelli F (2006). 4-Oxo-fenretinide, a recently identified fenretinide metabolite, induces marked G2-M cell cycle arrest and apoptosis in fenretinide-sensitive and fenretinide-resistant cell lines. Can Res.

[270] Fazi B, Bursch W, Fimia GM, Nardacci R, Piacentini M, Di Sano F, Piredda L (2008). Fenretinide induces autophagic cell death in caspase-defective breast cancer cells. Autophagy.

[271] Kang MH, Wan Z, Kang YH, Sposto R, Reynolds CP (2008). Mechanism of synergy of N-(4-hydroxyphenyl)retinamide and ABT-737 in acute lymphoblastic leukemia cell lines: Mcl-1 inactivation. J Natl Cancer Inst.

[272] Livingstone E, Swann S, Lilla C, Schadendorf D, Roesch A (2015). Combining BRAF(V) (600E) inhibition with modulators of the mitochondrial bioenergy metabolism to overcome drug resistance in metastatic melanoma. Exp Dermatol.

[273] Tinhofer I, Bernhard D, Senfter M, Anether G, Loeffler M, Kroemer G, Kofler R, Csordas A, Greil R (2001). Resveratrol, a tumor-suppressive compound from grapes, induces apoptosis via a novel mitochondrial pathway controlled by Bcl-2. FASEB J.

[274] Shi Y, Lim SK, Liang Q, Iyer SV, Wang H-Y, Wang Z, Xie X, Sun D, Chen Y-J, Tabar V (2019). Gboxin is an oxidative phosphorylation inhibitor that targets glioblastoma. Nature.

[275] Okon IS, Zou M-H (2015). Mitochondrial ROS and cancer drug resistance: implications for therapy. Pharmacol Res.

[276] Noh MR, Kong MJ, Han SJ, Kim JI, Park KM (2020). Isocitrate dehydrogenase 2 deficiency aggravates prolonged high-fat diet intake-induced hypertension. Redox Biol.

[277] Han SJ, Choi HS, Kim JI, Park J-W, Park KM (2018). IDH2 deficiency increases the liver susceptibility to ischemia-reperfusion injury via increased mitochondrial oxidative injury. Redox Biol.

[278] Dobrachinski F, da Silva MH, Tassi CLC, de Carvalho NR, Dias GRM, Golombieski RM, da Silva Loreto EL, da Rocha JBT, Fighera MR, Soares FAA (2014). Neuroprotective effect of diphenyl diselenide in a experimental stroke model: maintenance of redox system in mitochondria of brain regions. Neurotox Res.

[279] Chalker J, Gardiner D, Kuksal N, Mailloux RJ (2018). Characterization of the impact of glutaredoxin-2 (GRX2) deficiency on superoxide/hydrogen peroxide release from cardiac and liver mitochondria. Redox Biol.

[280] Kameritsch P, Singer M, Nuernbergk C, Rios N, Reyes AM, Schmidt K, Kirsch J, Schneider H, Müller S, Pogoda K, et al. The mitochondrial thioredoxin reductase system (TrxR2) in vascular endothelium controls peroxynitrite levels and tissue integrity. Proc Natl Acad Sci USA. 2021;118. 10.1073/pnas.1921828118 PMC7896346 33579817

[281] Sullivan LB, Martinez-Garcia E, Nguyen H, Mullen AR, Dufour E, Sudarshan S, Licht JD, Deberardinis RJ, Chandel NS (2013). The proto-oncometabolite fumarate binds glutathione to amplify ROS-dependent signaling. Mol Cell.

[282] Chen J, Wong HS, Ko KM (2015). Mitochondrial reactive oxygen species production mediates ursolic acid-induced mitochondrial uncoupling and glutathione redox cycling, with protection against oxidant injury in H9c2 cells. Food Funct.

[283] Okon IS, Coughlan KA, Zhang M, Wang Q, Zou M-H (2015). Gefitinib-mediated reactive oxygen specie (ROS) instigates mitochondrial dysfunction and drug resistance in lung cancer cells. J Biol Chem.

[284] Dekhne AS, Ning C, Nayeen MJ, Shah K, Kalpage H, Frühauf J, Wallace-Povirk A, O'Connor C, Hou Z, Kim S, et al. Cellular pharmacodynamics of a novel pyrrolo[3,2-]pyrimidine inhibitor targeting mitochondrial and cytosolic one-carbon metabolism. Mol Pharmacol. 2020;97. 10.1124/mol.119.117937 PMC6877291 31707355

[285] Jiang J, Peng L, Wang K, Huang C. Moonlighting metabolic enzymes in cancer: new perspectives on the redox code. Antioxid Redox Signal. 2021;34. 10.1089/ars.2020.8123 32631077

[286] Peng L, Jiang J, Chen H-N, Zhou L, Huang Z, Qin S, Jin P, Luo M, Li B, Shi J (2021). Redox-sensitive cyclophilin A elicits chemoresistance through realigning cellular oxidative status in colorectal cancer. Cell Rep.

[287] Minton DR, Nam M, McLaughlin DJ, Shin J, Bayraktar EC, Alvarez SW, Sviderskiy VO, Papagiannakopoulos T, Sabatini DM, Birsoy K, et al. Serine catabolism by SHMT2 is required for proper mitochondrial translation initiation and maintenance of formylmethionyl-tRNAs. Mol Cell. 2018;69. 10.1016/j.molcel.2018.01.024 PMC5819360 29452640

[288] Stuart SD, Schauble A, Gupta S, Kennedy AD, Keppler BR, Bingham PM, Zachar Z (2014). A strategically designed small molecule attacks alpha-ketoglutarate dehydrogenase in tumor cells through a redox process. Cancer Metab.

[289] Pardee TS, Lee K, Luddy J, Maturo C, Rodriguez R, Isom S, Miller LD, Stadelman KM, Levitan D, Hurd D (2014). A phase I study of the first-in-class antimitochondrial metabolism agent, CPI-613, in patients with advanced hematologic malignancies. Clinical Cancer Res.

[290] Cenigaonandia-Campillo A, Serna-Blasco R, Gómez-Ocabo L, Solanes-Casado S, Baños-Herraiz N, Puerto-Nevado LD, Cañas JA, Aceñero MJ, García-Foncillas J, Aguilera Ó (2021). Vitamin C activates pyruvate dehydrogenase (PDH) targeting the mitochondrial tricarboxylic acid (TCA) cycle in hypoxic mutant colon cancer. Theranostics.

[291] Altman BJ, Stine ZE, Dang CV (2016). From Krebs to clinic: glutamine metabolism to cancer therapy. Nat Rev Cancer.

[292] Hui S, Ghergurovich JM, Morscher RJ, Jang C, Teng X, Lu W, Esparza LA, Reya T, Le Z, Yanxiang Guo J (2017). Glucose feeds the TCA cycle via circulating lactate. Nature.

[293] Romero R, Sayin VI, Davidson SM, Bauer MR, Singh SX, LeBoeuf SE, Karakousi TR, Ellis DC, Bhutkar A, Sánchez-Rivera FJ (2017). Keap1 loss promotes Kras-driven lung cancer and results in dependence on glutaminolysis. Nat Med.

[294] Shroff EH, Eberlin LS, Dang VM, Gouw AM, Gabay M, Adam SJ, Bellovin DI, Tran PT, Philbrick WM, Garcia-Ocana A (2015). MYC oncogene overexpression drives renal cell carcinoma in a mouse model through glutamine metabolism. Proc Natl Acad Sci USA.

[295] Xiang Y, Stine ZE, Xia J, Lu Y, O'Connor RS, Altman BJ, Hsieh AL, Gouw AM, Thomas AG, Gao P (2015). Targeted inhibition of tumor-specific glutaminase diminishes cell-autonomous tumorigenesis. J Clin Investig.

[296] Rajeshkumar NV, Yabuuchi S, Pai SG, De Oliveira E, Kamphorst JJ, Rabinowitz JD, Tejero H, Al-Shahrour F, Hidalgo M, Maitra A (2017). Treatment of pancreatic cancer patient-derived xenograft panel with metabolic inhibitors reveals efficacy of phenformin. Clin Cancer Res.

[297] Seltzer MJ, Bennett BD, Joshi AD, Gao P, Thomas AG, Ferraris DV, Tsukamoto T, Rojas CJ, Slusher BS, Rabinowitz JD (2010). Inhibition of glutaminase preferentially slows growth of glioma cells with mutant IDH1. Can Res.

[298] Stalnecker CA, Erickson JW, Cerione RA (2017). Conformational changes in the activation loop of mitochondrial glutaminase C: a direct fluorescence readout that distinguishes the binding of allosteric inhibitors from activators. J Biol Chem.

[299] Chakrabarti G, Moore ZR, Luo X, Ilcheva M, Ali A, Padanad M, Zhou Y, Xie Y, Burma S, Scaglioni PP (2015). Targeting glutamine metabolism sensitizes pancreatic cancer to PARP-driven metabolic catastrophe induced by ß-lapachone. Cancer Metab.

[300] Bravo R, Vicencio JM, Parra V, Troncoso R, Munoz JP, Bui M, Quiroga C, Rodriguez AE, Verdejo HE, Ferreira J (2011). Increased ER-mitochondrial coupling promotes mitochondrial respiration and bioenergetics during early phases of ER stress. J Cell Sci.

[301] O-Uchi J, Jhun BS, Hurst S, Bisetto S, Gross P, Chen M, Kettlewell S, Park J, Oyamada H, Smith GL, et al. Overexpression of ryanodine receptor type 1 enhances mitochondrial fragmentation and Ca2+-induced ATP production in cardiac H9c2 myoblasts. Am J Physiol Heart Circulatory Physiol. 2013;305:H1736–H51. 10.1152/ajpheart.00094.2013 PMC3882548 24124188

[302] Chen X, Zhang X, Kubo H, Harris DM, Mills GD, Moyer J, Berretta R, Potts ST, Marsh JD, Houser SR (2005). Ca2+ influx-induced sarcoplasmic reticulum Ca2+ overload causes mitochondrial-dependent apoptosis in ventricular myocytes. Circ Res.

[303] Takata N, Ohshima Y, Suzuki-Karasaki M, Yoshida Y, Tokuhashi Y, Suzuki-Karasaki Y (2017). Mitochondrial Ca2+ removal amplifies TRAIL cytotoxicity toward apoptosis-resistant tumor cells via promotion of multiple cell death modalities. Int J Oncol.

[304] Del Bufalo D, Biroccio A, Soddu S, Laudonio N, D'Angelo C, Sacchi A, Zupi G (1996). Lonidamine induces apoptosis in drug-resistant cells independently of the p53 gene. J Clin Investig.

[305] Xu L, Xie Q, Qi L, Wang C, Xu N, Liu W, Yu Y, Li S, Xu Y (2018). Bcl-2 overexpression reduces cisplatin cytotoxicity by decreasing ER-mitochondrial Ca2+ signaling in SKOV3 cells. Oncol Rep.

[306] Weinberg SE, Chandel NS. Targeting mitochondria metabolism for cancer therapy. Nat Chem Biol. 2015;11. 10.1038/nchembio.1712 PMC4340667 25517383

[307] Zhang E, Zhang C, Su Y, Cheng T, Shi C (2011). Newly developed strategies for multifunctional mitochondria-targeted agents in cancer therapy. Drug Discovery Today.

[308] Eakins J, Bauch C, Woodhouse H, Park B, Bevan S, Dilworth C, Walker P (2016). A combined in vitro approach to improve the prediction of mitochondrial toxicants. Toxicol Vitro.

[309] Dowling RJO, Niraula S, Stambolic V, Goodwin PJ (2012). Metformin in cancer: translational challenges. J Mol Endocrinol.

[310] Nguyen C, Pandey S. Exploiting mitochondrial vulnerabilities to trigger apoptosis selectively in cancer cells. Cancers. 2019;11. 10.3390/cancers11070916 PMC6678564 31261935

[311] Fidalgo LM, Gille L (2011). Mitochondria and trypanosomatids: targets and drugs. Pharm Res.

[312] Le W, Sayana P, Jankovic J. Animal models of Parkinson's disease: a gateway to therapeutics? Neurotherapeutics. 2014;11. 10.1007/s13311-013-0234-1 PMC3899493 24158912

[313] Brown TP, Rumsby PC, Capleton AC, Rushton L, Levy LS (2006). Pesticides and Parkinson's disease–is there a link?. Environ Health Perspect.

[314] Zhao B, Shi Z (2017). Copper-catalyzed intermolecular heck-like coupling of cyclobutanone oximes initiated by selective C–C bond cleavage. Angew Chem Int Ed Engl.

[315] Wu X, Riedel J, Dong VM (2017). Transforming olefins into γ, δ-unsaturated nitriles through copper catalysis. Angew Chem Int Ed Engl.

[316] Zhang L, Yao Y, Zhang S, Liu Y, Guo H, Ahmed M, Bell T, Zhang H, Han G, Lorence E, et al. Metabolic reprogramming toward oxidative phosphorylation identifies a therapeutic target for mantle cell lymphoma. Sci Transl Med. 2019;11. 10.1126/scitranslmed.aau1167 31068440

[317] Luengo A, Gui DY, Vander Heiden MG (2017). Targeting metabolism for cancer therapy. Cell Chem Biol.

[318] Davidson SM, Papagiannakopoulos T, Olenchock BA, Heyman JE, Keibler MA, Luengo A, Bauer MR, Jha AK, O'Brien JP, Pierce KA (2016). Environment impacts the metabolic dependencies of ras-driven non-small cell lung cancer. Cell Metab.

[319] He H, Lin X, Guo J, Wang J, Xu B (2020). Perimitochondrial enzymatic self-assembly for selective targeting the mitochondria of cancer cells. ACS Nano.

[320] Errico A (2014). Targeted therapy: targeting mitochondria in pancreatic cancer. Nat Rev Clin Oncol.

[321] Chang J-C, Chang H-S, Wu Y-C, Cheng W-L, Lin T-T, Chang H-J, Kuo S-J, Chen S-T, Liu C-S (2019). Mitochondrial transplantation regulates antitumour activity, chemoresistance and mitochondrial dynamics in breast cancer. J Exp Clin Cancer Res.

[322] Nosengo N (2016). Can you teach old drugs new tricks?. Nature.

[323] Kurzrock R, Kantarjian HM, Kesselheim AS, Sigal EV (2020). New drug approvals in oncology. Nat Rev Clin Oncol.

[324] Zhang Z, Zhou L, Xie N, Nice EC, Zhang T, Cui Y, Huang C (2020). Overcoming cancer therapeutic bottleneck by drug repurposing. Signal Transduct Target Ther.

[325] Pushpakom S, Iorio F, Eyers PA, Escott KJ, Hopper S, Wells A, Doig A, Guilliams T, Latimer J, McNamee C (2019). Drug repurposing: progress, challenges and recommendations. Nat Rev Drug Discovery.

[326] Corsello SM, Bittker JA, Liu Z, Gould J, McCarren P, Hirschman JE, Johnston SE, Vrcic A, Wong B, Khan M (2017). The Drug Repurposing Hub: a next-generation drug library and information resource. Nat Med.

[327] Pantziarka P (2017). Scientific advice - is drug repurposing missing a trick?. Nat Rev Clin Oncol.

[328] Sohraby F, Aryapour H (2021). Rational drug repurposing for cancer by inclusion of the unbiased molecular dynamics simulation in the structure-based virtual screening approach: challenges and breakthroughs. Semin Cancer Biol.

[329] Guo W-F, Zhang S-W, Feng Y-H, Liang J, Zeng T, Chen L (2021). Network controllability-based algorithm to target personalized driver genes for discovering combinatorial drugs of individual patients. Nucleic Acids Res.

[330] Issa NT, Stathias V, Schürer S, Dakshanamurthy S (2021). Machine and deep learning approaches for cancer drug repurposing. Semin Cancer Biol.

[331] Mottini C, Napolitano F, Li Z, Gao X, Cardone L (2021). Computer-aided drug repurposing for cancer therapy: approaches and opportunities to challenge anticancer targets. Semin Cancer Biol.

[332] Heckman-Stoddard BM, DeCensi A, Sahasrabuddhe VV, Ford LG (2017). Repurposing metformin for the prevention of cancer and cancer recurrence. Diabetologia.

[333] Chen K, Qian W, Jiang Z, Cheng L, Li J, Sun L, Zhou C, Gao L, Lei M, Yan B (2017). Metformin suppresses cancer initiation and progression in genetic mouse models of pancreatic cancer. Mol Cancer.

[334] Kang J, Jeong S-M, Shin DW, Cho M, Cho JH, Kim J (2021). The associations of aspirin, statins, and metformin with lung cancer risk and related mortality: a time-dependent analysis of population-based nationally representative data. J Thoracic Oncol.

[335] Emami Riedmaier A, Fisel P, Nies AT, Schaeffeler E, Schwab M (2013). Metformin and cancer: from the old medicine cabinet to pharmacological pitfalls and prospects. Trends Pharmacol Sci.

[336] Wang C, Jeong K, Jiang H, Guo W, Gu C, Lu Y, Liang J. YAP/TAZ regulates the insulin signaling via IRS1/2 in endometrial cancer. Am J Cancer Res. 2016;6. PMC4889715 27293994

[337] Hirsch HA, Iliopoulos D, Struhl K (2013). Metformin inhibits the inflammatory response associated with cellular transformation and cancer stem cell growth. Proc Natl Acad Sci USA.

[338] Zheng L, Yang W, Wu F, Wang C, Yu L, Tang L, Qiu B, Li Y, Guo L, Wu M (2013). Prognostic significance of AMPK activation and therapeutic effects of metformin in hepatocellular carcinoma. Clinical Cancer Res.

[339] Li L, Wang T, Hu M, Zhang Y, Chen H, Xu L (2020). Metformin overcomes acquired resistance to EGFR TKIs in EGFR-mutant lung cancer via AMPK/ERK/NF-κB signaling pathway. Front Oncol.

[340] Chai X, Chu H, Yang X, Meng Y, Shi P, Gou S (2015). Metformin increases sensitivity of pancreatic cancer cells to gemcitabine by reducing CD133+ cell populations and suppressing ERK/P70S6K signaling. Sci Rep.

[341] Melnik S, Dvornikov D, Müller-Decker K, Depner S, Stannek P, Meister M, Warth A, Thomas M, Muley T, Risch A (2018). Cancer cell specific inhibition of Wnt/β-catenin signaling by forced intracellular acidification. Cell Discovery.

[342] Rossini M, Martini F, Torreggiani E, Fortini F, Aquila G, Sega FVD, Patergnani S, Pinton P, Maniscalco P, Cavallesco G (2020). Metformin induces apoptosis and inhibits Notch1 in malignant pleural mesothelioma cells. Front Cell Dev Biol.

[343] Xiao H, Zhang J, Xu Z, Feng Y, Zhang M, Liu J, Chen R, Shen J, Wu J, Lu Z (2016). Metformin is a novel suppressor for transforming growth factor (TGF)-β1. Sci Rep.

[344] Zou J, Li C, Jiang S, Luo L, Yan X, Huang D, Luo Z (2021). AMPK inhibits Smad3-mediated autoinduction of TGF-β1 in gastric cancer cells. J Cell Mol Med.

[345] Wheaton WW, Weinberg SE, Hamanaka RB, Soberanes S, Sullivan LB, Anso E, Glasauer A, Dufour E, Mutlu GM, Budigner GS, et al. Metformin inhibits mitochondrial complex I of cancer cells to reduce tumorigenesis. eLife. 2014;3:e02242. 10.7554/eLife.02242 PMC4017650 24843020

[346] Kalyanaraman B, Cheng G, Hardy M, Ouari O, Lopez M, Joseph J, Zielonka J, Dwinell MB (2018). A review of the basics of mitochondrial bioenergetics, metabolism, and related signaling pathways in cancer cells: therapeutic targeting of tumor mitochondria with lipophilic cationic compounds. Redox Biol.

[347] Madiraju AK, Erion DM, Rahimi Y, Zhang X-M, Braddock DT, Albright RA, Prigaro BJ, Wood JL, Bhanot S, MacDonald MJ (2014). Metformin suppresses gluconeogenesis by inhibiting mitochondrial glycerophosphate dehydrogenase. Nature.

[348] Andrzejewski S, Gravel S-P, Pollak M, St-Pierre J (2014). Metformin directly acts on mitochondria to alter cellular bioenergetics. Cancer Metab.

[349] Griss T, Vincent EE, Egnatchik R, Chen J, Ma EH, Faubert B, Viollet B, DeBerardinis RJ, Jones RG (2015). Metformin antagonizes cancer cell proliferation by suppressing mitochondrial-dependent biosynthesis. PLoS Biol.

[350] Cheng G, Zielonka J, Ouari O, Lopez M, McAllister D, Boyle K, Barrios CS, Weber JJ, Johnson BD, Hardy M (2016). Mitochondria-targeted analogues of metformin exhibit enhanced antiproliferative and radiosensitizing effects in pancreatic cancer cells. Can Res.

[351] Liu X, Romero IL, Litchfield LM, Lengyel E, Locasale JW (2016). Metformin targets central carbon metabolism and reveals mitochondrial requirements in human cancers. Cell Metab.

[352] Groenendijk FH, Mellema WW, van der Burg E, Schut E, Hauptmann M, Horlings HM, Willems SM, van den Heuvel MM, Jonkers J, Smit EF (2015). Sorafenib synergizes with metformin in NSCLC through AMPK pathway activation. Int J Cancer.

[353] Lam TG, Jeong YS, Kim S-A, Ahn S-G (2018). New metformin derivative HL156A prevents oral cancer progression by inhibiting the insulin-like growth factor/AKT/mammalian target of rapamycin pathways. Cancer Sci.

[354] Di Magno L, Manni S, Di Pastena F, Coni S, Macone A, Cairoli S, Sambucci M, Infante P, Moretti M, Petroni M, et al. Phenformin inhibits hedgehog-dependent tumor growth through a complex I-independent redox/corepressor module. Cell Rep. 2020;30. 10.1016/j.celrep.2020.01.024 32049007

[355] de Mey S, Jiang H, Corbet C, Wang H, Dufait I, Law K, Bastien E, Verovski V, Gevaert T, Feron O (2018). Antidiabetic biguanides radiosensitize hypoxic colorectal cancer cells through a decrease in oxygen consumption. Front Pharmacol.

[356] Miskimins WK, Ahn HJ, Kim JY, Ryu S, Jung Y-S, Choi JY (2014). Synergistic anti-cancer effect of phenformin and oxamate. PLoS ONE.

[357] Zhang Y, Cheng J, Li J, He J, Li X, Xu F (2021). The GLP-1R agonist exendin-4 attenuates hyperglycemia-induced chemoresistance in human endometrial cancer cells through ROS-mediated mitochondrial pathway. Front Oncol.

[358] Villani LA, Smith BK, Marcinko K, Ford RJ, Broadfield LA, Green AE, Houde VP, Muti P, Tsakiridis T, Steinberg GR (2016). The diabetes medication Canagliflozin reduces cancer cell proliferation by inhibiting mitochondrial complex-I supported respiration. Mol Metab.

[359] Gottfried E, Rogenhofer S, Waibel H, Kunz-Schughart LA, Reichle A, Wehrstein M, Peuker A, Peter K, Hartmannsgruber G, Andreesen R (2011). Pioglitazone modulates tumor cell metabolism and proliferation in multicellular tumor spheroids. Cancer Chemother Pharmacol.

[360] Lu Z, Xu N, He B, Pan C, Lan Y, Zhou H, Liu X (2017). Inhibition of autophagy enhances the selective anti-cancer activity of tigecycline to overcome drug resistance in the treatment of chronic myeloid leukemia. J Exp Clin Cancer Res: CR.

[361] Norberg E, Lako A, Chen P-H, Stanley IA, Zhou F, Ficarro SB, Chapuy B, Chen L, Rodig S, Shin D (2017). Differential contribution of the mitochondrial translation pathway to the survival of diffuse large B-cell lymphoma subsets. Cell Death Differ.

[362] Wang B, Ao J, Yu D, Rao T, Ruan Y, Yao X (2017). Inhibition of mitochondrial translation effectively sensitizes renal cell carcinoma to chemotherapy. Biochem Biophys Res Commun.

[363] Hu B, Guo Y (2019). Inhibition of mitochondrial translation as a therapeutic strategy for human ovarian cancer to overcome chemoresistance. Biochem Biophys Res Commun.

[364] Song M, Wu H, Wu S, Ge T, Wang G, Zhou Y, Sheng S, Jiang J. Antibiotic drug levofloxacin inhibits proliferation and induces apoptosis of lung cancer cells through inducing mitochondrial dysfunction and oxidative damage. Biomed Pharmacother. 2016;84:1137–43. 10.1016/j.biopha.2016.10.034 27780143

[365] Yu K, Wang T, Li Y, Wang C, Wang X, Zhang M, Xie Y, Li S, An Z, Ye T. Niclosamide induces apoptosis through mitochondrial intrinsic pathway and inhibits migration and invasion in human thyroid cancer in vitro. Biomed Pharmacother. 2017;92:403–11. 10.1016/j.biopha.2017.05.097 28575805

[366] Yu Q-S, Xin H-R, Qiu R-L, Deng Z-L, Deng F, Yan Z-J (2020). Niclosamide: drug repurposing for human chondrosarcoma treatment via the caspase-dependent mitochondrial apoptotic pathway. Am J Transl Res.

[367] Liu Y, Fang S, Sun Q, Liu B (2016). Anthelmintic drug ivermectin inhibits angiogenesis, growth and survival of glioblastoma through inducing mitochondrial dysfunction and oxidative stress. Biochem Biophys Res Commun.

[368] Lin L, Lu W, Dai T, Chen H, Wang T, Yang L, Yang X, Liu Y, Sun D (2021). Novel artemisinin derivatives with potent anticancer activities and the anti-colorectal cancer effect by the mitochondria-mediated pathway. Bioorg Chem.

[369] Tsui K-H, Wu M-Y, Lin L-T, Wen Z-H, Li Y-H, Chu P-Y, Li C-J (2019). Disruption of mitochondrial homeostasis with artemisinin unravels anti-angiogenesis effects via auto-paracrine mechanisms. Theranostics.

[370] Zhang J, Sun X, Wang L, Wong YK, Lee YM, Zhou C, Wu G, Zhao T, Yang L, Lu L (2018). Artesunate-induced mitophagy alters cellular redox status. Redox Biol.

[371] Ashton TM, Fokas E, Kunz-Schughart LA, Folkes LK, Anbalagan S, Huether M, Kelly CJ, Pirovano G, Buffa FM, Hammond EM (2016). The anti-malarial atovaquone increases radiosensitivity by alleviating tumour hypoxia. Nat Commun.

[372] Kobayashi Y, Banno K, Kunitomi H, Tominaga E, Aoki D (2019). Current state and outlook for drug repositioning anticipated in the field of ovarian cancer. J Gynecologic Oncol.

[373] Weir SJ, Patton L, Castle K, Rajewski L, Kasper J, Schimmer AD (2011). The repositioning of the anti-fungal agent ciclopirox olamine as a novel therapeutic agent for the treatment of haematologic malignancy. J Clin Pharm Ther.

[374] Tsubamoto H, Ueda T, Inoue K, Sakata K, Shibahara H, Sonoda T (2017). Repurposing itraconazole as an anticancer agent. Oncol Lett.

[375] Chen K, Cheng L, Qian W, Jiang Z, Sun L, Zhao Y, Zhou Y, Zhao L, Wang P, Duan W (2018). Itraconazole inhibits invasion and migration of pancreatic cancer cells by suppressing TGF-β/SMAD2/3 signaling. Oncol Rep.

[376] Pounds R, Leonard S, Dawson C, Kehoe S (2017). Repurposing itraconazole for the treatment of cancer. Oncol Lett.

[377] Deng H, Huang L, Liao Z, Liu M, Li Q, Xu R (2020). Itraconazole inhibits the Hedgehog signaling pathway thereby inducing autophagy-mediated apoptosis of colon cancer cells. Cell Death Dis.

[378] Liu R, Li J, Zhang T, Zou L, Chen Y, Wang K, Lei Y, Yuan K, Li Y, Lan J (2014). Itraconazole suppresses the growth of glioblastoma through induction of autophagy: involvement of abnormal cholesterol trafficking. Autophagy.

[379] Head SA, Shi W, Zhao L, Gorshkov K, Pasunooti K, Chen Y, Deng Z, Li R-j, Shim JS, Tan W, et al. Antifungal drug itraconazole targets VDAC1 to modulate the AMPK/mTOR signaling axis in endothelial cells. Proc Natl Acad Sci USA. 2015;112:E7276-E85. 10.1073/pnas.1512867112 PMC4703001 26655341

[380] Head SA, Shi WQ, Yang EJ, Nacev BA, Hong SY, Pasunooti KK, Li R-J, Shim JS, Liu JO (2017). Simultaneous targeting of NPC1 and VDAC1 by itraconazole leads to synergistic inhibition of mTOR signaling and angiogenesis. ACS Chem Biol.

[381] Wang X, Wei S, Zhao Y, Shi C, Liu P, Zhang C, Lei Y, Zhang B, Bai B, Huang Y (2017). Anti-proliferation of breast cancer cells with itraconazole: hedgehog pathway inhibition induces apoptosis and autophagic cell death. Cancer Lett.

[382] Lopez-Barcons L, Maurer BJ, Kang MH, Reynolds CP (2017). P450 inhibitor ketoconazole increased the intratumor drug levels and antitumor activity of fenretinide in human neuroblastoma xenograft models. Int J Cancer.

[383] Chen Y, Chen H-N, Wang K, Zhang L, Huang Z, Liu J, Zhang Z, Luo M, Lei Y, Peng Y (2019). Ketoconazole exacerbates mitophagy to induce apoptosis by downregulating cyclooxygenase-2 in hepatocellular carcinoma. J Hepatol.

[384] Choi EK, Park EJ, Phan TT, Kim HD, Hoe K-L, Kim D-U (2020). Econazole induces p53-dependent apoptosis and decreases metastasis ability in gastric cancer cells. Biomol Therap.

[385] Sobecks R, McCormick TS, Distelhorst CW (1996). Imidazole antifungals miconazole and econazole induce apoptosis in mouse lymphoma and human T cell leukemia cells: regulation by Bcl-2 and potential role of calcium. Cell Death Differ.

[386] Zhang Y, Soboloff J, Zhu Z, Berger SA (2006). Inhibition of Ca2+ influx is required for mitochondrial reactive oxygen species-induced endoplasmic reticulum Ca2+ depletion and cell death in leukemia cells. Mol Pharmacol.

[387] Ho Y-S, Wu C-H, Chou H-M, Wang Y-J, Tseng H, Chen C-H, Chen L-C, Lee C-H, Lin S-Y (2005). Molecular mechanisms of econazole-induced toxicity on human colon cancer cells: G0/G1 cell cycle arrest and caspase 8-independent apoptotic signaling pathways. Food Chem Toxicol.

[388] Zhang J, Fan J (2020). Prazosin inhibits the proliferation, migration and invasion, but promotes the apoptosis of U251 and U87 cells via the PI3K/AKT/mTOR signaling pathway. Exp Ther Med.

[389] Spencer BH, McDermott CM, Chess-Williams R, Christie D, Anoopkumar-Dukie S (2018). Prazosin but not tamsulosin sensitises PC-3 and LNCaP prostate cancer cells to docetaxel. Pharmacology.

[390] Lin S-C, Chueh S-C, Hsiao C-J, Li T-K, Chen T-H, Liao C-H, Lyu P-C, Guh J-H (2007). Prazosin displays anticancer activity against human prostate cancers: targeting DNA and cell cycle. Neoplasia (New York, NY).

[391] Wang K, Liu R, Li J, Mao J, Lei Y, Wu J, Zeng J, Zhang T, Wu H, Chen L (2011). Quercetin induces protective autophagy in gastric cancer cells: involvement of Akt-mTOR- and hypoxia-induced factor 1α-mediated signaling. Autophagy.

[392] Reyes-Farias M, Carrasco-Pozo C. The anti-cancer effect of quercetin: molecular implications in cancer metabolism. Int J Mol Sci. 2019;20. 10.3390/ijms20133177 PMC6651418 31261749

[393] Ma T, Liu Y, Wu Q, Luo L, Cui Y, Wang X, Chen X, Tan L, Meng X (2019). Quercetin-modified metal-organic frameworks for dual sensitization of radiotherapy in tumor tissues by inhibiting the carbonic anhydrase IX. ACS Nano.

[394] Shi H, Li X-Y, Chen Y, Zhang X, Wu Y, Wang Z-X, Chen P-H, Dai H-Q, Feng J, Chatterjee S (2020). Quercetin induces apoptosis via downregulation of vascular endothelial growth factor/akt signaling pathway in acute myeloid leukemia cells. Front Pharmacol.

[395] Mouria M, Gukovskaya AS, Jung Y, Buechler P, Hines OJ, Reber HA, Pandol SJ (2002). Food-derived polyphenols inhibit pancreatic cancer growth through mitochondrial cytochrome C release and apoptosis. Int J Cancer.

[396] Zou H, Ye H, Kamaraj R, Zhang T, Zhang J, Pavek P (2021). A review on pharmacological activities and synergistic effect of quercetin with small molecule agents. Phytomedicine.

[397] Lee AR, Seo MJ, Kim J, Lee DM, Kim IY, Yoon MJ, Hoon H, Choi KS. Lercanidipine synergistically enhances bortezomib cytotoxicity in cancer cells via enhanced endoplasmic reticulum stress and mitochondrial ca overload. Int J Mol Sci. 2019;20. 10.3390/ijms20246112 PMC6941136 31817163

[398] Grahovac J, Srdić-Rajić T, Francisco Santibañez J, Pavlović M, Čavić M, Radulović S (2019). Telmisartan induces melanoma cell apoptosis and synergizes with vemurafenib by altering cell bioenergetics. Cancer Biol Med.

[399] Sperling CD, Aalborg GL, Dehlendorff C, Friis S, Mørch LS, Kjaer SK. Use of antidepressants and endometrial-cancer risk: a nationwide nested case-control study. Int J Epidemiol. 2021. 10.1093/ije/dyab200 34550389

[400] Brandes LJ (2005). Hormetic effects of hormones, antihormones, and antidepressants on cancer cell growth in culture: in vivo correlates. Crit Rev Toxicol.

[401] Rossi M, Rotblat B, Ansell K, Amelio I, Caraglia M, Misso G, Bernassola F, Cavasotto CN, Knight RA, Ciechanover A (2014). High throughput screening for inhibitors of the HECT ubiquitin E3 ligase ITCH identifies antidepressant drugs as regulators of autophagy. Cell Death Dis.

[402] Higgins SC, Pilkington GJ (2010). The in vitro effects of tricyclic drugs and dexamethasone on cellular respiration of malignant glioma. Anticancer Res.

[403] Yang WH, Su YH, Hsu WH, Wang CC, Arbiser JL, Yang MH (2016). Imipramine blue halts head and neck cancer invasion through promoting F-box and leucine-rich repeat protein 14-mediated Twist1 degradation. Oncogene.

[404] Rajamanickam S, Panneerdoss S, Gorthi A, Timilsina S, Onyeagucha B, Kovalskyy D, Ivanov D, Hanes MA, Vadlamudi RK, Chen Y (2016). Inhibition of FoxM1-mediated DNA repair by imipramine blue suppresses breast cancer growth and metastasis. Clin Cancer Res.

[405] Rodriguez-Lafrasse C, Alphonse G, Aloy M-T, Ardail D, Gérard J-P, Louisot P, Rousson R (2002). Increasing endogenous ceramide using inhibitors of sphingolipid metabolism maximizes ionizing radiation-induced mitochondrial injury and apoptotic cell killing. Int J Cancer.

[406] Lei B, Xu L, Zhang X, Peng W, Tang Q, Feng C (2021). The proliferation effects of fluoxetine and amitriptyline on human breast cancer cells and the underlying molecular mechanisms. Environ Toxicol Pharmacol.

[407] Bielecka-Wajdman AM, Ludyga T, Machnik G, Gołyszny M, Obuchowicz E (2018). Tricyclic antidepressants modulate stressed mitochondria in glioblastoma multiforme cells. Cancer control.

[408] Bielecka-Wajdman AM, Lesiak M, Ludyga T, Sieroń A, Obuchowicz E (2017). Reversing glioma malignancy: a new look at the role of antidepressant drugs as adjuvant therapy for glioblastoma multiforme. Cancer Chemother Pharmacol.

[409] Daley E, Wilkie D, Loesch A, Hargreaves IP, Kendall DA, Pilkington GJ, Bates TE (2005). Chlorimipramine: a novel anticancer agent with a mitochondrial target. Biochem Biophys Res Commun.

[410] Peer D, Dekel Y, Melikhov D, Margalit R (2004). Fluoxetine inhibits multidrug resistance extrusion pumps and enhances responses to chemotherapy in syngeneic and in human xenograft mouse tumor models. Can Res.

[411] Lee CS, Kim YJ, Jang ER, Kim W, Myung SC (2010). Fluoxetine induces apoptosis in ovarian carcinoma cell line OVCAR-3 through reactive oxygen species-dependent activation of nuclear factor-kappaB. Basic Clin Pharmacol Toxicol.

[412] Charles E, Hammadi M, Kischel P, Delcroix V, Demaurex N, Castelbou C, Vacher A-M, Devin A, Ducret T, Nunes P (2017). The antidepressant fluoxetine induces necrosis by energy depletion and mitochondrial calcium overload. Oncotarget.

[413] Yuan S-Y, Cheng C-L, Ho H-C, Wang S-S, Chiu K-Y, Su C-K, Ou Y-C, Lin C-C (2015). Nortriptyline induces mitochondria and death receptor-mediated apoptosis in bladder cancer cells and inhibits bladder tumor growth in vivo. Eur J Pharmacol.

[414] Kayarthodi S, Fujimura Y, Fang J, Morsalin S, Rao VN, Reddy ESP. Anti-epileptic drug targets ewing sarcoma. J Pharm Sci Pharmacol. 2014;1. 10.1166/jpsp.2014.1013 PMC4316750 25664332

[415] Tran LNK, Kichenadasse G, Morel KL, Lavranos TC, Klebe S, Lower KM, Ormsby RJ, Elliot DJ, Sykes PJ. The combination of metformin and valproic acid has a greater anti-tumoral effect on prostate cancer growth than either drug alone. In vivo (Athens, Greece). 2019;33. 10.21873/invivo.11445 PMC6364073 30587609

[416] Tesei A, Brigliadori G, Carloni S, Fabbri F, Ulivi P, Arienti C, Sparatore A, Del Soldato P, Pasini A, Amadori D (2012). Organosulfur derivatives of the HDAC inhibitor valproic acid sensitize human lung cancer cell lines to apoptosis and to cisplatin cytotoxicity. J Cell Physiol.

[417] Abaza M-SI, Bahman A-M, Al-Attiyah RaJ. Valproic acid, an anti-epileptic drug and a histone deacetylase inhibitor, in combination with proteasome inhibitors exerts antiproliferative, pro-apoptotic and chemosensitizing effects in human colorectal cancer cells: underlying molecular mechanisms. Int J Mol Med. 2014;34:513–32. 10.3892/ijmm.2014.1795 24899129

[418] Ziauddin MF, Yeow W-S, Maxhimer JB, Baras A, Chua A, Reddy RM, Tsai W, Cole GW, Schrump DS, Nguyen DM (2006). Valproic acid, an antiepileptic drug with histone deacetylase inhibitory activity, potentiates the cytotoxic effect of Apo2L/TRAIL on cultured thoracic cancer cells through mitochondria-dependent caspase activation. Neoplasia (New York, NY).

[419] Komulainen T, Lodge T, Hinttala R, Bolszak M, Pietilä M, Koivunen P, Hakkola J, Poulton J, Morten KJ, Uusimaa J (2015). Sodium valproate induces mitochondrial respiration dysfunction in HepG2 in vitro cell model. Toxicology.

[420] Ong CKS, Lirk P, Tan CH, Seymour RA (2007). An evidence-based update on nonsteroidal anti-inflammatory drugs. Clin Med Res.

[421] da Costa BR, Reichenbach S, Keller N, Nartey L, Wandel S, Jüni P, Trelle S (2017). Effectiveness of non-steroidal anti-inflammatory drugs for the treatment of pain in knee and hip osteoarthritis: a network meta-analysis. Lancet (London, England).

[422] Bindu S, Mazumder S, Bandyopadhyay U (2020). Non-steroidal anti-inflammatory drugs (NSAIDs) and organ damage: a current perspective. Biochem Pharmacol.

[423] Drew DA, Cao Y, Chan AT (2016). Aspirin and colorectal cancer: the promise of precision chemoprevention. Nat Rev Cancer.

[424] Hua H, Zhang H, Kong Q, Wang J, Jiang Y (2019). Complex roles of the old drug aspirin in cancer chemoprevention and therapy. Med Res Rev.

[425] Drew DA, Chan AT (2021). Aspirin in the prevention of colorectal neoplasia. Annu Rev Med.

[426] Upadhyay A, Amanullah A, Chhangani D, Joshi V, Mishra R, Mishra A (2016). Ibuprofen induces mitochondrial-mediated apoptosis through proteasomal dysfunction. Mol Neurobiol.

[427] Zhao X, Xu Z, Li H (2017). NSAIDs use and reduced metastasis in cancer patients: results from a meta-analysis. Sci Rep.

[428] Wakabayashi K. NSAIDs as Cancer preventive agents. Asian Pacific J Cancer Prevent: APJCP. 2000;1. 12718676

[429] de Groot DJA, de Vries EGE, Groen HJM, de Jong S (2007). Non-steroidal anti-inflammatory drugs to potentiate chemotherapy effects: from lab to clinic. Crit Rev Oncol Hematol.

[430] Vaish V, Piplani H, Rana C, Vaiphei K, Sanyal SN (2013). NSAIDs may regulate EGR-1-mediated induction of reactive oxygen species and non-steroidal anti-inflammatory drug-induced gene (NAG)-1 to initiate intrinsic pathway of apoptosis for the chemoprevention of colorectal cancer. Mol Cell Biochem.

[431] Crokart N, Radermacher K, Jordan BF, Baudelet C, Cron GO, Grégoire V, Beghein N, Bouzin C, Feron O, Gallez B (2005). Tumor radiosensitization by antiinflammatory drugs: evidence for a new mechanism involving the oxygen effect. Can Res.

[432] Bank A, Wang P, Du C, Yu J, Zhang L (2008). SMAC mimetics sensitize nonsteroidal anti-inflammatory drug-induced apoptosis by promoting caspase-3-mediated cytochrome c release. Can Res.

[433] Gaziano JM, Brotons C, Coppolecchia R, Cricelli C, Darius H, Gorelick PB, Howard G, Pearson TA, Rothwell PM, Ruilope LM (2018). Use of aspirin to reduce risk of initial vascular events in patients at moderate risk of cardiovascular disease (ARRIVE): a randomised, double-blind, placebo-controlled trial. Lancet (London, England).

[434] Amarenco P, Albers GW, Denison H, Easton JD, Evans SR, Held P, Hill MD, Jonasson J, Kasner SE, Ladenvall P (2017). Efficacy and safety of ticagrelor versus aspirin in acute stroke or transient ischaemic attack of atherosclerotic origin: a subgroup analysis of SOCRATES, a randomised, double-blind, controlled trial. Lancet Neurol.

[435] Zimmermann KC, Waterhouse NJ, Goldstein JC, Schuler M, Green DR (2000). Aspirin induces apoptosis through release of cytochrome c from mitochondria. Neoplasia (New York, NY).

[436] Cheng Q, Shi H, Wang H, Wang J, Liu Y (2016). Asplatin enhances drug efficacy by altering the cellular response. Metallomics.

[437] de Groot DJA, van der Deen M, Le TKP, Regeling A, de Jong S, de Vries EGE (2007). Indomethacin induces apoptosis via a MRP1-dependent mechanism in doxorubicin-resistant small-cell lung cancer cells overexpressing MRP1. Br J Cancer.

[438] Mazumder S, De R, Debsharma S, Bindu S, Maity P, Sarkar S, Saha SJ, Siddiqui AA, Banerjee C, Nag S (2019). Indomethacin impairs mitochondrial dynamics by activating the PKCζ-p38-DRP1 pathway and inducing apoptosis in gastric cancer and normal mucosal cells. J Biol Chem.

[439] Cui XY, Park SH, Park WH (2020). Auranofin inhibits the proliferation of lung cancer cells via necrosis and caspase-dependent apoptosis. Oncol Rep.

[440] Gamberi T, Chiappetta G, Fiaschi T, Modesti A, Sorbi F, Magherini F. Upgrade of an old drug: auranofin in innovative cancer therapies to overcome drug resistance and to increase drug effectiveness. Med Res Rev. 2021. 10.1002/med.21872 PMC9299597 34850406

[441] Hatem E, Azzi S, El Banna N, He T, Heneman-Masurel A, Vernis L, Baïlle D, Masson V, Dingli F, Loew D (2019). Auranofin/vitamin C: a novel drug combination targeting triple-negative breast cancer. J Natl Cancer Inst.

[442] Ryu Y-S, Shin S, An H-G, Kwon T-U, Baek H-S, Kwon Y-J, Chun Y-J (2020). Synergistic induction of apoptosis by the combination of an Axl inhibitor and auranofin in human breast cancer cells. Biomol Therap.

[443] Hu J, Zhang H, Cao M, Wang L, Wu S, Fang B (2018). Auranofin enhances ibrutinib's anticancer activity in EGFR-mutant lung adenocarcinoma. Mol Cancer Ther.

[444] Zhang X, Selvaraju K, Saei AA, D'Arcy P, Zubarev RA, Arnér ES, Linder S (2019). Repurposing of auranofin: thioredoxin reductase remains a primary target of the drug. Biochimie.

[445] Zou P, Chen M, Ji J, Chen W, Chen X, Ying S, Zhang J, Zhang Z, Liu Z, Yang S (2015). Auranofin induces apoptosis by ROS-mediated ER stress and mitochondrial dysfunction and displayed synergistic lethality with piperlongumine in gastric cancer. Oncotarget.

[446] Hsieh Y-J, Chang C-J, Wan C-F, Chen C-P, Chiu Y-H, Leu Y-L, Peng K-C (2013). Euphorbia formosana root extract induces apoptosis by caspase-dependent cell death via Fas and mitochondrial pathway in THP-1 human leukemic cells. Molecules (Basel, Switzerland).

[447] Courty Y, Morel F, Dufaure JP (1986). In vitro translation of RNA from lizard epididymis and identification of poly A RNA coding for a major androgen-dependent protein. C R Acad Sci.

[448] Mémin E, Hoque M, Jain MR, Heller DS, Li H, Cracchiolo B, Hanauske-Abel HM, Pe'ery T, Mathews MB (2014). Blocking eIF5A modification in cervical cancer cells alters the expression of cancer-related genes and suppresses cell proliferation. Can Res.

[449] Simonart T, Noel JC, Andrei G, Parent D, Van Vooren JP, Hermans P, Lunardi-Yskandar Y, Lambert C, Dieye T, Farber CM (1998). Iron as a potential co-factor in the pathogenesis of Kaposi's sarcoma?. Int J Cancer.

[450] Simões RV, Veeraperumal S, Serganova IS, Kruchevsky N, Varshavsky J, Blasberg RG, Ackerstaff E, Koutcher JA. Inhibition of prostate cancer proliferation by Deferiprone. NMR Biomed. 2017;30. 10.1002/nbm.3712 PMC5505495 28272795

[451] Fiorillo M, Tóth F, Brindisi M, Sotgia F, Lisanti MP. Deferiprone (DFP) targets cancer stem cell (CSC) propagation by inhibiting mitochondrial metabolism and inducing ROS production. Cells. 2020;9. 10.3390/cells9061529 PMC7349387 32585919

[452] Zhang X, Fryknäs M, Hernlund E, Fayad W, De Milito A, Olofsson MH, Gogvadze V, Dang L, Påhlman S, Schughart LAK (2014). Induction of mitochondrial dysfunction as a strategy for targeting tumour cells in metabolically compromised microenvironments. Nat Commun.

[453] Fryknäs M, Zhang X, Bremberg U, Senkowski W, Olofsson MH, Brandt P, Persson I, D'Arcy P, Gullbo J, Nygren P (2016). Iron chelators target both proliferating and quiescent cancer cells. Sci Rep.

[454] Urra FA, Weiss-López B, Araya-Maturana R (2016). Determinants of anti-cancer effect of mitochondrial electron transport chain inhibitors: bioenergetic profile and metabolic flexibility of cancer cells. Curr Pharm Des.

[455] Vitiello GA, Medina BD, Zeng S, Bowler TG, Zhang JQ, Loo JK, Param NJ, Liu M, Moral AJ, Zhao JN (2018). Mitochondrial inhibition augments the efficacy of imatinib by resetting the metabolic phenotype of gastrointestinal stromal tumor. Clin Cancer Res.

[456] Ekstrom TL, Pathoulas NM, Huehls AM, Kanakkanthara A, Karnitz LM (2021). VLX600 disrupts homologous recombination and synergizes with PARP inhibitors and cisplatin by inhibiting histone lysine demethylases. Mol Cancer Ther.

[457] Brewer GJ (2005). Anticopper therapy against cancer and diseases of inflammation and fibrosis. Drug Discovery Today.

[458] Xu M, Casio M, Range DE, Sosa JA, Counter CM (2018). Copper chelation as targeted therapy in a mouse model of oncogenic BRAF-driven papillary thyroid cancer. Clin Cancer Res.

[459] Hald J, Jacobsen E (1948). A drug sensitizing the organism to ethyl alcohol. Lancet (London, England).

[460] Tacconi EM, Lai X, Folio C, Porru M, Zonderland G, Badie S, Michl J, Sechi I, Rogier M, Matía García V (2017). BRCA1 and BRCA2 tumor suppressors protect against endogenous acetaldehyde toxicity. EMBO Mol Med.

[461] Ekinci E, Rohondia S, Khan R, Dou QP (2019). Repurposing disulfiram as an anti-cancer agent: updated review on literature and patents. Recent Pat Anti-Cancer Drug Discovery.

[462] Skrott Z, Majera D, Gursky J, Buchtova T, Hajduch M, Mistrik M, Bartek J (2019). Disulfiram's anti-cancer activity reflects targeting NPL4, not inhibition of aldehyde dehydrogenase. Oncogene.

[463] Ren L, Feng W, Shao J, Ma J, Xu M, Zhu B-Z, Zheng N, Liu S (2020). Diethyldithiocarbamate-copper nanocomplex reinforces disulfiram chemotherapeutic efficacy through light-triggered nuclear targeting. Theranostics.

[464] Triscott J, Rose Pambid M, Dunn SE (2015). Concise review: bullseye: targeting cancer stem cells to improve the treatment of gliomas by repurposing disulfiram. Stem Cells (Dayton, Ohio).

[465] Cen D, Gonzalez RI, Buckmeier JA, Kahlon RS, Tohidian NB, Meyskens FL (2002). Disulfiram induces apoptosis in human melanoma cells: a redox-related process. Mol Cancer Ther.

[466] Chen S-Y, Chang Y-L, Liu S-T, Chen G-S, Lee S-P, Huang S-M. Differential cytotoxicity mechanisms of copper complexed with disulfiram in oral cancer cells. Int J Mol Sci. 2021;22. 10.3390/ijms22073711 PMC8038175 33918312

[467] Kirshner JR, He S, Balasubramanyam V, Kepros J, Yang C-Y, Zhang M, Du Z, Barsoum J, Bertin J (2008). Elesclomol induces cancer cell apoptosis through oxidative stress. Mol Cancer Ther.

[468] Gao W, Huang Z, Duan J, Nice EC, Lin J, Huang C (2021). Elesclomol induces copper-dependent ferroptosis in colorectal cancer cells via degradation of ATP7A. Mol Oncol.

[469] Qu Y, Wang J, Sim M-S, Liu B, Giuliano A, Barsoum J, Cui X (2010). Elesclomol, counteracted by Akt survival signaling, enhances the apoptotic effect of chemotherapy drugs in breast cancer cells. Breast Cancer Res Treat.

[470] Hasinoff BB, Wu X, Yadav AA, Patel D, Zhang H, Wang D-S, Chen Z-S, Yalowich JC (2015). Cellular mechanisms of the cytotoxicity of the anticancer drug elesclomol and its complex with Cu(II). Biochem Pharmacol.

[471] Hasinoff BB, Yadav AA, Patel D, Wu X (2014). The cytotoxicity of the anticancer drug elesclomol is due to oxidative stress indirectly mediated through its complex with Cu(II). J Inorg Biochem.

[472] Nagai M, Vo NH, Shin Ogawa L, Chimmanamada D, Inoue T, Chu J, Beaudette-Zlatanova BC, Lu R, Blackman RK, Barsoum J (2012). The oncology drug elesclomol selectively transports copper to the mitochondria to induce oxidative stress in cancer cells. Free Radical Biol Med.

[473] Modica-Napolitano JS, Bharath LP, Hanlon AJ, Hurley LD. The anticancer agent elesclomol has direct effects on mitochondrial bioenergetic function in isolated mammalian mitochondria. Biomolecules. 2019;9. 10.3390/biom9080298 PMC6724019 31344923

[474] Galiana-Roselló C, Aceves-Luquero C, González J, Martínez-Camarena Á, Villalonga R, Fernández de Mattos S, Soriano C, Llinares J, García-España E, Villalonga P, et al. Toward a rational design of polyamine-based zinc-chelating agents for cancer therapies. J Med Chem. 2020;63:1199–215. 10.1021/acs.jmedchem.9b01554 31935092

[475] Pan X, Li R, Guo H, Zhang W, Xu X, Chen X, Ding L (2020). Dihydropyridine calcium channel blockers suppress the transcription of PD-L1 by inhibiting the activation of STAT1. Front Pharmacol.

[476] Hinz B, Ramer R (2019). Anti-tumour actions of cannabinoids. Br J Pharmacol.

[477] Mahoney JM, Harris RA (1972). Effect of 9 -tetrahydrocannabinol on mitochondrial precesses. Biochem Pharmacol.

[478] Whyte DA, Al-Hammadi S, Balhaj G, Brown OM, Penefsky HS, Souid A-K (2010). Cannabinoids inhibit cellular respiration of human oral cancer cells. Pharmacology.

[479] Rimmerman N, Ben-Hail D, Porat Z, Juknat A, Kozela E, Daniels MP, Connelly PS, Leishman E, Bradshaw HB, Shoshan-Barmatz V (2013). Direct modulation of the outer mitochondrial membrane channel, voltage-dependent anion channel 1 (VDAC1) by cannabidiol: a novel mechanism for cannabinoid-induced cell death. Cell Death Dis.

[480] Trumbeckaite S, Cesna V, Jasukaitiene A, Baniene R, Gulbinas A (2018). Different mitochondrial response to cisplatin and hyperthermia treatment in human AGS, Caco-2 and T3M4 cancer cell lines. J Bioenerg Biomembr.

[481] Aryal B, Rao VA (2016). Deficiency in cardiolipin reduces doxorubicin-induced oxidative stress and mitochondrial damage in human B-lymphocytes. PLoS ONE.

[482] Lipshultz SE, Anderson LM, Miller TL, Gerschenson M, Stevenson KE, Neuberg DS, Franco VI, LiButti DE, Silverman LB, Vrooman LM (2016). Impaired mitochondrial function is abrogated by dexrazoxane in doxorubicin-treated childhood acute lymphoblastic leukemia survivors. Cancer.

[483] Yadav N, Kumar S, Marlowe T, Chaudhary AK, Kumar R, Wang J, O'Malley J, Boland PM, Jayanthi S, Kumar TKS (2015). Oxidative phosphorylation-dependent regulation of cancer cell apoptosis in response to anticancer agents. Cell Death Dis.

[484] Bull VH, Rajalingam K, Thiede B (2012). Sorafenib-induced mitochondrial complex I inactivation and cell death in human neuroblastoma cells. J Proteome Res.

[485] Cardoso CM, Custódio JB, Almeida LM, Moreno AJ (2001). Mechanisms of the deleterious effects of tamoxifen on mitochondrial respiration rate and phosphorylation efficiency. Toxicol Appl Pharmacol.

[486] Torchilin VP (2006). Recent approaches to intracellular delivery of drugs and DNA and organelle targeting. Annu Rev Biomed Eng.

[487] Kumar R, Han J, Lim H-J, Ren WX, Lim J-Y, Kim J-H, Kim JS (2014). Mitochondrial induced and self-monitored intrinsic apoptosis by antitumor theranostic prodrug: in vivo imaging and precise cancer treatment. J Am Chem Soc.

[488] Cheng L, Wang X, Gong F, Liu T, Liu Z (2020). 2D Nanomaterials for cancer theranostic applications. Adv Mater (Deerfield Beach, Fla).

[489] Sun T, Zhang YS, Pang B, Hyun DC, Yang M, Xia Y (2014). Engineered nanoparticles for drug delivery in cancer therapy. Angew Chem Int Ed Engl.

[490] Nie W, Wu G, Zhang J, Huang L-L, Ding J, Jiang A, Zhang Y, Liu Y, Li J, Pu K (2020). Responsive exosome nano-bioconjugates for synergistic cancer therapy. Angew Chem Int Ed Engl.

[491] Guo X, Yang N, Ji W, Zhang H, Dong X, Zhou Z, Li L, Shen H-M, Yao SQ, Huang W (2021). Mito-bomb: targeting mitochondria for cancer therapy. Adv Mater.

[492] Fulda S, Galluzzi L, Kroemer G (2010). Targeting mitochondria for cancer therapy. Nat Rev Drug Discovery.

[493] Wang W, Liu J, Feng W, Du S, Ge R, Li J, Liu Y, Sun H, Zhang D, Zhang H (2019). Targeting mitochondria with Au-Ag@Polydopamine nanoparticles for papillary thyroid cancer therapy. Biomater Sci.

[494] Pathania D, Millard M, Neamati N (2009). Opportunities in discovery and delivery of anticancer drugs targeting mitochondria and cancer cell metabolism. Adv Drug Deliv Rev.

[495] Zielonka J, Joseph J, Sikora A, Hardy M, Ouari O, Vasquez-Vivar J, Cheng G, Lopez M, Kalyanaraman B (2017). Mitochondria-targeted triphenylphosphonium-based compounds: syntheses, mechanisms of action, and therapeutic and diagnostic applications. Chem Rev.

[496] Chan MS, Liu LS, Leung HM, Lo PK (2017). Cancer-cell-specific mitochondria-targeted drug delivery by dual-ligand-functionalized nanodiamonds circumvent drug resistance. ACS Appl Mater Interfaces.

[497] Wang H, Zhang F, Wen H, Shi W, Huang Q, Huang Y, Xie J, Li P, Chen J, Qin L (2020). Tumor- and mitochondria-targeted nanoparticles eradicate drug resistant lung cancer through mitochondrial pathway of apoptosis. J Nanobiotechnol.

[498] Cho H, Cho Y-Y, Shim MS, Lee JY, Lee HS, Kang HC (2020). Mitochondria-targeted drug delivery in cancers. Biochim Biophys Acta Mol Basis Dis.

[499] Pfanner N, Warscheid B, Wiedemann N (2019). Mitochondrial proteins: from biogenesis to functional networks. Nat Rev Mol Cell Biol.

[500] Kreimendahl S, Schwichtenberg J, Günnewig K, Brandherm L, Rassow J (2020). The selectivity filter of the mitochondrial protein import machinery. BMC Biol.

[501] Baker MJ, Frazier AE, Gulbis JM, Ryan MT (2007). Mitochondrial protein-import machinery: correlating structure with function. Trends Cell Biol.

[502] Di Maio R, Barrett PJ, Hoffman EK, Barrett CW, Zharikov A, Borah A, Hu X, McCoy J, Chu CT, Burton EA, et al. α-Synuclein binds to TOM20 and inhibits mitochondrial protein import in Parkinson's disease. Sci Transl Med. 2016;8:342ra78. 10.1126/scitranslmed.aaf3634 PMC5016095 27280685

[503] Segui-Real B, Stuart RA, Neupert W (1992). Transport of proteins into the various subcompartments of mitochondria. FEBS Lett.

[504] Eisenberg-Bord M, Schuldiner M (2017). Ground control to major TOM: mitochondria-nucleus communication. FEBS J.

[505] Hartl FU, Neupert W (1990). Protein sorting to mitochondria: evolutionary conservations of folding and assembly. Science (New York, NY).

[506] Yu H, Mehta A, Wang G, Hauswirth WW, Chiodo V, Boye SL, Guy J (2013). Next-generation sequencing of mitochondrial targeted AAV transfer of human ND4 in mice. Mol Vis.

[507] Matissek KJ, Okal A, Mossalam M, Lim CS (2014). Delivery of a monomeric p53 subdomain with mitochondrial targeting signals from pro-apoptotic Bak or Bax. Pharm Res.

[508] Zhu X-J, Li R-F, Xu L, Yin H, Chen L, Yuan Y, Zhong W, Lin J (2019). A novel self-assembled mitochondria-targeting protein nanoparticle acting as theranostic platform for cancer. Small.

[509] Ahmed KS, Liu S, Mao J, Zhang J, Qiu L (2021). Dual-functional peptide driven liposome codelivery system for efficient treatment of doxorubicin-resistant breast cancer. Drug Des Devel Ther.

[510] Meade BR, Dowdy SF (2008). Enhancing the cellular uptake of siRNA duplexes following noncovalent packaging with protein transduction domain peptides. Adv Drug Deliv Rev.

[511] Snyder EL, Dowdy SF (2004). Cell penetrating peptides in drug delivery. Pharm Res.

[512] Cerrato CP, Pirisinu M, Vlachos EN, Langel Ü (2015). Novel cell-penetrating peptide targeting mitochondria. FASEB J..

[513] Cheng CJ, Saltzman WM (2011). Enhanced siRNA delivery into cells by exploiting the synergy between targeting ligands and cell-penetrating peptides. Biomaterials.

[514] Bai H, You Y, Yan H, Meng J, Xue X, Hou Z, Zhou Y, Ma X, Sang G, Luo X (2012). Antisense inhibition of gene expression and growth in gram-negative bacteria by cell-penetrating peptide conjugates of peptide nucleic acids targeted to rpoD gene. Biomaterials.

[515] Liu BR, Liou J-S, Chen Y-J, Huang Y-W, Lee H-J (2013). Delivery of nucleic acids, proteins, and nanoparticles by arginine-rich cell-penetrating peptides in rotifers. Mar Biotechnol (NY).

[516] Aguilera TA, Olson ES, Timmers MM, Jiang T, Tsien RY (2009). Systemic in vivo distribution of activatable cell penetrating peptides is superior to that of cell penetrating peptides. Integrative Biol..

[517] Bode SA, Wallbrecher R, Brock R, van Hest JCM, Löwik DWPM (2014). Activation of cell-penetrating peptides by disulfide bridge formation of truncated precursors. Chem Commun (Camb).

[518] Hansen MB, van Gaal E, Minten I, Storm G, van Hest JCM, Löwik DWPM (2012). Constrained and UV-activatable cell-penetrating peptides for intracellular delivery of liposomes. J Controlled Release.

[519] Kardani K, Milani A, H Shabani S, Bolhassani A. Cell penetrating peptides: the potent multi-cargo intracellular carriers. Expert Opinion Drug Delivery. 2019;16:1227–58. 10.1080/17425247.2019.1676720 31583914

[520] Khan MM, Filipczak N, Torchilin VP (2021). Cell penetrating peptides: a versatile vector for co-delivery of drug and genes in cancer. J Controlled Release.

[521] Park SE, Sajid MI, Parang K, Tiwari RK (2019). Cyclic cell-penetrating peptides as efficient intracellular drug delivery tools. Mol Pharm.

[522] Wu J, Li J, Wang H, Liu C-B (2018). Mitochondrial-targeted penetrating peptide delivery for cancer therapy. Expert Opin Drug Deliv.

[523] Chen W, Jin M-J, Gao Z-G, Wang L-P, Piao H-F (2011). Preparation and in vitro evaluation of pH-sensitive TAT peptide conjugated micelles. Yao Xue Xue Bao..

[524] Liu D, Angelova A, Liu J, Garamus VM, Angelov B, Zhang X, Li Y, Feger G, Li N, Zou A (2019). Self-assembly of mitochondria-specific peptide amphiphiles amplifying lung cancer cell death through targeting the VDAC1-hexokinase-II complex. J Mater Chem B.

[525] Woldetsadik AD, Vogel MC, Rabeh WM, Magzoub M (2017). Hexokinase II-derived cell-penetrating peptide targets mitochondria and triggers apoptosis in cancer cells. FASEB J.

[526] Johnson LV, Walsh ML, Bockus BJ, Chen LB (1981). Monitoring of relative mitochondrial membrane potential in living cells by fluorescence microscopy. J Cell Biol.

[527] Wallace DC (2012). Mitochondria and cancer. Nat Rev Cancer..

[528] Samudio I, Fiegl M, Andreeff M (2009). Mitochondrial uncoupling and the Warburg effect: molecular basis for the reprogramming of cancer cell metabolism. Can Res.

[529] Qian K, Chen H, Qu C, Qi J, Du B, Ko T, Xiang Z, Kandawa-Schulz M, Wang Y, Cheng Z (2020). Mitochondria-targeted delocalized lipophilic cation complexed with human serum albumin for tumor cell imaging and treatment. Nanomed Nanotechnol Biol Med.

[530] Jiang Z, Liu H, He H, Yadava N, Chambers JJ, Thayumanavan S (2020). Anionic polymers promote mitochondrial targeting of delocalized lipophilic cations. Bioconjug Chem.

[531] Kelley SO, Stewart KM, Mourtada R (2011). Development of novel peptides for mitochondrial drug delivery: amino acids featuring delocalized lipophilic cations. Pharm Res.

[532] Modica-Napolitano JS, Aprille JR (2001). Delocalized lipophilic cations selectively target the mitochondria of carcinoma cells. Adv Drug Deliv Rev.

[533] Bernal SD, Lampidis TJ, Summerhayes IC, Chen LB (1982). Rhodamine-123 selectively reduces clonal growth of carcinoma cells in vitro. Science (New York, NY).

[534] Lampidis TJ, Bernal SD, Summerhayes IC, Chen LB (1983). Selective toxicity of rhodamine 123 in carcinoma cells in vitro. Can Res.

[535] Murphy MP (1997). Selective targeting of bioactive compounds to mitochondria. Trends Biotechnol.

[536] Boddapati SV, Tongcharoensirikul P, Hanson RN, D'Souza GGM, Torchilin VP, Weissig V (2005). Mitochondriotropic Liposomes J Liposome Res.

[537] Boddapati SV, D'Souza GGM, Erdogan S, Torchilin VP, Weissig V (2008). Organelle-targeted nanocarriers: specific delivery of liposomal ceramide to mitochondria enhances its cytotoxicity in vitro and in vivo. Nano Lett.

[538] Asin-Cayuela J, Manas A-RB, James AM, Smith RAJ, Murphy MP. Fine-tuning the hydrophobicity of a mitochondria-targeted antioxidant. FEBS Lett. 2004;571. 10.1016/j.febslet.2004.06.045 15280009

[539] Hardy M, Poulhés F, Rizzato E, Rockenbauer A, Banaszak K, Karoui H, Lopez M, Zielonka J, Vasquez-Vivar J, Sethumadhavan S (2014). Mitochondria-targeted spin traps: synthesis, superoxide spin trapping, and mitochondrial uptake. Chem Res Toxicol.

[540] Biswas S, Dodwadkar NS, Deshpande PP, Torchilin VP (2012). Liposomes loaded with paclitaxel and modified with novel triphenylphosphonium-PEG-PE conjugate possess low toxicity, target mitochondria and demonstrate enhanced antitumor effects in vitro and in vivo. J Control Release.

[541] Zhou J, Zhao W-Y, Ma X, Ju R-J, Li X-Y, Li N, Sun M-G, Shi J-F, Zhang C-X, Lu W-L (2013). The anticancer efficacy of paclitaxel liposomes modified with mitochondrial targeting conjugate in resistant lung cancer. Biomaterials.

[542] Sun X, Wong JR, Song K, Chen LB (1996). Anticarcinoma activity of a novel drug, 3-ethyl-3'-methyl-thiatelluracarbocyanine iodide (Te), a tellurium-containing cyanine targeted at mitochondria. Clin Cancer Res.

[543] Pan Y, Zhao S, Chen F (2021). The potential value of dequalinium chloride in the treatment of cancer: focus on malignant glioma. Clin Exp Pharmacol Physiol.

[544] Bailly C (2021). Medicinal applications and molecular targets of dequalinium chloride. Biochem Pharmacol.

[545] Wang X-X, Li Y-B, Yao H-J, Ju R-J, Zhang Y, Li R-J, Yu Y, Zhang L, Lu W-L (2011). The use of mitochondrial targeting resveratrol liposomes modified with a dequalinium polyethylene glycol-distearoylphosphatidyl ethanolamine conjugate to induce apoptosis in resistant lung cancer cells. Biomaterials.

[546] Wang H, Yin H, Yan F, Sun M, Du L, Peng W, Li Q, Feng Y, Zhou Y (2015). Folate-mediated mitochondrial targeting with doxorubicin-polyrotaxane nanoparticles overcomes multidrug resistance. Oncotarget.

[547] Lv W, Guo J, Ping Q, Song Y, Li J (2008). Comparative pharmacokinetics of breviscapine liposomes in dogs, rabbits and rats. Int J Pharm.

[548] Chen C-L, Chang S-F, Lee D, Yang L-Y, Lee Y-H, Hsu CY, Lin S-J, Liaw J (2008). Bioavailability effect of methylprednisolone by polymeric micelles. Pharm Res.

[549] Mu L-M, Ju R-J, Liu R, Bu Y-Z, Zhang J-Y, Li X-Q, Zeng F, Lu W-L (2017). Dual-functional drug liposomes in treatment of resistant cancers. Adv Drug Deliv Rev.

[550] Francesko A, Petkova P, Tzanov T (2018). Hydrogel dressings for advanced wound management. Curr Med Chem.

[551] Huang H-J, Tsai Y-L, Lin S-H, Hsu S-H (2019). Smart polymers for cell therapy and precision medicine. J Biomed Sci.

[552] Farouk F, Shamma R (2019). Chemical structure modifications and nano-technology applications for improving ADME-Tox properties, a review. Arch Pharm.

[553] Lim D, Jung WC, Jeong J-H, Song M (2020). Targeted delivery of the mitochondrial target domain of noxa to tumor tissue via synthetic secretion system in *E. coli*. Front Bioeng Biotechnol.

[554] Luo X, Gong X, Su L, Lin H, Yang Z, Yan X, Gao J (2021). Activatable mitochondria-targeting organoarsenic prodrugs for bioenergetic cancer therapy. Angew Chem Int Ed Engl.

[555] Cheng G, Hardy M, Topchyan P, Zander R, Volberding P, Cui W, Kalyanaraman B (2020). Potent inhibition of tumour cell proliferation and immunoregulatory function by mitochondria-targeted atovaquone. Sci Rep.

[556] Ma J, Li L, Yue K, Li Y, Liu H, Wang PG, Wang C, Wang J, Luo W, Xie S (2020). Bromocoumarinplatin, targeting simultaneously mitochondria and nuclei with p53 apoptosis pathway to overcome cisplatin resistance. Bioorg Chem.

[557] Xing Y, Jiang Z, Akakuru OU, He Y, Li A, Li J, Wu A (2020). Mitochondria-targeting zeolitic imidazole frameworks to overcome platinum-resistant ovarian cancer. Colloids Surf B Biointerfaces.

[558] Cheng L, Wang C, Feng L, Yang K, Liu Z (2014). Functional nanomaterials for phototherapies of cancer. Chem Rev.

[559] Karimi M, Sahandi Zangabad P, Baghaee-Ravari S, Ghazadeh M, Mirshekari H, Hamblin MR (2017). Smart nanostructures for cargo delivery: uncaging and activating by light. J Am Chem Soc.

[560] Zhang L, Wang D, Yang K, Sheng D, Tan B, Wang Z, Ran H, Yi H, Zhong Y, Lin H, et al. Mitochondria-targeted artificial "Nano-RBCs" for amplified synergistic cancer phototherapy by a single NIR irradiation. Adv Sci (Weinheim, Baden-Wurttemberg, Germany). 2018;5:1800049. 10.1002/advs.201800049 PMC6097143 30128231

[561] Smith AM, Mancini MC, Nie S (2009). Bioimaging: second window for in vivo imaging. Nat Nanotechnol.

[562] Hemmer E, Acosta-Mora P, Méndez-Ramos J, Fischer S (2017). Optical nanoprobes for biomedical applications: shining a light on upconverting and near-infrared emitting nanoparticles for imaging, thermal sensing, and photodynamic therapy. J Mater Chem B.

[563] Sun W, Lu Y, Gu Z (2014). Advances in anticancer protein delivery using micro-/ nanoparticles. Particle Particle Syst Charact.

[564] Li X, Lovell JF, Yoon J, Chen X (2020). Clinical development and potential of photothermal and photodynamic therapies for cancer. Nat Rev Clin Oncol.

[565] Chen S, Lei Q, Qiu W-X, Liu L-H, Zheng D-W, Fan J-X, Rong L, Sun Y-X, Zhang X-Z. Mitochondria-targeting "Nanoheater" for enhanced photothermal/chemo-therapy. Biomaterials. 2017;117. 10.1016/j.biomaterials.2016.11.056 27939904

[566] Tao W, Zhu X, Yu X, Zeng X, Xiao Q, Zhang X, Ji X, Wang X, Shi J, Zhang H, et al. Black phosphorus nanosheets as a robust delivery platform for cancer theranostics. Adv Mater (Deerfield Beach, Fla). 2017;29. 10.1002/adma.201603276 PMC5205548 27797119

[567] Jung HS, Verwilst P, Sharma A, Shin J, Sessler JL, Kim JS (2018). Organic molecule-based photothermal agents: an expanding photothermal therapy universe. Chem Soc Rev.

[568] Chen S, Zhao X, Chen J, Chen J, Kuznetsova L, Wong SS, Ojima I. Mechanism-based tumor-targeting drug delivery system. Validation of efficient vitamin receptor-mediated endocytosis and drug release. Bioconjugate Chem. 2010;21:979–87. 10.1021/bc9005656 PMC3036843 20429547

[569] Marrache S, Dhar S (2015). The energy blocker inside the power house: mitochondria targeted delivery of 3-bromopyruvate. Chem Sci.

[570] Kang JH, Ko YT (2019). Dual-selective photodynamic therapy with a mitochondria-targeted photosensitizer and fiber optic cannula for malignant brain tumors. Biomater Sci.

[571] Nagashima C (1986). Multiple surgery of recurrent intramedullary spinal cord tumors. No shinkei geka Neurological Surg.

[572] Cerman E, Çekiç O (2015). Clinical use of photodynamic therapy in ocular tumors. Surv Ophthalmol.

[573] Ming H, Li B, Tian H, Zhou L, Jiang J, Zhang T, Qiao L, Wu P, Nice EC, Zhang W, He W, Huang C, Zhang H (2022). A minimalist and robust chemo-photothermal nanoplatform capable of augmenting autophagy-modulated immune response against breast cancer. Mater Today Bio..

[574] Hou H, Huang X, Wei G, Xu F, Wang Y, Zhou S (2019). Fenton reaction-assisted photodynamic therapy for cancer with multifunctional magnetic nanoparticles. ACS Appl Mater Interfaces.

[575] Zeng L, Pan Y, Tian Y, Wang X, Ren W, Wang S, Lu G, Wu A. Doxorubicin-loaded NaYF4:Yb/Tm-TiO2 inorganic photosensitizers for NIR-triggered photodynamic therapy and enhanced chemotherapy in drug-resistant breast cancers. Biomaterials. 2015;57. 10.1016/j.biomaterials.2015.04.006 25913254

[576] Zhang C, Bu W, Ni D, Zhang S, Li Q, Yao Z, Zhang J, Yao H, Wang Z, Shi J (2016). Synthesis of iron nanometallic glasses and their application in cancer therapy by a localized fenton reaction. Angew Chem Int Ed Engl.

[577] Sharker SM, Kim SM, Kim SH, In I, Lee H, Park SY (2015). Target delivery of β-cyclodextrin/paclitaxel complexed fluorescent carbon nanoparticles: externally NIR light and internally pH sensitive-mediated release of paclitaxel with bio-imaging. J Mater Chem B.

[578] Shi C-E, You C-Q, Pan L (2019). Facile formulation of near-infrared light-triggered hollow mesoporous silica nanoparticles based on mitochondria targeting for on-demand chemo/photothermal/photodynamic therapy. Nanotechnology.

[579] Yang G-G, Pan Z-Y, Zhang D-Y, Cao Q, Ji L-N, Mao Z-W (2020). Precisely assembled nanoparticles against cisplatin resistance via cancer-specific targeting of mitochondria and imaging-guided chemo-photothermal therapy. ACS Appl Mater Interfaces.

[580] Cheng H, Zheng R-R, Fan G-L, Fan J-H, Zhao L-P, Jiang X-Y, Yang B, Yu X-Y, Li S-Y, Zhang X-Z. Mitochondria and plasma membrane dual-targeted chimeric peptide for single-agent synergistic photodynamic therapy. Biomaterials. 2019;188. 10.1016/j.biomaterials.2018.10.005 30312907

[581] Liu J, Liang H, Li M, Luo Z, Zhang J, Guo X, Cai K (2018). Tumor acidity activating multifunctional nanoplatform for NIR-mediated multiple enhanced photodynamic and photothermal tumor therapy. Biomaterials.

[582] Chen H, Wang Y, Yao Y, Qiao S, Wang H, Tan N (2017). Sequential delivery of cyclopeptide RA-V and doxorubicin for combination therapy on resistant tumor and monitoring of cytochrome c release. Theranostics.

[583] Xiao Q, Dong X, Yang F, Zhou S, Xiang M, Lou L, Yao SQ, Gao L (2021). Engineered cell-penetrating peptides for mitochondrion-targeted drug delivery in cancer therapy. Chemistry (Weinheim an Der Bergstrasse, Germany).

[584] Shi J-F, Sun M-G, Li X-Y, Zhao Y, Ju R-J, Mu L-M, Yan Y, Li X-T, Zeng F, Lu W-L (2015). A combination of targeted sunitinib liposomes and targeted vinorelbine liposomes for treating invasive breast cancer. J Biomed Nanotechnol.

[585] He L, Zhang M-F, Pan Z-Y, Wang K-N, Zhao Z-J, Li Y, Mao Z-W (2019). A mitochondria-targeted iridium(iii)-based photoacid generator induces dual-mode photodynamic damage within cancer cells. Chem Commun (Camb).

[586] Song X-D, Kong X, He S-F, Chen J-X, Sun J, Chen B-B, Zhao J-W, Mao Z-W (2017). Cyclometalated iridium(III)-guanidinium complexes as mitochondria-targeted anticancer agents. Eur J Med Chem.

[587] Cheng Y, Ji Y (2020). Mitochondria-targeting nanomedicine self-assembled from GSH-responsive paclitaxel-ss-berberine conjugate for synergetic cancer treatment with enhanced cytotoxicity. J Control Release.

[588] Yan J, Chen J, Zhang N, Yang Y, Zhu W, Li L, He B (2020). Mitochondria-targeted tetrahedral DNA nanostructures for doxorubicin delivery and enhancement of apoptosis. J Mater Chem B.

[589] Orienti I, Salvati V, Sette G, Zucchetti M, Bongiorno-Borbone L, Peschiaroli A, Zolla L, Francescangeli F, Ferrari M, Matteo C (2019). A novel oral micellar fenretinide formulation with enhanced bioavailability and antitumour activity against multiple tumours from cancer stem cells. J Exp Clin Cancer Res: CR.

[590] He H, Wang J, Wang H, Zhou N, Yang D, Green DR, Xu B (2018). Enzymatic cleavage of branched peptides for targeting mitochondria. J Am Chem Soc.

[591] Ulger O, Kubat GB. Therapeutic applications of mitochondrial transplantation. Biochimie. 2022;195. 10.1016/j.biochi.2022.01.002 35026361

[592] Tachibana M, Sparman M, Sritanaudomchai H, Ma H, Clepper L, Woodward J, Li Y, Ramsey C, Kolotushkina O, Mitalipov S (2009). Mitochondrial gene replacement in primate offspring and embryonic stem cells. Nature.

[593] Craven L, Tuppen HA, Greggains GD, Harbottle SJ, Murphy JL, Cree LM, Murdoch AP, Chinnery PF, Taylor RW, Lightowlers RN (2010). Pronuclear transfer in human embryos to prevent transmission of mitochondrial DNA disease. Nature.

[594] Hyslop LA, Blakeley P, Craven L, Richardson J, Fogarty NME, Fragouli E, Lamb M, Wamaitha SE, Prathalingam N, Zhang Q (2016). Towards clinical application of pronuclear transfer to prevent mitochondrial DNA disease. Nature.

[595] Roushandeh AM, Kuwahara Y, Roudkenar MH (2019). Mitochondrial transplantation as a potential and novel master key for treatment of various incurable diseases. Cytotechnology.

[596] Zhao Z, Hou Y, Zhou W, Keerthiga R, Fu A (2021). Mitochondrial transplantation therapy inhibit carbon tetrachloride-induced liver injury through scavenging free radicals and protecting hepatocytes. Bioeng Transl Med.

[597] Moskowitzova K, Orfany A, Liu K, Ramirez-Barbieri G, Thedsanamoorthy JK, Yao R, Guariento A, Doulamis IP, Blitzer D, Shin B (2020). Mitochondrial transplantation enhances murine lung viability and recovery after ischemia-reperfusion injury. Am J Physiol Lung Cell Mol Physiol.

[598] Roushandeh AM, Tomita K, Kuwahara Y, Jahanian-Najafabadi A, Igarashi K, Roudkenar MH, Sato T (2020). Transfer of healthy fibroblast-derived mitochondria to HeLa ρ and SAS ρ cells recovers the proliferation capabilities of these cancer cells under conventional culture medium, but increase their sensitivity to cisplatin-induced apoptotic death. Mol Biol Rep.

[599] Jiang X-P, Elliott RL, Head JF (2015). Exogenous normal mammary epithelial mitochondria suppress glycolytic metabolism and glucose uptake of human breast cancer cells. Breast Cancer Res Treat.

[600] Sun C, Liu X, Wang B, Wang Z, Liu Y, Di C, Si J, Li H, Wu Q, Xu D (2019). Endocytosis-mediated mitochondrial transplantation: transferring normal human astrocytic mitochondria into glioma cells rescues aerobic respiration and enhances radiosensitivity. Theranostics.

[601] Vazquez F, Lim J-H, Chim H, Bhalla K, Girnun G, Pierce K, Clish CB, Granter SR, Widlund HR, Spiegelman BM (2013). PGC1α expression defines a subset of human melanoma tumors with increased mitochondrial capacity and resistance to oxidative stress. Cancer Cell.

[602] Henkenius K, Greene BH, Barckhausen C, Hartmann R, Märken M, Kaiser T, Rehberger M, Metzelder SK, Parak WJ, Neubauer A (2017). Maintenance of cellular respiration indicates drug resistance in acute myeloid leukemia. Leuk Res.

[603] Farge T, Saland E, de Toni F, Aroua N, Hosseini M, Perry R, Bosc C, Sugita M, Stuani L, Fraisse M (2017). Chemotherapy-resistant human acute myeloid leukemia cells are not enriched for leukemic stem cells but require oxidative metabolism. Cancer Discov.

[604] Zhan X-K, Li J-L, Zhang S, Xing P-Y, Xia M-F (2018). Betulinic acid exerts potent antitumor effects on paclitaxel-resistant human lung carcinoma cells (H460) via G2/M phase cell cycle arrest and induction of mitochondrial apoptosis. Oncol Lett.

[605] van Ginkel PR, Sareen D, Subramanian L, Walker Q, Darjatmoko SR, Lindstrom MJ, Kulkarni A, Albert DM, Polans AS (2007). Resveratrol inhibits tumor growth of human neuroblastoma and mediates apoptosis by directly targeting mitochondria. Clin Cancer Res.

[606] Cheng G, Zhang Q, Pan J, Lee Y, Ouari O, Hardy M, Zielonka M, Myers CR, Zielonka J, Weh K (2019). Targeting lonidamine to mitochondria mitigates lung tumorigenesis and brain metastasis. Nat Commun.

[607] Chen W, Ouyang J, Liu H, Chen M, Zeng K, Sheng J, Liu Z, Han Y, Wang L, Li J, et al. Black phosphorus nanosheet-based drug delivery system for synergistic photodynamic/photothermal/chemotherapy of cancer. Adv Mater (Deerfield Beach, Fla). 2017;29. 10.1002/adma.201603864 27882622

[608] Nam J, Son S, Ochyl LJ, Kuai R, Schwendeman A, Moon JJ (2018). Chemo-photothermal therapy combination elicits anti-tumor immunity against advanced metastatic cancer. Nat Commun.

[609] Lu K, He C, Guo N, Chan C, Ni K, Lan G, Tang H, Pelizzari C, Fu Y-X, Spiotto MT (2018). Low-dose X-ray radiotherapy-radiodynamic therapy via nanoscale metal-organic frameworks enhances checkpoint blockade immunotherapy. Nat Biom Eng.

[610] Zeng J-Y, Zou M-Z, Zhang M, Wang X-S, Zeng X, Cong H, Zhang X-Z (2018). π-Extended benzoporphyrin-based metal-organic framework for inhibition of tumor metastasis. ACS Nano.

[611] Zhang L, Yi H, Song J, Huang J, Yang K, Tan B, Wang D, Yang N, Wang Z, Li X (2019). Mitochondria-targeted and ultrasound-activated nanodroplets for enhanced deep-penetration sonodynamic cancer therapy. ACS Appl Mater Interfaces.

[612] Cardoso AM, Morais CM, Cruz AR, Cardoso AL, Silva SG, do Vale ML, Marques EF, Pedroso de Lima MC, Jurado AS. Gemini surfactants mediate efficient mitochondrial gene delivery and expression. Mol Pharmaceutics. 2015;12:716–30. 10.1021/mp5005349 25634573

[613] Bae Y, Jung MK, Song SJ, Green ES, Lee S, Park H-S, Jeong SH, Han J, Mun JY, Ko KS (2017). Functional nanosome for enhanced mitochondria-targeted gene delivery and expression. Mitochondrion.

[614] Coutinho E, Batista C, Sousa F, Queiroz J, Costa D (2017). Mitochondrial gene therapy: advances in mitochondrial gene cloning, plasmid production, and nanosystems targeted to mitochondria. Mol Pharm.

[615] He T, Qin X, Jiang C, Jiang D, Lei S, Lin J, Zhu W-G, Qu J, Huang P (2020). Tumor pH-responsive metastable-phase manganese sulfide nanotheranostics for traceable hydrogen sulfide gas therapy primed chemodynamic therapy. Theranostics.

[616] Motterlini R, Otterbein LE (2010). The therapeutic potential of carbon monoxide. Nat Rev Drug Discovery.

[617] Kim S, Tachikawa T, Fujitsuka M, Majima T (2014). Far-red fluorescence probe for monitoring singlet oxygen during photodynamic therapy. J Am Chem Soc.

[618] Hu P, Wu T, Fan W, Chen L, Liu Y, Ni D, Bu W, Shi J (2017). Near infrared-assisted Fenton reaction for tumor-specific and mitochondrial DNA-targeted photochemotherapy. Biomaterials.

[619] Yang Z, Wang J, Liu S, Li X, Miao L, Yang B, Zhang C, He J, Ai S, Guan W (2020). Defeating relapsed and refractory malignancies through a nano-enabled mitochondria-mediated respiratory inhibition and damage pathway. Biomaterials.

[620] Zeng F, Qin H, Liu L, Chang H, Chen Q, Wu L, Zhang L, Wu Z, Xing D. Photoacoustic-immune therapy with a multi-purpose black phosphorus-based nanoparticle. Nano Res. 2020. 10.1007/s12274-020-3028-x PMC7455780 32904446

[621] Bosc C, Selak MA, Sarry J-E (2017). Resistance is futile: targeting mitochondrial energetics and metabolism to overcome drug resistance in cancer treatment. Cell Metab.

